# Atualização das Diretrizes Brasileiras de Valvopatias – 2020

**DOI:** 10.36660/abc.20201047

**Published:** 2020-10-13

**Authors:** Flavio Tarasoutchi, Marcelo Westerlund Montera, Auristela Isabel de Oliveira Ramos, Roney Orismar Sampaio, Vitor Emer Egypto Rosa, Tarso Augusto Duenhas Accorsi, Antonio de Santis, João Ricardo Cordeiro Fernandes, Lucas José Tachotti Pires, Guilherme S. Spina, Marcelo Luiz Campos Vieira, Paulo de Lara Lavitola, Walkiria Samuel Ávila, Milena Ribeiro Paixão, Tiago Bignoto, Dorival Júlio Della Togna, Evandro Tinoco Mesquita, William Antônio de Magalhães Esteves, Fernando Atik, Alexandre Siciliano Colafranceschi, Valdir Ambrósio Moises, Alberto Takeshi Kiyose, Pablo M. A. Pomerantzeff, Pedro A. Lemos, Fabio Sandoli de Brito, Clara Weksler, Carlos Manuel de Almeida Brandão, Robinson Poffo, Ricardo Simões, Salvador Rassi, Paulo Ernesto Leães, Ricardo Mourilhe-Rocha, José Luiz Barros Pena, Fabio Biscegli Jatene, Márcia de Melo Barbosa, Alexandre Abizaid, Henrique Barbosa Ribeiro, Fernando Bacal, Carlos Eduardo Rochitte, José Honório de Almeida Palma da Fonseca, Samira Kaissar Nasr Ghorayeb, Marcelo Antonio Cartaxo Queiroga Lopes, Salvador Vicente Spina, Ricardo H. Pignatelli, José Francisco Kerr Saraiva

**Affiliations:** 1 Universidade de São Paulo Faculdade de Medicina Instituto do Coração (Incor) do Hospital de Clínica São PauloSP Brasil Instituto do Coração (Incor) do Hospital de Clínica da Faculdade de Medicina da Universidade de São Paulo (HCFMUSP), São Paulo, SP – Brasil; 2 Hospital Pró-Cardíaco Rio de JaneiroRJ Brasil Hospital Pró-Cardíaco, Rio de Janeiro, RJ – Brasil; 3 Instituto Dante Pazzanese de Cardiologia São PauloSP Brasil Instituto Dante Pazzanese de Cardiologia, São Paulo, SP – Brasil; 4 Universidade Federal Fluminense Rio de JaneiroRJ Brasil Universidade Federal Fluminense, Rio de Janeiro, RJ – Brasil; 5 Universidade Federal de Minas Gerais Belo HorizonteMG Brasil Universidade Federal de Minas Gerais, Belo Horizonte, MG – Brasil; 6 Fundação Universitária de Cardiologia São PauloSP Brasil Fundação Universitária de Cardiologia (FUC), São Paulo, SP – Brasil; 7 Universidade Federal de São Paulo São PauloSP Brasil Universidade Federal de São Paulo (UNIFESP), São Paulo, SP – Brasil; 8 Universidade de São Paulo Faculdade de Medicina São PauloSP Brasil Faculdade de Medicina da Universidade de São Paulo (FMUSP), São Paulo, SP – Brasil; 9 Hospital Israelita Albert Einstein São PauloSP Brasil Hospital Israelita Albert Einstein, São Paulo, SP – Brasil; 10 Fundação Zerbini São PauloSP Brasil Fundação Zerbini, São Paulo, SP – Brasil; 11 Instituto Nacional de Cardiologia Rio de JaneiroRJ Brasil Instituto Nacional de Cardiologia, Rio de Janeiro, RJ – Brasil; 12 Faculdade Ciências Médicas de Minas Gerais Belo HorizonteMG Brasil Faculdade Ciências Médicas de Minas Gerais, Belo Horizonte, MG – Brasil; 13 Universidade Federal de Goiás GoiâniaGO Brasil Universidade Federal de Goiás, Goiânia, GO – Brasil; 14 Santa Casa de Misericórdia de Porto Alegre Porto AlegreRS Brasil Santa Casa de Misericórdia de Porto Alegre, Porto Alegre, RS – Brasil; 15 Hospital Universitário Pedro Ernesto Rio de JaneiroRJ Brasil Hospital Universitário Pedro Ernesto, Rio de Janeiro, RJ – Brasil; 16 Hospital Felício Rocho Belo HorizonteMG Brasil Hospital Felício Rocho, Belo Horizonte, MG – Brasil; 17 Hospital Socor Belo HorizonteMG Brasil Hospital Socor, Belo Horizonte, MG – Brasil; 18 Hospital Alberto Urquiza Wanderley João PessoaPB Brasil Hospital Alberto Urquiza Wanderley, João Pessoa, PB – Brasil; 19 Texas Children’s Hospital Houston EUA Texas Children’s Hospital, Houston – EUA; 20 Pontifícia Universidade Católica de Campinas Instrução Mantenedora CampinasSP Brasil Sociedade Campineira de Educação e Instrução Mantenedora da Pontifícia Universidade Católica de Campinas, Campinas, SP – Brasil

## 1. Introdução

**Figura 1:**
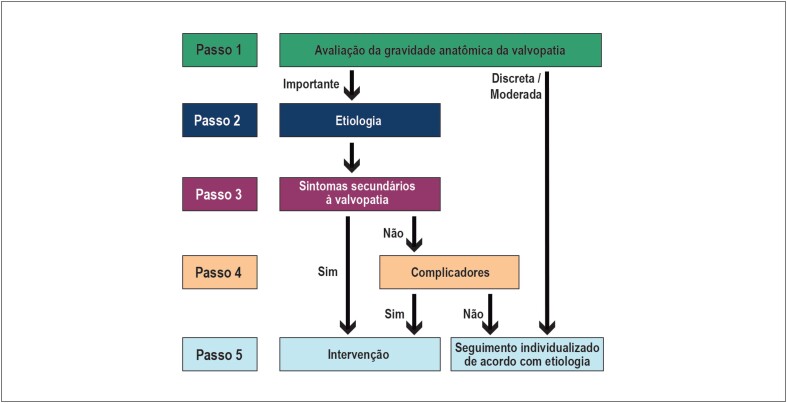
Algoritmo com passos para diagnóstico anatômico, etiológico e funcional, além da definição de conduta.

Atualmente, há grande variedade de estratégias intervencionistas - tanto transcateter, quanto cirúrgicas - que podem ser indicadas para pacientes portadores de valvopatia cardíaca, com objetivo de redução da morbimortalidade associada a esta doença. O correto momento de indicação e o tipo de tratamento intervencionista estão atrelados ao preciso diagnóstico anatômico e funcional da valvopatia cardíaca e a uma minuciosa avaliação global do paciente. Estas Diretrizes Brasileira de Valvopatias de 2020, além da compilação de evidências científicas e opinião de especialistas, mantém o ideal de ser extremamente útil ao apoio à decisão frente o paciente portador de valvopatia e tem três características que a diferencia:

- Manutenção do fluxograma inovador proposto na edição de 2017, com passos sequenciais que norteiam o diagnóstico anatômico, etiológico e funcional, definindo a conduta alinhada às melhores práticas, com uso racional de recursos (Figura 1);
- Aumento do número de recomendações a serem consultadas na tentativa de contemplar as múltiplas possibilidades frente o aumento da complexidade dos pacientes;
- Comparação das recomendações destas diretrizes com as principais diretrizes internacionais — American College of Cardiology/American Heart Association (ACC/AHA) 2017 e European Society of Cardiology/European Association for CardioThoracic Surgery (ESC/EACTS) 2017 – permitindo individualização da nossa população.^1^^,^^2^

Esta edição de 2020 contempla o raciocínio frente o paciente portador de valvopatia não importante e enfatiza a necessidade de ponderar a possibilidade de intervenção transcateter para idosos independente do risco presumido para cirurgia convencional e para todos pacientes com valva nativa ou prótese com risco presumido mais elevado para cirurgia convencional. A despeito de grandes avanços e maior disponibilização de exames de imagem, há manutenção da recomendação de avaliação clínica detalhada que ainda é imprescindível para diagnóstico, tomada de conduta e relacionamento médico-paciente.

Abaixo, os passos recomendados a serem seguidos:

- Primeiro passo: certificar-se de que a valvopatia é anatomicamente importante e, se confirmada, progredir para segundo passo. Caso não seja, procurar diagnósticos diferenciais em paciente sintomático e monitorizar evolução em paciente assintomático;
- Segundo passo: avaliar a etiologia, incluindo história clínica e antecedentes pessoais, em conjunção com exames complementares;
- Terceiro passo: avaliar os sintomas - fundamental na decisão de intervenção. O tratamento farmacológico está indicado para alívio dos sintomas até o tratamento intervencionista da doença valvar;
- Quarto passo: avaliação de complicadores - anatômicos e/ou funcionais (especialmente hipertensão pulmonar [HP], remodelamento ventricular, disfunção sistólica, dilatação aneurismática de aorta, fibrilação atrial [FA]). Pode ser determinante para intervenção nos pacientes assintomáticos;
- Quinto passo: tipo de intervenção - o procedimento de correção da doença valvar pode ser cirúrgico ou transcateter, com indicação individualizada dependendo do risco operatório, das comorbidades e da decisão do *Heart Team*.

## 2. O *Heart Team*

O *Heart Team* é um conceito no qual um conjunto de diferentes profissionais com experiência em doenças valvares compartilha a decisão sobre o tratamento mais adequado para um determinado paciente. Com a grande variedade de estratégias intervencionistas disponíveis para o tratamento do paciente portador de valvopatia, o *Heart Team* é fundamental para análise do risco/benefício e custo/eficácia de cada estratégia e auxílio à tomada de decisão. É composto por diversas subespecialidades cardiológicas, que exercerão papéis diferentes e fundamentais em cada passo do cuidado: desde o cardiologista clínico, a quem caberá a seleção e indicação de pacientes, além do acompanhamento pré e pós-intervenção, até o cirurgião cardíaco e o hemodinamicista, que serão os profissionais responsáveis pela concretização dos procedimentos indicados pelo *Heart Team*. Além destes, o radiologista será importante na análise de dados para avaliar a possibilidade técnica de realização de cada tipo de intervenção, e o ecocardiografista, além de avaliar os dados pré-operatórios poderá também acompanhar o procedimento, colaborando para um melhor resultado.^1^^,^^2^

## 3. Avaliação de Risco Operatório

A indicação da intervenção nos pacientes valvares deve ser sempre baseada na comparação do benefício e do provável risco do procedimento proposto. Para tal fim, alguns escores *online* são utilizados, dentre eles o EuroSCORE II (http://www.euroscore.org/calc.html) e o STS (*Society of Thoracic Surgeons*, http://riskcalc.sts.org/stswebriskcalc/#/calculate), validados em diferentes populações, com capacidade preditiva de mortalidade em 30 dias. Pacientes com STS < 4% são convencionalmente considerados de baixo risco cirúrgico, enquanto aqueles com escore entre 4-8% têm risco intermediário e aqueles com escore > 8% tem alto risco. Em relação ao EuroSCORE II, quando menor que 4% o paciente é considerado de baixo risco e, se escore > 4%, risco aumentado. Caso ocorra discrepância entre os escores, devemos utilizar aquele cujo risco estimado foi maior.^3^^–^^8^

Importante ressaltar que ambos os escores não incluem alguns fatores relacionados a desfechos prognósticos, como fragilidade e contraindicações específicas aos procedimentos, como, por exemplo, aorta em porcelana. Além disso, a avaliação do risco não substitui a impressão da avaliação clínica individual e a decisão da intervenção deve ser sempre compartilhada com paciente e familiares.

## 4. Fragilidade

Fragilidade é uma entidade que denota um estado de vulnerabilidade do idoso, associado à fraqueza física e baixa reserva fisiológica. Tem extrema relevância na avaliação individualizada devido principalmente a 2 fatores:

– é um preditor de eventos, como mortalidade, tempo de hospitalização e declínio funcional, após intervenção cirúrgica ou transcateter;
– não é contemplada nos escores de risco de uso rotineiro.

Vários escores e ferramentas estão disponíveis para a avaliação e quantificação da fragilidade, através da mensuração de dados relacionados ao status funcional, atividades instrumentais diárias, nutrição, cognição, independência para atividades, dentre outros. Importante que a avaliação da fragilidade não seja apenas subjetiva (*eyeball test*), mas sim um conjunto da impressão clínica associada a várias medidas/escores objetivos.^9^^–^^14^

## 5. Estenose Mitral

O exame físico é o primeiro recurso utilizado para avaliação anatômica da estenose mitral (EM). Pacientes com EM discreta a moderada poderão já apresentar estalido de abertura da valva mitral e sopro diastólico em ruflar mitral, com formato em decrescendo, com início logo após o estalido. Nos pacientes com ritmo sinusal, o sopro apresenta um reforço pré-sistólico no final da diástole. Entretanto, é nos pacientes com EM importante que as alterações propedêuticas são mais evidentes, assim como surgem as alterações eletrocardiográficas e radiológicas. Tais alterações presentes em pacientes com EM importante encontram-se no Quadro 1.

**Quadro 1:** Passo 1: Diagnóstico de estenose mitral importante^15^

	Características de estenose mitral importante
Exame físico	*Facies mitralis* Estalido de abertura precoce B1 hiperfonética B2 hiperfonética Sopro diastólico em ruflar, com reforço pré-sistólico se paciente em ritmo sinusal Sinais de congestão pulmonar e insuficiência cardíaca direita Presença de IT
Eletrocardiograma	Sobrecarga de AE Sobrecarga de câmaras direitas FA
Radiografia de tórax	Índice cardiotorácico normal Sinais de aumento de AE: Elevação do brônquio fonte esquerdo (“sinal da bailarina”) Duplo contorno atrial à direita 4° arco na silhueta cardíaca à esquerda Sinais de congestão pulmonar
Ecocardiograma	AVM < 1,5 cm^2^ Gradiente diastólico médio AE/VE ≥ 10 mmHg PSAP ≥ 50 mmHg em repouso PSAP ≥ 60 mmHg com esforço
Estudo hemodinâmico	Indicado em caso de discordância entre achados clínicos e ecocardiográficos Gradiente diastólico AE/VE ≥ 10 mmHg (espontâneo ou após prova com atropina e volume) PSAP ≥ 50 mmHg

AE: átrio esquerdo; AVM: área valvar mitral; FA: fibrilação atrial; IT: insuficiência tricúspide; PSAP: pressão sistólica da artéria pulmonar; VE: ventrículo esquerdo.

A ecocardiografia é o principal exame complementar para a avaliação anatômica da valva mitral, sendo fundamental para a definição da gravidade da valvopatia, das repercussões hemodinâmicas e dos parâmetros que estão relacionados à chance de sucesso das intervenções, com avaliação individualizada de cada componente da valva (anel valvar, cúspides valvares, aparato subvalvar).

Os parâmetros ecocardiográficos que classificam a gravidade da EM são a área valvar mitral (AVM), que pode ser aferida pela planimetria, pelo PHT (da sigla em inglês, *pressure half time*) ou pela equação de continuidade, e o gradiente diastólico transvalvar mitral.^15^

Do ponto de vista epidemiológico (Quadro 2), a EM segue apresentando como sua principal etiologia a febre reumática (FR), mantendo sua prevalência em países em desenvolvimento, inclusive no Brasil. Nestes países, a doença valvar reumática mantém uma estimativa de prevalência de 1 a 7 para cada 1000 crianças em estudos clínicos, podendo este número ser até 10 vezes maior com uso da ecocardiografia para *screening* populacional. Nos países desenvolvidos, as estatísticas apontam a EM como responsável por cerca de 9% do total das valvopatias nos países europeus, e apresentando uma prevalência de 0,1% nos Estados Unidos. Nestes países, a predominância dos casos ocorre em pacientes idosos e em imigrantes jovens, provenientes dos países em desenvolvimento.^16^^–^^18^

**Quadro 2:** Passo 2: Avaliação da etiologia da estenose mitral importante^16^^,^^17^

	Características etiológicas
Febre reumática	> 90% dos casos nos países em desenvolvimento Sintomas entre a 3ᵃ e 4ᵃ décadas da vida Fusão comissural, espessamento de cúspides Comprometimento do aparelho subvalvar Abertura em cúpula da cúspide anterior e redução da mobilidade da cúspide posterior Acometimento mitroaórtico
Degenerativa (calcificação do anel valvar)	12 a 26% dos casos nos países desenvolvidos Mais comum nos idosos Pode chegar a 60% dos casos em pacientes com mais de 80 anos de idade Calcificação do anel valvar mitral Ausência de fusão comissural Relação com calcificação aórtica e coronariana
Causas raras	Congênita Doenças reumatológicas (lúpus/artrite reumatoide) Drogas (metisergida/anorexígenos) Síndrome carcinóide Doença de Fabry Lesão actínica – pós-radioterapia

Além da etiologia reumática, cresce proporcionalmente o número de pacientes portadores de EM de etiologia degenerativa, ocasionada por calcificação do anel mitral, que pode se estender para a base dos folhetos valvares, gerando restrição para a movimentação das cúspides, com consequente restrição para o esvaziamento atrial. A prevalência estimada de calcificação do anel mitral está em torno de 10% na população idosa. Destes pacientes, cerca de 1 a 2% desenvolvem EM.^19^

Outras causas raras de EM incluem doenças reumatológicas (como lúpus eritematoso sistêmico ou artrite reumatoide), doenças de depósito (como doença de Fabry), doença de Whipple, terapia com metisergida ou anorexígenos, síndrome carcinóide ou alterações anatômicas congênitas da valva mitral, como valva mitral em paraquedas ou hipoplasia da valva mitral.

Nos pacientes com EM importante, é necessário estar atento ao possível surgimento de sintomas (Quadro 3), sendo o mais comum dispneia (classe funcional [CF] II a IV da *New York Heart Association* [NYHA]). Em particular, dispneia pode surgir em situações que levam ao aumento da pressão venocapilar pulmonar (esforço físico, gestação, fibrilação atrial). Com o passar do tempo, pode surgir mesmo em repouso, inclusive com ortopneia associada. Outros sintomas que podem se desenvolver são palpitações, hemoptise, disfonia, disfagia, tosse e eventos embólicos.

**Quadro 3:** Passo 3: Avaliação de sintomas na estenose mitral importante

	Sintomas
Dispneia (NYHA II – IV)	Principal sintoma Inicialmente com eventos que aumentam a pressão venocapilar pulmonar (esforço físico, fibrilação atrial, gestação) Dispneia em repouso e dispneia paroxística noturna Pode ser acompanhada por palpitações, hemoptise, disfonia, disfagia, tosse Pode ser acompanhada por eventos embólicos (cerebrais, mesentéricos, de extremidades)

Paralelamente à avaliação dos sintomas, o acompanhamento dos pacientes deverá incluir também a busca por possíveis complicadores (Quadro 4). No caso da EM importante, as alterações funcionais relevantes são a presença de HP significativa (pressão sistólica da artéria pulmonar –PSAP maior que 50 mmHg em repouso ou maior que 60mmHg no esforço) ou FA de início recente (desencadeada nos últimos meses).

**Quadro 4:** Passo 4: Avaliação de complicadores da estenose mitral importante

	Complicadores
Hipertensão pulmonar	PSAP ≥ 50 mmHg em repouso PSAP ≥ 60 mmHg ao esforço (teste ergométrico ou ecocardiografia com estresse farmacológico)
FA de início recente	Relação com remodelamento do AE Manter INR 2,0 a 3,0

INR: razão normalizada internacional; PSAP: pressão sistólica da artéria pulmonar; AE: átrio esquerdo; FA: fibrilação atrial; INR: razão normalizada internacional; PSAP: pressão sistólica da artéria pulmonar.

**Figura 2:**
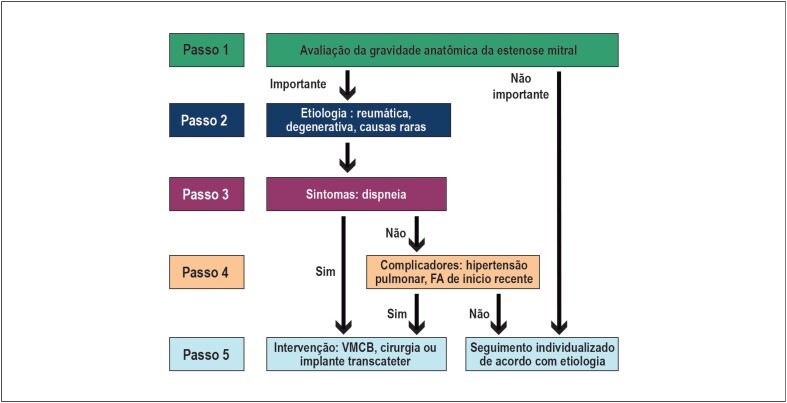
Algoritmo para tomada de decisão na estenose mitral. FA: fibrilação atrial; VMCB: valvoplastia mitral por cateter-balão.

Os tipos de intervenção disponíveis e as indicações das referidas intervenções estão descritas nos Quadros 5 e 6 e Figura 2. A valvoplastia mitral por cateter-balão (VMCB) segue sendo o tratamento de escolha para os pacientes com EM de etiologia reumática, nos quais predomina a calcificação e fusão comissurais, desde que apresentem anatomia valvar favorável (pela avaliação do escore de Wilkins-Block – Quadro 7), e na ausência de contraindicações (insuficiência mitral [IM] moderada a importante e trombo em AE). O escore de Wilkins-Block consiste na avaliação ecocardiográfica da valva mitral com ênfase na descrição dos aspectos estruturais. Quatro parâmetros são considerados: mobilidade dos folhetos, espessamento valvar, grau de calcificação e acometimento do aparato subvalvar. Uma graduação de um a quatro pontos para cada item resulta num escore que pode variar de 4 a 16 pontos. Pacientes com escore de Wilkins-Block inferior ou igual a 8 são candidatos a VMCB, na ausência de outras contraindicações. Nos pacientes mais sintomáticos (NYHA III ou IV) ou com complicadores e que apresentem anatomia desfavorável à VMCB ou contraindicações ao procedimento percutâneo, o tratamento cirúrgico da valva mitral passa a ser a opção terapêutica de escolha. A cirurgia poderá consistir na comissurotomia mitral ou, nos casos em que há comprometimento valvar muito significativo, sem possibilidade de manutenção da valva nativa, na troca valvar por prótese biológica ou mecânica.^20^^,^^21^

**Quadro 5:** Passo 5: Tipo de intervenção na estenose mitral importante^15^^,^^17^^,^^20^^–^^25^

Tipo	Considerações
Valvoplastia mitral por cateter-balão	Tratamento de escolha na etiologia reumática Indicações: sintomas (CF II-IV) e/ou fatores complicadores escore ecocardiográfico de Wilkins-Block ≤ 8 * (aparelho subvalvar e calcificação ≤ 2) Em gestantes ou pacientes com alto risco cirúrgico, considerar se: escore ecocardiográfico 9-10 (aparelho subvalvar e calcificação ≤ 2) Contraindicações: – trombo em AE – IM moderada ou importante – fenômeno embólico recente
Tratamento cirúrgico (comissurotomia/troca valvar)	EM reumática CF III-IV com contraindicações à VMCB EM reumática com fatores complicadores, não elegíveis para VMCB EM degenerativa refratária ao tratamento clínico
Implante valvar mitral transcateter (*valve-in-MAC*)	EM degenerativa refratária ao tratamento clínico, com contraindicação ou alto risco ao tratamento cirúrgico (em estudo)

* Individualizar em casos de escore ecocardiográfico 9-10. Pacientes com calcificação e aparato subvalvar com pontuações menores que 3 têm maiores taxas de sucesso com VMCB. AE: átrio esquerdo; CF: classe funcional; EM: estenose mitral; IM: insuficiência mitral; MAC: mitral annulus calcificatio; VMCB: valvoplastia mitral por cateter-balão.

**Quadro 6:** Estenose mitral: Recomendações^1^^,^^2^^,^^15^^,^^17^^,^^20^^–^^25^

Intervenção	Condição clínica	SBC	AHA	ESC
Valvoplastia mitral por cateter-balão	EM reumática CF II-IV, na ausência de contraindicações	I A	I A	I B
	EM reumática assintomática, com fatores complicadores, na ausência de contraindicações	I C	IIb C (se FA)	IIa C (se alto risco tromboembólico ou de deterioração hemodinâmica)
Tratamento cirúrgico (comissurotomia/troca valvar)	EM reumática CF III-IV com contraindicações à VMCB	I B	I B	I C
	EM reumática assintomático com fatores complicadores, não elegíveis para VMCB	IIa C	IIb C (Embolia recorrente)	–
	EM degenerativa refratária ao tratamento clínico	IIb C*	–	–
	EM reumático assintomático em programação de outra cirurgia cardíaca	I C	I C	–
Implante valvar mitral transcateter (valve-in-MAC)	EM degenerativa refratária ao tratamento clínico, com contraindicação ou alto risco a tratamento cirúrgico	IIb C*	–	–

* Considerar discussão junto ao Heart Team. AHA: American Heart Association; CF: classe funcional; EM: estenose mitral; ESC: European Society of Cardiology; MAC: mitral annulus calcificatio; SBC: Sociedade Brasileira de Cardiologia; VMCB: valvoplastia mitral por cateter-balão.

**Quadro 7:** Escore ecocardiográfico de Wilkins-Block

Mobilidade dos folhetos: Mobilidade elevada da valva com apenas restrição nas extremidades dos folhetos Regiões medial e basal apresentam mobilidade normal A valva continua se movendo adiante na diástole, principalmente na base Nenhum ou mínimo movimento dos folhetos em diástole
Acometimento subvalvar: Mínimo espessamento subvalvar exatamente abaixo dos folhetos mitrais Espessamento de cordas estendendo-se por mais de um terço do comprimento Espessamento expandindo-se para o terço distal das cordas Espessamento extenso e encurtamento de todas as estruturas das cordas expandindo-se para os músculos papilares
Espessura dos folhetos: Espessamento dos folhetos com espessura próxima do normal (4-5 mm) Camadas médias normais, espessamento considerável de margens (5-8 mm) Espessamento expandindo através de toda a camada (5-8 mm) Espessamento considerável de toda a camada do tecido (> 8-10 mm)
Calcificação valvar: Uma área única da ecoluminosidade aumentada Mínimas áreas de luminosidade confinadas às margens do folheto Luminosidade expandindo-se dentro da porção média dos folhetos Luminosidade extensa, além dos limites dos folhetos

No caso dos pacientes com EM degenerativa, por outro lado, a VMCB não é uma opção terapêutica, uma vez que não há fusão comissural, e sim calcificação do anel valvar, podendo ou não se estender para a base dos folhetos. Além disso, nestes pacientes, que habitualmente são mais idosos e frequentemente portadores de múltiplas comorbidades, o risco cirúrgico é significativamente mais elevado. O procedimento cirúrgico é acompanhado de dificuldades técnicas que podem aumentar as chances de complicações, incluindo disjunção atrioventricular, lesão da artéria circunflexa e sangramento da parede ventricular. Desta forma, o tratamento inicial de escolha é clínico, com controle de frequência cardíaca com betabloqueador, bloqueador de canal de cálcio ou ivabradina (quando em ritmo sinusal e não tolerar medicações anteriores), associado a diurético.^22^ Caso haja controle adequado dos sintomas com esta estratégia, o paciente poderá permanecer sem indicação de novas intervenções. Para os pacientes refratários ao tratamento clínico, entretanto, faz-se necessário considerar a possibilidade de intervenção cirúrgica, nos casos de risco baixo a moderado, ou do eventual implante de prótese mitral por via transcateter. Nestes casos, o implante transcateter ocorre utilizando como apoio para a prótese valvar a calcificação importante do anel mitral, sendo o procedimento habitualmente referido na literatura em língua inglesa como *valve-in-MAC (mitral annulus calcification)*. Há ainda uma experiência limitada com este procedimento, realizado nos estudos clínicos mais frequentemente por via transeptal ou transapical. Apresenta ainda um alto índice de complicações, incluindo *leak paravalvar*, obstrução da via de saída do VE, embolização da prótese, e taxa de mortalidade que pode chegar a 25% em 30 dias e 54% em 12 meses. Requer, portanto, um número maior de estudos, que possibilitem uma menor taxa de complicações, para a expansão de suas indicações.^23^^–^^25^

O acompanhamento clínico do paciente, enquanto apresentar valvopatia anatomicamente não importante é realizado com consultas e reavaliações ecocardiográficas periódicas (Quadro 8). No paciente com EM não importante, reavaliações podem ser realizadas a cada 1 ano. Não é esperado rotineiramente que pacientes com área valvar ≥ 1,5 cm^2^ desenvolvam sintomas ou complicadores. No caso de surgimento destas alterações, antes que o paciente desenvolva valvopatia anatomicamente importante, é imperativo considerar a possibilidade de que outros diagnósticos diferenciais sejam responsáveis pelas mesmas. O paciente com EM importante, por sua vez, deverá ser reavaliado em intervalos menores de tempo, habitualmente a cada 6 a 12 meses.

**Quadro 8:** Estenose mitral: Acompanhamento individualizado^1^^,^^2^

Estenose mitral	Acompanhamento	SBC	AHA	ESC
Importante assintomático e sem complicadores	Reavaliação clínica e ecocardiográfica	A cada 6-12 meses	A cada 12 meses	A cada 12 meses
	Intervenção cirúrgica concomitante em pacientes que serão submetidos a outro procedimento cirúrgico cardíaco (revascularização coronária, aorta ascendente ou outra válvula)	I C	IIb C	–
Não importante (AVM > 1,5 cm² e gradiente médio AE/VE < 5 mmHg)	Reavaliação clínica e ecocardiográfica	A cada 1 ano	A cada 3-5 anos	A cada 2-3 anos

AE: átrio esquerdo; AHA: American Heart Association; AVM: área valvar mitral; ESC: European Society of Cardiology; SBC: Sociedade Brasileira de Cardiologia; VE: ventrículo esquerdo; VMCB: valvoplastia mitral por cateter-balão.

## 6. Insuficiência Mitral Primária Crônica

**Figura 3:**
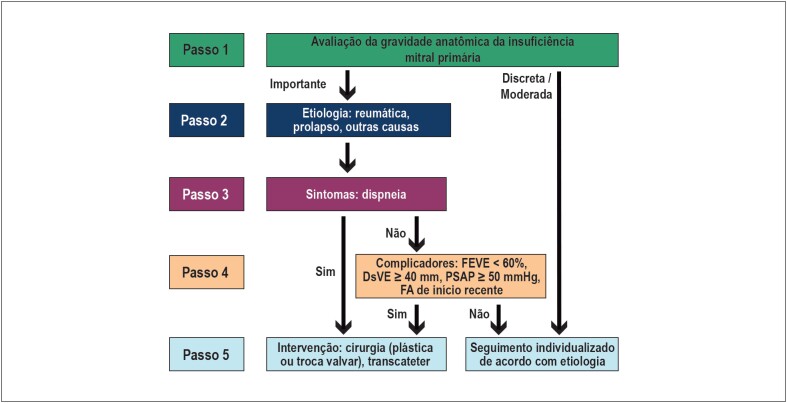
Algoritmo para tomada de decisão na insuficiência mitral crônica primária. DSVE: diâmetro sistólico do ventrículo esquerdo; FEVE: fração de ejeção do VE; PSAP: pressão sistólica da artéria pulmonar.

Para a tomada de decisão frente a um paciente com IM primária crônica, recomenda-se que sejam seguidos os 5 passos do algoritmo de abordagem das valvopatias, conforme detalhado abaixo e posteriormente resumido na Figura 3.

Além de confirmar a presença da valvopatia, o ecocardiograma transtorácico é o principal exame empregado para a definição da gravidade anatômica da IM. Diversos parâmetros podem ser utilizados para essa quantificação, sendo de fundamental importância um exame detalhado e completo (Quadro 9).

**Quadro 9:** Passo 1: Diagnóstico de insuficiência mitral primária importante^26^^–^^32^

	Características de insuficiência mitral primária importante
Exame físico	*Ictus cordis* desviado para a esquerda e para baixo B1 hipofonética (frequentemente audível em portadores de IM reumática e por prolapso e a perda da intensidade pode ser considerada um marcador de gravidade por disfunção ventricular, rotura cordoalha, dentre outros) B2 hiperfonética Sopro sistólico regurgitativo ≥ +++/6+ Sinais clínicos de insuficiência cardíaca direita
Eletrocardiograma	Sobrecarga de câmaras esquerdas Arritmias atriais ou ventriculares (extrassístoles, taquicardia) e FA
Radiografia de tórax	Aumento da silhueta cardíaca com dilatação do VE e AE Sinais de congestão pulmonar
Ecocardiograma	Área do jato ≥ 40% da área do AE Fração regurgitante ≥ 50% Volume regurgitante ≥ 60 mL/batimento *Vena contracta* ≥ 0,7 cm ERO ≥ 0,40 cm²
Estudo hemodinâmico	Indicado nos casos de dissociação clinicoecocardiográfica Ventriculografia esquerda (importante se > 3+) Avaliação de pressões intracavitárias
Ressonância Magnética	Casos de dissociação clinicoecocardiográfica ou qualidade limitada da imagem ecocardiográfica Confirmação da graduação da IM antes de intervenção programada na valva mitral Graduação da IM Avaliação da disjunção do anel mitral no complexo da doença mixomatosa e prolapso da valva mitral

AE: átrio esquerdo; ERO: área efetiva do orifício regurgitante; FA: fibrilação atrial; IM: insuficiência mitral; VE: ventrículo esquerdo.

Os pacientes com IM anatomicamente discreta ou moderada deverão ser mantidos em acompanhamento clínico e ecocardiográfico periódicos, sem indicação de intervenção (medicamentosa ou cirúrgica) para interrupção da história natural da doença valvar. Por outro lado, pacientes com IM importante devem prosseguir no fluxograma de avaliação específica, buscando-se sintomas secundários à valvopatia e/ou presença de complicadores.

Os pacientes com IM deverão ter definida a etiologia da valvopatia, uma vez que o seguimento clínico e a programação terapêutica (momento e tipo de intervenção), quando indicada, poderão ser diferentes conforme a causa da IM (Quadro 10). Apesar dos avanços nos métodos diagnósticos, o ecocardiograma transtorácico ainda é o primeiro e principal exame indicado para a quantificação anatômica e para a avaliação etiológica de pacientes com IM. ^33^^–^^35^

**Quadro 10:** Passo 2: Avaliação de etiologia na insuficiência mitral primária importante ^33^^–^^35^

	Características etiológicas
Reumática	Causa mais prevalente no Brasil Espessamento com retração das cúspides Acometimento comissural Acometimento mitroaórtico Frequente em adultos jovens
Prolapso da valva mitral e doenças associadas (“*flail*”, Barlow)	Segunda causa mais frequente no Brasil Protrusão de cúspides para o AE ≥ 2 mm Mais frequente na população de meia idade e idosa
Outras causas	Endocardite infecciosa Síndrome de Marfan Lúpus eritematoso sistêmico Lesões traumáticas Deformidades congênitas

AE: átrio esquerdo.

O principal sintoma apresentado pelos pacientes com IM anatomicamente importante é dispneia, a qual deve ser valorizada mesmo que ainda não limite as atividades habituais (CF II). Caso haja dúvida quanto à presença de sintomas, pode ser solicitado um teste ergométrico ou ergoespirométrico (Quadro 11). Uma vez confirmada a presença de sintomas, e sendo estes secundários à IM, os pacientes devem receber indicação de intervenção da valvopatia, conforme descrito no Passo 5 (Quadro 12).

**Quadro 11:** Passo 3: Avaliação de sintomas na insuficiência mitral primária importante

	Sintomas
Dispneia (CF II-IV) e fadiga/fraqueza	Congestão pulmonar Inicialmente com eventos que aumentam a pressão venocapilar pulmonar (esforço físico, FA, gestação) Dispneia em repouso e dispneia paroxística noturna Pode ser acompanhada por palpitações, tosse, edema Pode ser acompanhada por eventos embólicos

CF: classe funcional; FA: fibrilação atrial.

**Quadro 12:** Passo 5: Tipo de intervenção na insuficiência mitral primária importante ^39^^–^^52^

Tipo de intervenção	Considerações
Plástica da valva mitral	Tratamento de escolha Pacientes reumáticos: resultados menos favoráveis. Prolapso valvar mitral de cúspide posterior (P2 isolado): melhores resultados.
Troca da valva mitral	Indicada em caso de impossibilidade de plástica valvar.
Clipagem percutânea da valva mitral	Reservado a pacientes de alto risco ou com contraindicação cirúrgica com sintomas refratários IM degenerativa por prolapso Condição anatômica favorável Indicado após decisão do *Heart Team*

IM: insuficiência mitral.

Pacientes com IM importante assintomáticos devem ser periodicamente reavaliados quanto ao desenvolvimento ou não de alterações anatômicas e/ou funcionais secundárias à doença valvar (Quadro 13). Os complicadores associados à IM são: disfunção sistólica do ventrículo esquerdo (VE) (fração de ejeção do VE [FEVE] < 60%), dilatação do VE (diâmetro sistólico do VE [DSVE] ≥ 40 mm), HP (PSAP ≥ 50 mmHg em repouso ou ≥ 60mmHg ao esforço) e FA de início recente (desencadeada nos últimos meses).^36^^–^^38^ Aumento do volume atrial esquerdo (especialmente quando ≥ 60 ml/m^2^) pode ser considerado um complicador anatômico na IM, devendo ser levado em consideração na definição de conduta, uma vez que está associado a pior prognóstico. Além disso, caso haja queda progressiva da FEVE ou dilatação progressiva do VE em exames de imagem seriados, mesmo antes de atingidos os limites especificados acima, intervenção valvar mitral deve ser considerada.

**Quadro 13:** Passo 4: Avaliação de complicadores na insuficiência mitral primária importante^36^^–^^38^

	Complicadores
Ecocardiograma	FEVE ≤ 60% ou queda da FEVE durante a evolução (dentro da normalidade) Remodelamento progressivo (DSVE ≥ 40 mm) PSAP ≥ 50 mmHg ou ≥ 60 mmHg ao exercício Volume de AE ≥ 60 ml/m²
Eletrocardiograma	FA de início recente (< 1 ano)

AE: átrio esquerdo; DSVE: diâmetro sistólico do ventrículo esquerdo; FA: fibrilação atrial; FEVE: fração de ejeção do ventrículo esquerdo; PSAP: pressão sistólica da artéria pulmonar.

Após confirmada a presença de IM anatomicamente importante, analisada e definida a etiologia e, por fim, assegurada a presença de sintomas secundários à valvopatia e/ou de complicadores, o paciente sem contraindicação deverá receber indicação de intervenção da valvopatia (Quadro 13 e 14). Nesses casos, a cirurgia de plástica mitral é o tratamento de escolha, caso a etiologia (principalmente prolapso) e a anatomia sejam favoráveis, e o procedimento seja realizado em hospital capacitado e com cirurgião experiente. Em caso contrário, está indicada a cirurgia de troca valvar mitral.^39^^–^^52^

**Quadro 14:** Insuficiência mitral primária: Recomendações^1^^,^^2^^,^^39^^–^^52^

Intervenção	Condição clínica	SBC	AHA	ESC
Plástica da valva mitral (centros com experiência)	**Reumáticos**			
	Sintomático (CF ≥ II)	IIb C	IIb C	–
	Assintomático, com complicadores: – FEVE entre 30 e 60% e/ou DSVE ≥ 40 mm – PSAP ≥ 50 mmHg ou FA	IIb B	IIb B	–
		IIb B	–	–
	IM reumática, assintomática, sem complicadores	III	–	–
	**Não reumáticos**			
	CF ≥ II, com anatomia favorável	I B	I B	I B
	Assintomático, com anatomia favorável e com complicadores: – FEVE entre 30 e 60% e/ou DSVE ≥ 40 mm – PSAP ≥ 50 mmHg ou FA	I B	I B	I B (DSVE≥45 mm)
		IIa B	IIa B	IIa B
	Assintomático, IM por prolapso, com anatomia favorável, sem complicadores	IIa B	IIa B	IIa C (AE ≥ 60 ml/m² e ritmo sinusal)
Troca da valva mitral	**Reumáticos**			
	Sintomáticos (CF ≥ II)	I B	–	–
	Assintomático, com complicadores: – FEVE entre 30 e 60% e/ou DSVE ≥ 40 mm – PSAP ≥ 50 mmHg ou FA	I B	–	–
		IIa B	–	–
	IM reumática, assintomática, sem complicadores	III	–	–
	**Não reumáticos**			
	CF ≥ II, com anatomia desfavorável à plástica valvar	I B	I B	I B
	Assintomático, com anatomia desfavorável à plástica valvar, com complicadores: – FEVE entre 30 e 60% e DSVE ≥ 40mm – PSAP ≥ 50 mmHg ou FA	I B	I B	I C (DSVE≥45 mm)
		IIa C	IIa C	IIa B
	Assintomático, IM por prolapso, com anatomia desfavorável à plástica valvar, sem complicadores	III	III	III
Clipagem percutânea da valva mitral	IM não reumática, com alto risco ou contraindicação a cirurgia, com sintomas refratários	IIa B *	IIb B	IIb C

* Em centros com Heart Team. AHA: American Heart Association; CF: classe funcional; DSVE: diâmetro sistólico do ventrículo esquerdo; ESC: European Society of Cardiology; FA: fibrilação atrial; FEVE: fração de ejeção do VE; IM: insuficiência mitral; PSAP: pressão sistólica da artéria pulmonar; SBC: Sociedade Brasileira de Cardiologia.

Intervenções transcateter têm indicação restrita para pacientes com IM primária e devem ser decididas após discussão com o *Heart Team*. Da mesma forma, pacientes com contraindicação ou que apresentem elevado risco associado à cirurgia convencional, devem ser previamente discutidos com o *Heart Team* antes de terem sua conduta definida.

Quando, apesar da presença de IM anatomicamente importante, o paciente não apresentar sintomas nem complicadores, este deverá ser seguido de maneira individualizada, com acompanhamento clínico semestral e avaliação ecocardiográfica com intervalo máximo de 1 ano (Quadro 15).

**Quadro 15:** Insuficiência mitral primária: Acompanhamento individualizado^1^^,^^2^

Insuficiência mitral primária	Acompanhamento	SBC	AHA	ESC
Importante assintomático e sem complicadores	Reavaliação clínica e ecocardiográfica	A cada 6 meses a 1 ano	A cada 6 meses a 1 ano	A cada 6 meses
	Intervenção concomitante em pacientes que serão submetidos a outro procedimento cirúrgico cardíaco (revascularização coronária, aorta ascendente ou outra válvula)	I B	I B	–
Moderada (Área do jato 20- 40% da área do AE, Fração regurgitante 30-49%, Volume regurgitante 30-59 mL/batimento, Vena contracta 0,3-0,69 cm, ERO 0,2-0,39 cm²)	Reavaliação clínica e ecocardiográfica	A cada 1-2 anos	A cada 1-2 anos	A cada 1-2 anos
	Intervenção concomitante em pacientes que serão submetidos a outro procedimento cirúrgico cardíaco (revascularização coronária, aorta ascendente ou outra válvula)	IIa C	IIa C	–
Discreta (Área do jato < 20% da área do AE, Fração regurgitante < 30%, Volume regurgitante < 30 mL/batimento, Vena contracta < 0,3 cm, ERO < 0,2-0,39mm²)	Reavaliação clínica e ecocardiográfica	A cada 2-3 anos	A cada 3-5 anos	–

AE: átrio esquerdo; AHA: American Heart Association; ERO: área efetiva do orifício regurgitante; ESC: European Society of Cardiology; SBC: Sociedade Brasileira de Cardiologia.

Por outro lado, pacientes com IM anatomicamente moderada devem ter avaliação clínica anual e realizar ecocardiograma a cada 2 anos.

## 7. Insuficiência Mitral Secundária

A IM secundária decorre de alterações ventriculares (disfunção e/ou dilatação), enquanto que os folhetos valvares mitrais e as cordoalhas são normais. Nesse contexto, há uma sobrecarga adicional ao VE pela regurgitação mitral, culminando num pior prognóstico. As principais etiologias são: doença arterial coronária (IM isquêmica) e miocardiopatia dilatada (dilatação anular e/ou mau posicionamento). Por esses motivos, o tratamento ideal é controverso, uma vez que a correção valvar não é curativa. De maneira geral, está indicada intervenção em pacientes que se mantêm sintomáticos, a despeito de tratamento medicamentoso otimizado. Mesmo assim, a decisão terapêutica deve ser individualizada e, sempre que possível, compartilhada com o *Heart Team.*^53^

Como muitas vezes o exame físico é frustro para o diagnóstico da IM secundária, o ecocardiograma transtorácico é exame fundamental. Há evidências de que limites menores da área do orifício regurgitante e do volume regurgitante estão associados a pior prognóstico, quando comparados com IM primária. Todavia, para quantificação da gravidade anatômica da IM secundária, os limites ecocardiográficos utilizados são os mesmos da IM primária. Em casos de dissociação clinicoecocardiográfica, a realização de estudo hemodinâmico com ventriculografia esquerda ou ressonância magnética podem ajudar na definição (Quadro 16).^27^^–^^32^^,^^54^

**Quadro 16:** Passo 1: Diagnóstico de insuficiência mitral secundária importante^27^^–^^32^^,^^54^

	Características de insuficiência mitral secundária importante
Exame físico	B1 hipofonética ou normofonética Sopro protomesossistólico ou holossistólico com irradiação para linha axilar
Eletrocardiograma	Sinais de sobrecarga de câmaras esquerdas Sinais sugestivos da patologia de base
Radiografia de tórax	Aumento da silhueta cardíaca por dilatação de câmaras esquerdas
Ecocardiograma	Quantificação da regurgitação*: – Fração regurgitante ≥ 50% – Volume regurgitante ≥ 60 mL/batimento – ERO ≥ 0,40 cm²
Estudo hemodinâmico	Dissociação clinicoecocardiográfica Graduação da IM pela ventriculografia esquerda
Ressonância Magnética	Dissociação clinicoecocardiográfica ou qualidade limitada da imagem ecocardiográfica Confirmação da graduação da IM antes de intervenção programada na valva mitral Graduação da IM

* Considerar a possibilidade de insuficiência mitral anatomicamente importante em presença de ERO entre 0,3-0,4 cm ² quando associado a disfunção sistólica importante. ERO: área efetiva do orifício regurgitante; IM: insuficiência mitral.

O ecocardiograma fornece as principais informações necessárias para se estabelecer a etiologia da IM secundária, especialmente pela análise de alterações do VE (Quadro 17). A cineangiocoronariografia, por sua vez, tem papel importante no diagnóstico de doença arterial coronária obstrutiva que pode ser causa de IM.^53^

**Quadro 17:** Passo 2: Avaliação da etiologia na insuficiência mitral secundária importante^53^

	Características etiológicas
Isquêmica	Alterações segmentares da contratilidade Disposição inadequada dos músculos papilares ou das cúspides valvares (“em tenda”, ou com tracionamento apical – *tethering* – e/ou por falha na coaptação das cúspides) Dilatação ou deformidade anular mitral Avaliação de coronárias pela cinecoronariografia Avaliação de viabilidade pela ressonância magnética de coração
Dilatada	Dilatação do anel valvar – dilatação ventricular Disfunção ventricular sistólica Disposição inadequada dos músculos papilares ou das cúspides valvares (“em tenda”, ou com tracionamento apical – *tethering* – e/ou por falha na coaptação das cúspides) Dissincronia ventricular Alteração no acoplamento mecânico atrioventricular

Exames para avaliação de viabilidade miocárdica (como ressonância nuclear magnética) podem ser úteis em pacientes com IM isquêmica, nos quais há programação de revascularização miocárdica.

O principal sintoma apresentado por pacientes com IM secundária é dispneia, a qual pode decorrer da disfunção ventricular esquerda e/ou da regurgitação mitral associada (Quadro 18).

**Quadro 18:** Passo 3: Avaliação de sintomas na insuficiência mitral secundária importante

	Sintomas
Dispneia e fadiga/fraqueza	Aumento da pressão diastólica final Congestão venocapilar pulmonar Pode ser acompanhada por palpitações, tosse, ascite, edema, dor torácica Pode ser acompanhada por eventos embólicos

Pacientes com sintomas importantes (CF NYHA III e IV) e persistentes, a despeito de tratamento otimizado para insuficiência cardíaca (incluindo terapia de ressincronização, quando indicada), devem ser considerados para intervenção de maneira individualizada.

Não há complicadores específicos para pacientes com IM secundária, uma vez que a origem do problema está na doença ventricular (Quadro 19). Todavia, caso haja agravamento da dilatação e/ou da disfunção do VE, sem fator causal aparente, a valvopatia mitral concomitante pode ser considerada responsável.^55^^,^^56^

**Quadro 19:** Passo 4: Avaliação de complicadores na insuficiência mitral secundária importante^55^^,^^56^

	Complicadores
Avaliação clinicoecocardiográfica	Agravamento das condições de base sem outras causas atribuíveis (elevação de PSAP, aumento dos diâmetros ventriculares, queda da FEVE) Sintoma refratário ao tratamento clínico otimizado

FEVE: fração de ejeção do ventrículo esquerdo.

A indicação de intervenção em pacientes com IM secundária é controversa (Quadros 20 e 21). Em pacientes com IM isquêmica, candidatos à cirurgia de revascularização miocárdica, a abordagem simultânea da valvopatia mitral deve ser considerada. Por outro lado, em pacientes sem indicação de revascularização, a abordagem cirúrgica isolada da IM está associada à elevada mortalidade, altas taxas de recorrência da IM e não há evidência de benefício em termos de sobrevida.^53^^,^^57^^–^^66^

**Quadro 20:** Passo 5: Tipo de intervenção na insuficiência mitral secundária importante^53^^,^^57^^–^^72^

Tipo	Considerações
Cirurgia (plástica ou troca valvar)	Troca ou plástica valvar + revascularização miocárdica quando indicada
Clipagem percutânea da valva mitral	Pode ser considerada após avaliação do *Heart Team,* principalmente em pacientes com FEVE ≥ 20% e DSVE < 70 mm

DSVE: diâmetro sistólico do ventrículo esquerdo; FEVE: fração de ejeção do ventrículo esquerdo.

**Quadro 21:** Insuficiência mitral secundária: Recomendações na insuficiência mitral secundária importante^1^^,^^2^^,^^53^^,^^57^^–^^72^

Intervenção	Condição clínica	SBC	AHA	ESC
Troca ou Plástica da valva mitral	**Isquêmica**			
	Sintomático (CF ≥ III) Revascularização associada	IIb B	IIb B	IIb C
		IIa B	IIa B	I C (FEVE > 30%) IIa C (FEVE < 30%)
	**Dilatada**			
	Sintomático (CF ≥ III)	IIb B	IIb B	IIb C
Clipagem percutânea da valva mitral	**Isquêmica**			
	Sintomas refratários (CF ≥ III), com alto risco ou contraindicação à cirurgia	IIa B	–	IIb C (FE < 30%)
	**Dilatada**			
	Sintomas refratários (CF ≥ III) com alto risco ou contraindicação à cirurgia	IIa B	–	IIb C (FE < 30%)

AHA: American Heart Association; CF: classe funcional; ESC: European Society of Cardiology; SBC: Sociedade Brasileira de Cardiologia.

Em pacientes com IM secundária a cardiomiopatia dilatada, a indicação de intervenção na valvopatia mitral é ainda mais restrita. Enquanto a cirurgia valvar mitral isolada não mostrou benefício nesse cenário, novas evidências mostraram benefício da intervenção transcateter em pacientes com IM secundária, FEVE ≥ 20% e sintomáticos a despeito de tratamento clínico otimizado, desde que o procedimento não seja indicado em fases mais avançadas da história natural da valvopatia. ^67^^–^^72^

**Figura 4:**
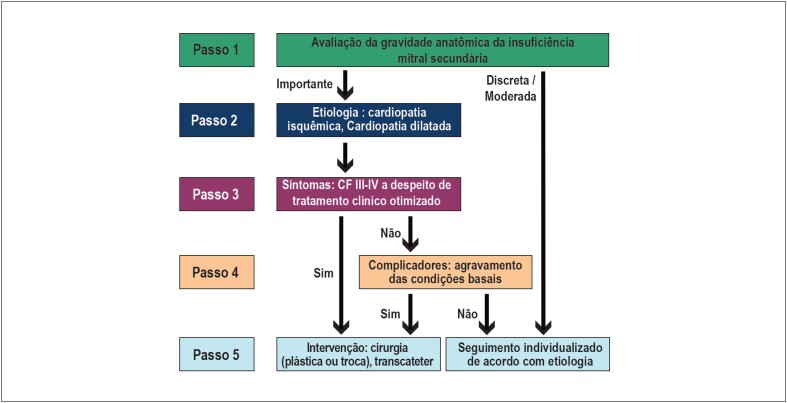
Algoritmo para tomada de decisão na insuficiência mitral secundária. CF: classe funcional.

Para indicação mais adequada e abordagem mais completa, os casos de IM secundária devem ser discutidos com o *Heart Team* antes da tomada de decisão (Figura 4).

## 8. Estenose Aórtica

A estenose aórtica (EAo) apresenta prevalência crescente na atualidade em razão do aumento da expectativa de vida e do consequente envelhecimento da população brasileira. Atualmente, a causa mais comum de EAo é a calcificação/degeneração aórtica, que acomete principalmente pacientes idosos. O tratamento transcateter tornou-se uma opção à troca valvar cirúrgica não só em pacientes frágeis e de alto risco, mas também nos outros extratos de risco operatório. Dessa maneira, o *Heart Team* torna-se cada vez mais importante e necessário para a decisão da intervenção em tais pacientes.^73^

De acordo com as evidências atuais e seguindo as recomendações das diretrizes brasileiras de 2017, o primeiro passo para a avaliação do paciente com EAo para indicação de intervenção é a definição da gravidade anatômica da valvopatia (Quadro 22). Até o presente momento, apenas pacientes com EAo anatomicamente importante têm benefício de intervenção. A EAo importante é definida ecocardiograficamente com área valvar aórtica (AVA) ≤ 1,0 cm² e/ou AVA indexada ≤ 0,6 cm²/m² na presença de gradiente médio VE/aorta ≥ 40 mmHg ou velocidade máxima do jato aórtico ≥ 4,0 m/s. Pacientes com EAo baixo-fluxo e baixo-gradiente (AVA ≤ 1,0 cm² e gradiente médio VE/aorta < 40 mmHg) quando confirmada a gravidade anatômica, também podem ter indicação de intervenção. No caso daqueles com EAo baixo-fluxo e baixo-gradiente e FEVE preservada, é necessária a realização de escore de cálcio valvar que, se elevado (maior que 1300 AU para mulheres e maior que 2000 AU para homens), confirma EAo importante.^74^^–^^82^ Já naqueles com EAo baixo-fluxo e baixo-gradiente e FEVE reduzida, deve ser realizado ecocardiograma com estresse com dobutamina. Se houver reserva contrátil e a AVA mantiver reduzida, definimos EAo importante.^83^^–^^86^ Se não houver reserva contrátil, também deve ser realizada a avaliação com escore de cálcio valvar para definição da gravidade anatômica.^74^^–^^78^^,^^87^ Tais pacientes sem reserva contrátil também têm benefício com a realização de intervenção cirúrgica ou transcateter.

**Quadro 22:** Passo 1: Diagnóstico de estenose aórtica importante^74^^–^^87^

	Característica de estenose aórtica importante
Exame físico	Pulso *Parvus et Tardus* Sopro sistólico ejetivo com pico telessistólico Hipofonese de B2 Hipofonese de B1 Fenômeno de *Gallavardin* Desdobramento paradoxal de B2 ou B2 única
Eletrocardiograma	Sobrecarga de câmaras esquerdas Alteração de repolarização ventricular (padrão *Strain*)
Radiografia de tórax	Índice cardiotorácico pode ser normal Sinais de congestão pulmonar
Ecocardiograma	AVA ≤ 1,0 cm^2^ AVA indexada ≤ 0,6 cm^2^/m^2^ Gradiente VE/Aorta ≥ 40 mmHg Velocidade máxima do jato aórtico ≥ 4,0 m/s Razão das velocidades de fluxo entre a via de saída do VE e valva aórtica < 0,25
Ecocardiograma sob estresse com dobutamina	Indicado para avaliação de gravidade anatômica em pacientes com EAo de baixo fluxo, baixo gradiente com FEVE reduzida, definida como: AVA ≤ 1,0 cm^2^ com FEVE < 50% e gradiente médio VE/Aorta < 40 mmHg* Na presença de reserva contrátil (aumento ≥ 20% do volume sistólico ejetado e/ou aumento > 10 mmHg no gradiente médio VE/Aorta), pacientes com redução ou manutenção da AVA no pico do estresse possuem EAo importante (se aceita aumento da AVA de até 0,2cm^2^ como critério de manutenção da AVA). Pacientes com aumento da AVA ≥ 0,3 cm² são definidos como portadores de EAo moderada (EAo pseudo-importante) Na ausência da reserva contrátil, devemos corroborar a gravidade anatômica com o escore de cálcio valvar pela tomografia de tórax
Tomografia computadorizada de tórax multidetectora	Escore de cálcio valvar aórtico acima de 1.300 AU para mulheres e 2000 AU para homens reforça possibilidade de valvopatia importante
Estudo hemodinâmico	Gradiente VE/Aorta (pico) ≥ 50 mmHg
Situação especial	EAo de baixo fluxo, baixo gradiente com FEVE preservada (“paradoxal”), definida como: AVA ≤ 1,0 cm^2^ com FEVE > 50% e gradiente médio VE/Aorta < 40 mmHg*. Nestes casos devemos ter os seguintes parâmetros para definição da EAo importante: – AVA indexada ≤ 0,6 cm^2^/m^2^ – Escore de cálcio valvar aórtico elevado – Pressão arterial sistólica ≤ 140 mmHg – Volume ejetado indexado < 35 mL/m^2^ Paciente com todos os parâmetros acima, porém com volume ejetado indexado normal (> 35 ml/m²) são definidos com EAo normofluxo baixo-gradiente. Tal entidade foi recentemente descrita, as evidências são escassas e tais pacientes aparentam ter benefício com a intervenção valvar quando sintomáticos^88^^,^^89^

* Nos casos de EAo com baixo-fluxo, baixo-gradiente e FEVE preservada ou reduzida, devemos atentar para possíveis erros de aferição das medidas ecocardiográficas. AVA: área valvar aórtica; EAo: estenose aórtica; FEVE: fração de ejeção do VE; VE: ventrículo esquerdo.

O segundo passo é a avaliação da etiologia (Quadro 23).^88^^,^^89^ Nos países desenvolvidos, existe maior prevalência de etiologia degenerativa/calcifica nos idosos, enquanto em países subdesenvolvidos, as etiologias reumática e bicúspide predominam em pacientes jovens. No Brasil, devido a sua pirâmide etária transicional, típica de países em desenvolvimento, encontramos um pico bimodal de prevalência de EAo, ou seja, encontramos pacientes de todas as etiologias nas diferentes faixas etárias. A importância da definição da etiologia da EAo também se traduz na escolha do tratamento (Passo 5). Pacientes com EAo reumática usualmente são jovens e não foram contemplados nos estudos de implante transcateter de bioprótese aórtica (TAVI, do inglês *transcatheter aortic valve implantation*). A grande maioria dos pacientes estudados apresentava etiologia degenerativa. Entretanto, já existem evidências da factibilidade do procedimento em pacientes com válvula aórtica bicúspide.^90^

**Quadro 23:** Passo 2: avaliação da etiologia na estenose aórtica importante^88^^,^^89^

	Características etiológicas
Aterosclerótica/degenerativa	Associação com senilidade Prevalência: 3 a 5% da população > 75 anos Relacionada à calcificação valvar aórtica Presença de fatores de risco relacionados à aterosclerose
Reumática	Fusão comissural Acometimento mitroaórtico Faixa etária mais jovem Associada a variados graus de insuficiência aórtica
Bicúspide	Prevalência: 2% da população Associação com aortopatia (70% dos casos) Orientação látero-lateral da fenda comissural: preditor evolutivo de estenose aórtica

O terceiro passo trata-se da avaliação de sintomas atribuíveis à valvopatia (Quadro 24). Pacientes com EAo importante e dispneia, angina ou síncope têm indicação inequívoca de intervenção.

**Quadro 24:** Passo 3: Avaliação de sintomas na estenose aórtica importante

	Sintomas
Dispneia	Disfunção diastólica: hipertrofia ventricular esquerda ➔ redução de complacência ➔ deslocamento da curva pressão/volume ventricular para cima e para a esquerda ➔ elevação das pressões de enchimento ➔ hipertensão venocapilar pulmonar Disfunção sistólica: ocorre na adaptação ventricular inadequada (*afterload mismatch*) e baixo fluxo/baixo gradiente Pacientes com sintomatologia duvidosa (pseudo-assintomático) podem ser submetidos ao teste ergométrico ou ergoespirométrico para avaliação da dispneia ao esforço
Angina	Desbalanço da oferta/consumo de oxigênio no miocárdio hipertrófico Redução do gradiente de perfusão miocárdico (pressão diastólica final elevada)
Síncope	Resulta da incapacidade de incrementos de débito cardíaco em situações de redução expressiva da resistência periférica total Pode decorrer do uso de vasodilatadores (agentes deflagradores comuns) 50% dos casos estão associados a reflexo cardioinibitório

No caso de ausência de sintomas, devemos avaliar a presença de complicadores para indicar intervenção (Quadro 25).^91^^–^^95^ Atualmente, os complicadores contemplados nas diretrizes são:

**Quadro 25:** Passo 4: Avaliação de complicadores na estenose aórtica importante^91^^–^^98^

	Complicadores
Ecocardiograma	Disfunção de ventrículo esquerdo: FEVE < 50% Marcadores de mau prognóstico: AVA < 0,7 cm^2^, velocidade máxima do jato aórtico > 5,0 m/s, gradiente médio VE/Aorta > 60 mmHg
Teste ergométrico/ergoespirométrico	Capacidade funcional limitada Resposta pressórica inadequada: ascensão da pressão artéria sistólica menor do que 20 mmHg ou pressão arterial sistólica com queda maior que 10 mmHg Arritmias: taquicardia ventricular ou mais que 4 extrassístoles ventriculares sucessivas Infradesnivelamento de segmento ST ≥ 2 mm horizontal ou descendente Contraindicado em pacientes sintomáticos e/ou com disfunção ventricular esquerda

AVA: área valvar aórtica; FEVE: fração de ejeção do VE; VE: ventrículo esquerdo.

– Ecocardiograma: disfunção de VE (FEVE < 50%) e/ou marcadores de mau prognóstico (AVA < 0,7 cm^2^, velocidade máxima do jato aórtico > 5,0 m/s, gradiente médio VE/Aorta > 60 mmHg).^96^
– Teste ergométrico: ausência de reserva inotrópica no teste ergométrico e/ou baixa capacidade funcional, hipotensão arterial durante esforço (queda de 20 mmHg na pressão arterial sistólica) e/ou presença de sintomas em baixas carga.^97^^,^^98^

**Figura 5:**
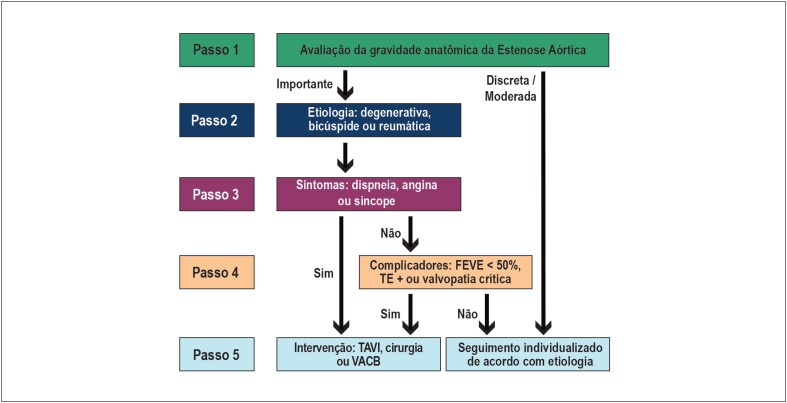
Algoritmo para tomada de decisão na estenose aórtica. FEVE: fração de ejeção do ventrículo esquerdo; TE: teste ergométrico; TAVI: implante de bioprótese aórtica transcateter (da sigla em inglês, transcateter aortic valve implantation); VACB: valvoplastia aórtica cateter-balão.

O quinto e último passo é a escolha da intervenção (Quadros 26, 27 e Figura 5, 6 e 7). A TAVI transfemoral é preferível em relação aos outros acessos torácicos (transaórtico e transapical) por ser menos invasiva e com menor taxa de complicações. Assim, tais acessos são recomendados apenas quando há contraindicação técnica para a realização do acesso femoral.

**Quadro 26:** Passo 5: tipo de intervenção na estenose aórtica importante^90^^,^^99^^–^^132^

Tipo	Considerações
Cirurgia de troca valvar aórtica*	Primeira escolha para pacientes com menos de 70 anos e sem contraindicação ou risco cirúrgico elevado* Pode ser indicada em pacientes com risco intermediário ou idosos com baixo risco a depender da decisão do *Heart Team* e da disponibilidade do procedimento transcateter
Implante de bioprótese aórtica transcateter - TAVI	É necessária avaliação do *Heart Team* institucional Via transfemoral é a preferencial Primeira escolha em pacientes com risco cirúrgico proibitivo, contraindicações à cirurgia convencional, fragilidade ou risco intermediário Ampliada indicação para pacientes de baixo risco cirúrgico (STS < 4%, EuroSCORE II < 4% ou EuroSCORE logístico < 10%) * Acesso transfemoral aparente ser melhor que a cirurgia para esses pacientes Existe uma carência de dados sobre TAVI em pacientes < 70 anos e sobre a durabilidade da prótese Assim, pacientes com baixo risco, idade < 70 anos e sem outras indicações específicas para TAVI, tal procedimento deve ser evitado A angiotomografia de aorta é o exame de escolha para avaliação do acesso a ser utilizado, do tamanho da válvula, tipo de válvula, factibilidade do procedimento e predição de possíveis complicações. Contraindicada para pacientes com expectativa de vida estimada menor que 12 meses
Valvoplastia aórtica por cateter-balão	“Ponte terapêutica” para procedimentos definitivos (cirurgia ou TAVI) em pacientes com instabilidade hemodinâmica ou sintomas avançados Paliação nos casos com contraindicações definitivas à cirurgia convencional e TAVI.

* As diretrizes europeias a norte-americanas atuais são categóricas na indicação preferencial da TAVI em detrimento da cirurgia para pacientes inoperáveis, frágeis e/ou de alto risco cirúrgico (avaliados pelos escores STS e EuroSCORE II). Entretanto, após a publicação de tais diretrizes, 4 trabalhos comparando a TAVI com a cirurgia em pacientes de baixo risco cirúrgico foram publicados. A metanálise de tais estudos demonstrou redução de mortalidade em 1 ano a favor da TAVI transfemoral. Tais resultados sugerem que a TAVI transfemoral deva ser o tratamento preferencial nestes pacientes. Entretanto, um ponto de relevância é a média etária de 75,4 anos e a carência de estudos sobre a durabilidade a longo prazo de tais próteses. Dessa maneira, em pacientes de baixo risco, e estendendo para o risco intermediário, devemos evitar o procedimento em pacientes com menos de 70 anos de idade até que dados robustos de durabilidade das próteses sejam publicados. STS: Society of Thoracic Surgeons; TAVI: implante transcateter de bioprótese aórtica.

**Quadro 27:** Estenose aórtica: Recomendações^1^^,^^2^^,^^90^^,^^99^^–^^132^

Intervenção	Condição clínica	SBC	AHA	ESC
Tratamento cirúrgico convencional ou TAVI*	Sintomas (CF ≥ 2, síncope e angina)	I A	I A	I B
	Assintomático, com complicadores: FEVE < 50% Teste ergométrico +	I B	I B	I C
		IIa B	IIa B	I C
	Assintomático com valvopatia crítica: AVA < 0,7 cm^2^ Velocidade máxima do jato > 5,0 m/s Gradiente médio VE/Aorta > 60 mmHg	IIa C	IIa B	IIa C (BNP elevado para idade; PSAP>60 mmHg; velocidade máxima do jato > 5,5 m/s)
	**Situações especiais**			
	EAo importante de baixo-fluxo/baixo-gradiente com FEVE reduzida: – Com reserva contrátil – Sem reserva contrátil + escore de cálcio elevado EAo importante paradoxal sintomático	IIa B	IIa B	I C
		IIa C	–	IIa C
		IIa C	IIa C	IIa C
Escolha da intervenção entre cirurgia e TAVI**	Inoperável, risco proibitivo e/ou fragilidade – TAVI – Cirurgia	I A	I A	I B
		IIb A	–	–
	Alto risco cirúrgico – TAVI – Cirurgia	I A	I A	I B
		IIa A	I A	–
	Risco cirúrgico intermediário – TAVI – Cirurgia	I A	IIa B	I B
		IIa A	I B	I B
	Baixo risco > 70 anos – TAVI – Cirurgia	I A	–	–
		I A	I B	I B
	Baixo risco < 70 anos – TAVI – Cirurgia	IIb C	–	–
		I A	I B	I B
Valvoplastia aórtica por cateter-balão*	Sintomático com instabilidade hemodinâmica importante, impossibilidade momentânea de intervenção definitiva (TAVI ou cirurgia convencional) — “ponte terapêutica”	IIa C	IIb C	IIb C
	Tratamento paliativo em pacientes sintomáticos e com contraindicações à cirurgia convencional e/ou TAVI.	IIb C	–	–

* Pré-requisito obrigatório: avaliação por Heart Team institucional, contemplando risco cirúrgico, grau de fragilidade, condições anatômicas e comorbidades.

** Outros aspectos como factibilidade técnica, riscos e benefícios de cada procedimento, escolha do paciente, experiência local e disponibilidade dos procedimentos também devem ser levados em consideração para a escolha da técnica. As diretrizes americanas e europeias foram publicadas antes dos trabalhos de TAVI no contexto de baixo risco cirúrgico. Devemos levar tais dados em consideração na comparação das evidências das 3 diretrizes (SBC, AHA e ESC). AHA: American Heart Association; AVA: área valvar aórtica; CF: classe funcional; EAo: estenose aórtica; ESC: European Society of Cardiology; FEVE: fração de ejeção do VE; SBC: Sociedade Brasileira de Cardiologia; TAVI: implante transcateter de bioprótese aórtica; VE: ventrículo esquerdo.

**Figura 6:**
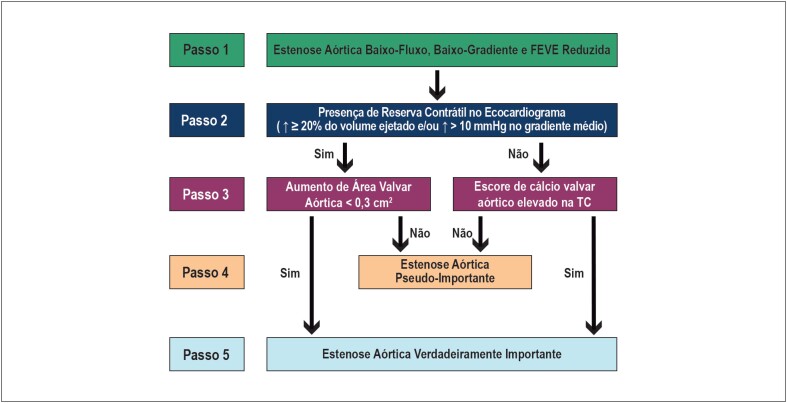
Algoritmo para confirmação da gravidade anatômica na estenose aórtica baixo-fluxo, baixo-gradiente com fração de ejeção reduzida. FEVE: fração de ejeção do ventrículo esquerdo; TC: tomografia computadorizada.

**Figura 7:**
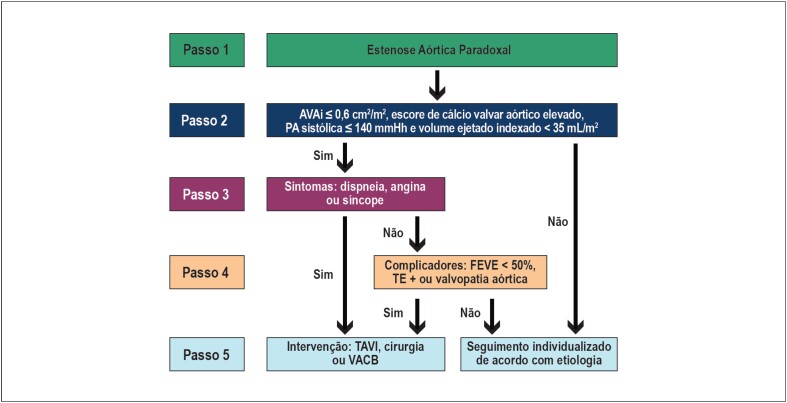
Algoritmo para tomada de decisão na estenose aórtica paradoxal. AVAi: área valvar aórtica indexada; PA: pressão arterial; FEVE: fração de ejeção do ventrículo esquerdo; TE: teste ergométrico; TAVI: implante de bioprótese aórtica transcateter (da sigla em inglês, transcatheter aortic valve implantation); VACB: valvoplastia aórtica cateter-balão.

Todas as diretrizes atuais são categóricas na indicação preferencial da TAVI em detrimento da cirurgia para pacientes inoperáveis, frágeis e/ou de alto risco cirúrgico (avaliados pelos escores STS e EuroSCORE II).^99^^–^^113^ Entretanto, após a publicação de tais diretrizes, 4 trabalhos comparando a TAVI com a cirurgia em pacientes de baixo risco cirúrgico foram publicados. A metanálise de tais estudos demonstrou redução de mortalidade em 1 ano a favor da TAVI transfemoral. Tais resultados sugerem que a TAVI transfemoral deva ser o tratamento preferencial em tais pacientes. Entretanto, um ponto de relevância é a média etária de 75,4 anos. Dessa maneira, em pacientes de baixo risco, e estendendo para o risco intermediário, devemos evitar o procedimento em pacientes com menos de 70 anos de idade até que dados robustos de durabilidade das próteses sejam publicados. ^100^^,^^114^^–^^120^

Outro aspecto relevante e unânime nas diretrizes brasileiras e internacionais é a necessidade de avaliação de cada caso por um *Heart Team*. Outros aspectos como factibilidade técnica, riscos e benefícios de cada procedimento, escolha do paciente, experiência local e disponibilidade dos procedimentos também devem ser levados em consideração para a escolha da técnica.

Alguns grupos de pacientes devem ser monitorizados frequentemente devido o risco de evolução da valvopatia para um quadro que necessite de intervenção (Quadro 28). Tais grupos são:

**Quadro 28:** Estenose aórtica: Acompanhamento individualizado^1^^,^^2^

Estenose aórtica	Acompanhamento	SBC	AHA	ESC
Importante assintomático e sem complicadores	Reavaliação clínica e ecocardiográfica	A cada 6 meses	A cada 0,5-1 ano	A cada 6 meses
	Intervenção concomitante em pacientes que serão submetidos a outro procedimento cirúrgico cardíaco (revascularização coronária, aorta ascendente ou outra válvula)	I C	I B	I C
Moderada (AVA entre 1,0-1,5 cm² e gradiente médio VE/Aorta 25-39 mmHg)	Reavaliação clínica e ecocardiográfica	A cada ano	A cada 1-2 anos	A cada ano
	Intervenção concomitante em pacientes que serão submetidos a outro procedimento cirúrgico cardíaco (revascularização coronária, aorta ascendente ou outra válvula)	IIa C	IIa C	IIa C
Discreta (AVA > 1,5 cm² e gradiente médio VE/Aorta < 25 mmHg)	Reavaliação clínica e ecocardiográfica	A cada 2-3 anos	A cada 3-5 anos	A cada 2-3 anos

AHA: American Heart Association; AVA: área valvar aórtica; ESC: European Society of Cardiology; SBC: Sociedade Brasileira de Cardiologia; VE: ventrículo esquerdo.

– EAo importante assintomático e sem complicadores: até o presente momento, apresentam indicação cirurgia valvar apenas se submetidos a outros procedimentos cardiovasculares invasivos (revascularização coronária, aorta ascendente ou outra válvula). Trabalhos avaliando o benefício de intervenção precoce neste grupo de pacientes estão em andamento.
– EAo moderada, definida como AVA entre 1,0-1,5 cm² e gradiente médio VE/Aorta 25-39 mmHg: apresentam indicação cirurgia valvar apenas se submetidos a outros procedimentos cardiovasculares invasivos (revascularização coronária, aorta ascendente ou outra válvula).
– EAo discreta, definida como AVA > 1,5 cm² e gradiente médio VE/Aorta < 25 mmHg: indicação apenas de acompanhamento clínico/ecocardiográfico.

## 9. Insuficiência Aórtica Crônica

**Figura 8:**
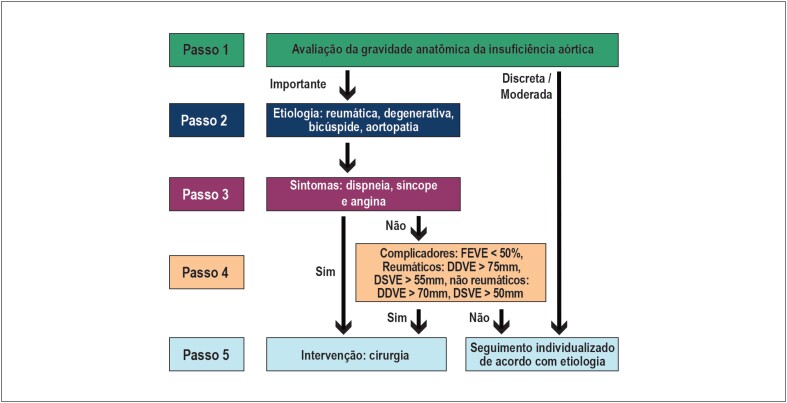
Algoritmo para tomada de decisão na insuficiência aórtica crônica. DDVE: diâmetro diastólico do ventrículo esquerdo; DSVE: diâmetro sistólico do ventrículo esquerdo; FEVE: fração de ejeção do ventrículo esquerdo.

A abordagem clínica escalonada através de cinco passos (Figura 8), marco das Diretrizes Brasileiras de Valvopatias, também é recomendada para o manejo da insuficiência aórtica (IAo) crônica. O primeiro passo para o manejo apropriado dos portadores de IAo consiste na caracterização de sua gravidade anatômica, sobretudo a identificação dos portadores de lesões anatomicamente importantes. O Quadro 29 apresenta os principais achados de exame clínico e de métodos complementares para definição de IAo importante.^133^^,^^134^ De maneira geral, o ecocardiograma transtorácico ainda representa a principal ferramenta para diagnóstico e gradação da gravidade da IAo. A ecocardiografia tridimensional tem sido cada vez mais incorporada na avaliação complementar, especialmente nos casos de limitação na análise bidimensional (jatos excêntricos, determinação anatômica como em valvopatia bicúspide). Além disso, recentemente, destaca-se o surgimento de estudos com a aplicação de ressonância magnética de coração na avaliação da IAo, com possibilidade de aquisição de novos marcadores diagnósticos e prognósticos como a fração regurgitante e estimativa do volume diastólico final do ventrículo esquerdo.^134^

**Quadro 29:** Passo 1: Diagnóstico de insuficiência aórtica importante^133^^,^^134^

	Característica de Insuficiência Aórtica importante
Exame físico	Sopro diastólico aspirativo decrescente com B2 hipofonética Sopro mesossistólico de hiperfluxo Sopro de Austin-Flint (jato da insuficiência aórtica não permite a abertura valvar mitral, gerando sopro diastólico em ruflar) Pulso em martelo d’água ou Corrigan: ascenso rápido e alta amplitude Divergência entre pressão sistólica e diastólica Sinais clínicos de aumento de pressão de pulso: sinal de Musset, sinal de Becker, dança das artérias, sinal de Muller, sinal de Quincke, sinal de Rosenbach, sinal de Gerhard, sinal de Traube, sinal de Duroziez, sinal de Mayne e sinal de Hill
Eletrocardiograma	Sinais de sobrecarga de câmaras esquerdas
Radiografia de tórax	Aumento da silhueta cardíaca à custa de dilatação do VE Sinais de dilatação ou ectasia da aorta
Ecocardiograma	Avaliação da etiologia da doença valvar, diâmetro da aorta ascendente, diâmetros ventriculares, função ventricular. Quantificação da regurgitação: – *Vena contracta* > 0,6 cm – Largura do jato > 0,65 cm – Área do jato ≥ 60% – Fração regurgitante ≥ 50% – Volume regurgitante ≥ 60 mL/batimento – ERO ≥ 0,30cm²
Estudo hemodinâmico	Necessário nos casos de dissociação clínico-ecocardiográfica (elevação da pressão diastólica final do VE, regurgitação aórtica durante a aortografia)
Ressonância Magnética	Avaliação da aorta Avaliação de função ventricular em casos limítrofes Avaliação da função valvar nos casos de dissociação clinicoecocardiográfica Novos preditores: Fração regurgitante e volume diastólico final do ventrículo esquerda
Angiotomografia de aorta	Avaliação da aorta

ERO: área efetiva do orifício regurgitante; VE: ventrículo esquerdo.

No segundo passo (Quadro 30), há que se verificar a etiologia responsável pela IAo. Do ponto de vista etiopatogênico, a IAo crônica relaciona-se a alterações anatômicas relacionadas aos folhetos das semilunares e/ou patologias do ânulo valvar aórtico. Dentre as causas condicionadas por disfunção dos folhetos valvares destacam-se a sequela reumática (ainda uma das principais etiologias no Brasil), endocardite infecciosa (EI), degenerativa, malformações congênitas como a valvopatia bicúspide e degeneração mixomatosa. Quanto às alterações relacionadas ao anel valvar aórtico, destacamos a dissecção de aorta ascendente, dilatações aneurismáticas (provocadas principalmente por hipertensão arterial sistêmica e doenças do colágeno como Marfan e Ehlers-Danlos), espondiloartropatias soronegativas (espondilite anquilosante, doença de Reiter), aortite sifilítica e arterite de Takayasu.^135^^,^^136^

**Quadro 30:** Passo 2: Avaliação da etiologia na insuficiência aórtica importante^135^^,^^136^

	Características etiológicas
Reumática	Alta prevalência no Brasil Geralmente associada à lesão mitral Frequente em adultos jovens
Aterosclerótica	Geralmente associada à EAo Frequente na população idosa
Bicúspide	Associada a alterações da aorta (40% dos casos - aneurisma, dissecção, coarctação) Frequente em adultos jovens
Doenças relacionadas à alteração da geometria da raiz da aorta	Hipertensão arterial sistêmica, dissecção da aorta ascendente, síndrome de Marfan, espondilite anquilosante, aortite sifilítica, osteogênese imperfeita, síndrome de Ehlers-Danlos, síndrome de Reiter, estenose subaórtica e defeito do septo interventricular com prolapso da cúspide aórtica
Outras	Endocardite infecciosa, degeneração mixomatosa, lesões traumáticas, artrite reumatoide

EAo: estenose aórtica.

O terceiro passo (Quadro 31) caracteriza-se pela avaliação de sintomas relacionados à IAo. A identificação dos sintomas pode ser tarefa difícil na rotina assistencial, sobretudo em pacientes idosos que comumente recorrem à prática de autolimitação. Nestes casos, a incorporação de testes funcionais provocativos, como a ergoespirometria, pode auxiliar na identificação destes “assintomáticos” autolimitados. Tendo em vista a elevada morbimortalidade relacionada à instalação de sintomas, sua identificação condiciona o referenciamento do paciente para a intervenção cirúrgica.

**Quadro 31:** Passo 3: Avaliação de sintomas na insuficiência aórtica importante

	Sintomas
Dispneia	Ocorre por aumento da pressão diastólica final secundária à sobrecarga de volume sanguíneo no VE, e consequente congestão venocapilar pulmonar.
Angina	Ocorre pela redução da reserva miocárdica. Pode ocorrer angina noturna pelo aumento da regurgitação valvar decorrente da bradicardia durante o sono
Síncope	Baixo débito cardíaco efetivo

VE: ventrículo esquerdo.

No quarto passo (Quadro 32), destaca-se a avaliação de complicadores relacionados à IAo. Este estágio tem especial relevância nos pacientes assintomáticos. O principal complicador da IAo consiste na perda de função sistólica do VE, relacionada ao estresse sistólico e dilatação ventricular. Chaliki et al, em estudo retrospectivo, verificaram reduzida sobrevida em portadores de IAo com FEVE inferior a 50%. As taxas de mortalidade pós-operatória também foram influenciadas pela função ventricular (14% para pacientes com FEVE inferior a 35%, 6,7% para FEVE entre 35 a 50% e 3,7% para pacientes com FEVE superior a 50%, p = 0,02). ^137^

**Quadro 32:** Passo 4: Avaliação de complicadores^134^^,^^137^^,^ ^137^^–^^139^^,^^141^

	Complicadores
Ecocardiograma	FEVE < 50% DDVE > 70 mm (não reumático) e > 75 mm (reumático) DSVE > 50 mm (não reumático) e > 55 mm (reumático) DSVE indexado > 25 mm/m²
Ressonância Magnética	Presença de fibrose miocárdica (realce tardio) Fração regurgitante > 33% Volume diastólico final do VE > 246 mL
Angiotomografia	Valva Bicúspide com indicação de intervenção + Raiz da Aorta > 45 mm

DDVE: diâmetro diastólico do ventrículo esquerdo; DSVE: diâmetro sistólico do ventrículo esquerdo; FEVE: fração de ejeção do ventrículo esquerdo;; VE: ventrículo esquerdo.

O remodelamento ventricular ainda representa uma ambivalência clínica: de um lado o aumento dos diâmetros ventriculares funciona como um mecanismo adaptativo à sobrecarga volumétrica; por outro lado, o remodelamento ventricular pode determinar pior prognóstico, sobretudo em populações não reumáticas. Em estudo nacional conduzido com 75 pacientes assintomáticos com IAo importante de etiologia reumática, a estratégia de indicar tratamento cirúrgico baseada no surgimento de sintomas mesmo em pacientes com diâmetro diastólico do VE (DDVE) maior que 75mm e DSVE maior que 55mm com função de VE normal foi eficaz em promover a melhora da qualidade de vida e regressão da dilatação, com taxa de sobrevida de 90,6% em dez anos.^138^ Por outro lado, estudos prospectivos com populações com maior predomínio de IAo não reumática verificaram que valores de DSVE acima de 50 mm associaram-se com desfechos clínicos compostos (morte, sintomas e/ou disfunção ventricular) de até 19%/ano. Mais recentemente, há evidências de que a indexação destes diâmetros pela superfície corpórea seja mais apropriada, sobretudo para mulheres. Um estudo com 246 pacientes com IAo assintomáticos verificou que valores de DSVE indexado iguais ou superiores a 25 mm/m^2^ associaram-se a desfechos negativos (mortalidade, sintomas e disfunção ventricular).^139^ Mais recentemente, estudos avaliaram o papel do peptídeo natriurético cerebral (BNP: sigla do inglês *Brain Natriuretic Peptide*) na IAo. Valores de corte de 130 pg/mL para BNP e 602 pg/mL para o NT-pro-BNP associaram-se com desfechos clínicos adversos. A combinação destes valores de BNP com parâmetros ecocardiográficos pode melhorar a capacidade de estratificação dos pacientes assintomáticos. Elevações persistentes de BNP durante o seguimento clínico também foram relacionadas a eventos clínicos adversos.^140^

Parâmetros ecocardiográficos funcionais como o estresse longitudinal também são preditores evolutivos na IAo assintomática, influenciando inclusive os resultados pós-operatórios. A limitação para utilização clínica do estresse longitudinal na IAo consiste na divergência para os pontos de corte a serem utilizados.

Outro complicador relacionado à IAo consiste na fibrose miocárdica pelo realce tardio. A ressonância magnética de coração com realce tardio é o principal método de imagem capaz para sua quantificação. Estudos demonstram que a presença de fibrose miocárdica influencia o período pós-operatório, associando-se à persistência de sintomas, falência de recuperação da função ventricular e maior mortalidade.^141^ Ainda relativo à ressonância magnética, novos estudos demonstram que fração regurgitante acima de 33% e volume diastólico final do VE acima de 246 ml foram associados a menor sobrevida livre de cirurgia. Estes novos parâmetros podem melhorar a estratificação dos pacientes assintomáticos, assegurando indicações cirúrgicas mais precisas.^134^

Por fim, no quinto passo, definimos a necessidade de intervenção na IAo (Quadros 33 e 34). O tratamento cirúrgico com troca valvar aórtica consiste ainda na principal terapia intervencionista na IAo.^142^^,^^143^ As taxas de mortalidade cirúrgica variam de 1% (procedimento de troca valvar isolada) até 7% (procedimentos combinados). O aparecimento de sintomas, redução da função sistólica e remodelamento excessivo do VE geram pior prognóstico e, portanto, são os principais deflagradores do tratamento cirúrgico. Como exposto anteriormente, novos complicadores relacionados à fibrose miocárdica, remodelamento ventricular e comportamento de biomarcadores podem representar potenciais deflagradores de intervenção. O acompanhamento clínico dos pacientes sem indicação de intervenção está descrito no Quadro 35.

**Quadro 33:** Passo 5: Intervenção na insuficiência aórtica importante^142^^,^^143^

Tipo de intervenção	Considerações
Cirurgia (troca valvar)	Tratamento de escolha Troca valvar combinado com correção da aorta ascendente, quando indicada
TAVI	Requer estudos que validem sua indicação

TAVI: implante transcateter de bioprótese aórtica.

**Quadro 34:** Insuficiência aórtica: Recomendações^1^^,^^2^^,^^142^^,^^143^

Intervenção	Condição clínica	SBC	AHA	ESC
Cirurgia de troca valvar	Sintomas	I B	I B	I B
	FEVE < 50%	I B	I B	I B
	Diâmetros ventriculares	IIa B Reumático DDVE > 75 mm ou DSVE > 55 mm IIa B Não reumático DDVE > 70 mm ou DSVE > 50 mm ou DSVE indexado > 25 mm/m²	IIa C DDVE > 70 mm ou DSVE > 50 mm ou DSVE indexado > 25 mm/m²	IIa B DDVE > 70 mm ou DSVE > 50 mm ou DSVE indexado >25 mm/m²
Implante valvar transcateter – TAVI*	Sintomáticos com expectativa de vida > 1 ano com contraindicações/risco proibitivo à cirurgia convencional	IIb C*	–	–

* Considerar discussão junto ao Heart Team. AHA: American Heart Association; DDVE: diâmetro diastólico do ventrículo esquerdo; DSVE: diâmetro sistólico do ventrículo esquerdo; ESC: European Society of Cardiology; FEVE: fração de ejeção do VE; SBC: Sociedade Brasileira de Cardiologia; TAVI: implante transcateter de bioprótese aórtica.

**Quadro 35:** Insuficiência aórtica: Acompanhamento individualizado^1^^,^^2^

Insuficiência aórtica	Acompanhamento	SBC	AHA	ESC
Importante assintomático e sem complicadores	Reavaliação clínica e ecocardiográfica	A cada 0,5 a 1 ano	A cada 0,5 a 1 ano	A cada 3 a 6 meses
	Intervenção concomitante em pacientes que serão submetidos a outro procedimento cirúrgico cardíaco (revascularização coronária, aorta ascendente ou outra válvula)	I C	I C	I C
Moderada (Vena contracta 0,3-0,6 cm, Largura do jato 0,25-0,64, Fração regurgitante 30-49%, Volume regurgitante 30-59mL/batimento, ERO 0,10-0,29 cm²)	Reavaliação clínica e ecocardiográfica	A cada 1-2 anos	A cada 1-2 anos	A cada 1-2 anos
	Intervenção concomitante em pacientes que serão submetidos a outro procedimento cirúrgico cardíaco (revascularização coronária, aorta ascendente ou outra válvula)	IIa C	IIa C	–
Discreta (Vena contracta < 0,3 cm, Largura do jato < 0,25, Fração regurgitante < 30%, Volume regurgitante < 30 ml/batimento, ERO < 0,10 cm²)	Reavaliação clínica e ecocardiográfica	A cada 3-5 anos	A cada 3-5 anos	A cada 1-2 anos

AHA: American Heart Association; ERO: área efetiva do orifício regurgitante; ESC: European Society of Cardiology; SBC: Sociedade Brasileira de Cardiologia.

## 10. Estenose Tricúspide

A estenose tricúspide (ET) é uma valvopatia rara, habitualmente associada à IT. O ecocardiograma mantém-se como principal ferramenta para definição da gravidade anatômica (Quadro 36). ^144^

**Quadro 36:** Passo 1: Diagnóstico de estenose tricúspide importante^144^

	Características de Estenose Tricúspide importante
Exame físico	Estalido de abertura precoce B1 hiperfonética Sopro diastólico em ruflar, com reforço pré-sistólico se paciente em ritmo sinusal em borda esternal esquerda que aumenta com a inspiração. Sinais de congestão sistêmica: hepatomegalia, ascite, edema de membros inferiores, estase jugular, sinal de Kussmaul
Eletrocardiograma	Sobrecarga de AD FA
Radiografia de tórax	Aumento de AD
Ecocardiograma	Área valvar tricúspide ≤ 1,0 cm^2^ Gradiente diastólico médio AD/ventrículo direito ≥ 5 mmHg Aumento isolado de AD PHT tricúspide ≥ 190 ms
Estudo hemodinâmico	Casos de dissociação clinicoecocardiográfica Gradiente diastólico AD/ventrículo direito ≥ 5 mmHg
Ressonância Magnética	Casos de dissociação clinicoecocardiográfica ou qualidade limitada da imagem ecocardiográfica

AD: átrio direito; FA: fibrilação atrial; PHT: pressure half time.

Sua etiologia mais comum é a doença reumática, e neste caso geralmente ocorre concomitantemente ao comprometimento da valva mitral e/ou da valva aórtica. Ocorre espessamento e retração das cúspides, com acometimento comissural. Outras possíveis causas de ET são ainda mais raras, e estão descritas no Quadro 37.^145^^–^^147^

**Quadro 37:** Passo 2: Avaliação da etiologia na estenose tricúspide importante^145^^–^^147^

	Características etiológicas
Reumática	Causa mais prevalente Associação com outras valvopatias Espessamento com retração das cúspides Acometimento comissural Frequente em adultos jovens
Outras	Endocardite infecciosa Lúpus eritematoso sistêmico Síndrome carcinóide Deformidades congênitas Mixoma atrial Lesão actínica (pós-radioterapia) Doença de depósito: amiloidose, doença de Fabry Doença de Whipple

Tanto os sintomas quanto as alterações do exame físico restringem-se habitualmente aos pacientes com ET anatomicamente importante. O sintoma mais comumente encontrado é a fadiga, que pode estar associada a sintomas de insuficiência cardíaca de câmaras direitas (Quadro 38).

**Quadro 38:** Passo 3: Avaliação de sintomas na estenose tricúspide importante

	Sintomas
Fadiga	Principal sintoma Associada a dor e edema de membros inferiores Ausência de dispneia Pode estar associada a palpitações, ascite, sinais de disfunção hepática

Quando os pacientes forem portadores de ET importante, mas ainda não tiverem sintomas secundários à valvopatia, deve ser avaliado se apresentam ou não complicadores (Quadro 39).

**Quadro 39:** Passo 4: Avaliação de complicadores na estenose tricúspide importante

	Complicadores
Eletrocardiograma	FA
Congestão sistêmica	Avaliação de comprometimento hepático (elevação de enzimas, alteração do coagulograma)

FA: fibrilação atrial.

**Figura 9:**
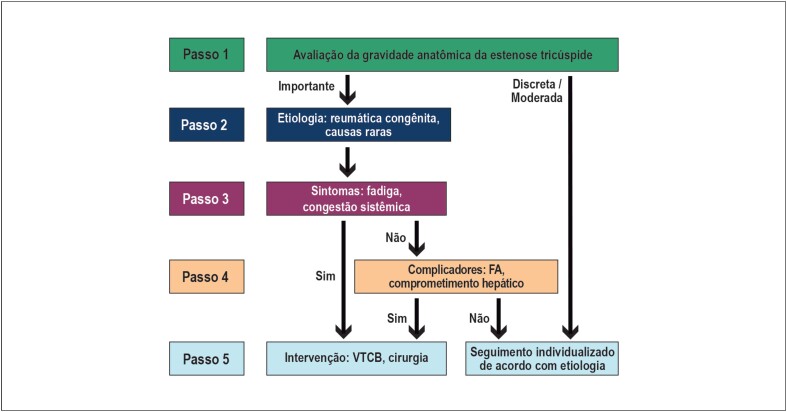
Algoritmo para tomada de decisão na estenose tricúspide. FA: fibrilação atrial; VTCB: valvoplastia tricúspide por cateter-balão.

A presença de sintomas ou dos complicadores descritos acarreta na indicação de intervenção sobre a valvopatia. Apesar da raridade dos casos e da escassez da literatura, a valvoplastia tricúspide por cateter-balão (VCTB) ainda é o tratamento de escolha (Quadros 40, 41 e Figura 9).^148^

**Quadro 40:** Passo 5: Tipo de intervenção na estenose tricúspide importante^148^

Tipo	Considerações
Valvoplastia tricúspide por balão	Tratamento de escolha Possível fazer em pacientes com refluxo tricuspídeo moderado. Contraindicado se presença de trombo refratário à anticoagulação e/ou vegetação
Troca da valva tricúspide	Opção em caso de impossibilidade de valvoplastia por balão. Preferência por prótese biológica Preferível se associada à cirurgia para tratamento da valvopatia mitral.

**Quadro 41:** Estenose tricúspide: Recomendações^1^^,^^2^^,^^148^

Intervenção	Condição clínica	SBC	AHA	ESC
Valvoplastia tricúspide por cateter-balão	ET importante isolada sintomática sem contraindicações	IIa C	IIb C	–
	VMCB concomitante	I C	I C	–
	VTCB com IT importante	III	–	–
Troca da valva tricúspide ou plástica (comissurotomia)	ET importante sintomática com contraindicação a VTCB	I C	I C	I C
	ET importante isolada sintomática	IIa C	I C	I C
	Preferência por prótese biológica em caso de troca valvar	I C	–	–

AHA: American Heart Association; ESC: European Society of Cardiology; ET: estenose tricúspide; SBC: Sociedade Brasileira de Cardiologia; VTCB: valvoplastia tricúspide por cateter-balão.

## 11. Insuficiência Tricúspide

Pacientes com insuficiência tricúspide (IT) discreta habitualmente não requerem qualquer tipo de abordagem direcionada à valva tricúspide. Os pacientes com IT moderada a importante são aqueles que exigirão um acompanhamento específico, em particular relacionado ao esclarecimento da etiologia da valvopatia e das repercussões a ela associadas (Quadro 42).^149^

**Quadro 42:** Passo 1: Diagnóstico de insuficiência tricúspide importante^149^

	Características de Insuficiência Tricúspide Importante
Exame físico	Turgência jugular patológica B2 hiperfonética (hipertensão arterial pulmonar) Sopro sistólico regurgitativo em borda esternal esquerda com sinal de Rivero-Carvallo Hepatomegalia
Eletrocardiograma	Sobrecarga de câmaras direitas FA
Radiografia de tórax	Sinais de aumento de câmaras direitas Congestão pulmonar se lesão concomitante do lado esquerdo Retificação/abaulamento de tronco pulmonar
Ecocardiograma	ERO ≥ 0,40cm² Fluxo reverso nas veias hepáticas Volume regurgitante > 45 ml/batimento Volume regurgitante denso, triangular e pico precoce no Doppler contínuo. *Vena contracta* ≥ 0,7 cm Diâmetro do anel ≥ 40mm Falha de coaptação das cúspides
Estudo hemodinâmico	Em caso de dissociação clinicoecocardiográfica Mensurar PSAP em casos de falha de coaptação das cúspides
Ressonância Magnética	Em caso de dissociação clinicoecocardiográfica ou qualidade limitada da imagem ecocardiográfica

FA: fibrilação atrial; ERO: área efetiva do orifício regurgitante; PSAP: pressão sistólica da artéria pulmonar.

A IT é habitualmente funcional, secundária a condições clínicas que levam à dilatação do anel valvar tricúspide, principalmente envolvendo as câmaras cardíacas esquerdas (doenças valvares ou cardiomiopatias) e/ou HP. Os casos de IT primária em geral estão relacionados à doença reumática, podendo ainda estar associados a intervenções médicas (biópsias endomiocárdicas de repetição, presença de eletrodos de marcapasso ou cardiodesfibrilador implantável) ou ocorrer em consequência de EI ou outras causas mais raras (Quadro 43).^150^

**Quadro 43:** Passo 2: Avaliação da etiologia na insuficiência tricúspide importante^150^

	Características etiológicas
Primária	Acometimento reumático Prolapso e degeneração mixomatosa Lesão actínica por radiação (pós-radioterapia) Trauma torácico fechado Endocardite Infecciosa Biopsia endomiocárdica de repetição Síndrome carcinoide Congênita (Ebstein) Eletrodos de marcapasso ou desfibrilador
Secundária	Dilatação do ânulo tricúspide (> 40 mm ou > 21 mm/m²) Doença valvar do lado esquerdo do coração FA de longa duração HP primária Miocardiopatia de ventrículo direito (isquêmica, displasia arritmogênica, miocárdio não compactado, cardiomiopatia hipertrófica) Pericardite constrictiva
Causas raras	Doenças reumatológicas Drogas (metissergida/anorexígenos) Doença de Fabry

FA: fibrilação atrial; HP: hipertensão pulmonar.

Nos pacientes com IT importante, conforme aumenta o período de tempo em que o paciente mantém disfunção valvar significativa, poderão surgir sintomas que terão impacto significativo para a tomada de decisão sobre o melhor tratamento a ser instituído (Quadro 44).

**Quadro 44:** Passo 3: Avaliação de sintomas na insuficiência tricúspide importante

Sintomas	
Dispneia (NYHA II – IV)	Na IT secundária decorre da doença do lado esquerdo do coração (congestão venocapilar pulmonar, hipertensão arterial pulmonar). Dispneia aos esforços e paroxística noturna
Fadiga	Principal sintoma Associada a dor e edema de membros inferiores Mais comum na insuficiência cardíaca direita

IT: insuficiência tricúspide; NYHA: New York Heart Association.

Por outro lado, mesmo naqueles pacientes que não apresentarem sintomas pode haver evolução com remodelamento do ventrículo direito, que poderá justificar intervenção sobre a valva. Assim, os pacientes com dilatação ou disfunção (exceto importante) do ventrículo direito serão considerados como portadores de fator complicador (Quadro 45).

**Quadro 45:** Passo 4: Avaliação de complicadores na insuficiência tricúspide importante

Complicadores	
Ecocardiograma	IT primária: dilatação ou disfunção progressiva de ventrículo direito

IT: insuficiência tricúspide.

Novos dados têm elucidado a importância prognóstica da IT. Um estudo recentemente publicado encontrou uma prevalência de IT moderada a importante de 0,55% na população, sendo 72% dos casos secundários à presença de valvopatia esquerda (49,5%) ou HP (23%). Neste estudo, apenas 8% dos casos ocorreram de forma isolada. Os pacientes com IT isolada moderada a importante apresentaram maior taxa de mortalidade (risco relativo 1,68, com IC 95% 1,04 a 2,6, p = 0,03), confirmando dados que haviam sido publicados pelo mesmo grupo já em 2014.^151^ Esta maior mortalidade foi também demonstrada em uma metanálise recente, na qual foram incluídos 70 estudos, tendo sido encontrado quase o dobro de mortalidade nos pacientes portadores de IT moderada ou importante (risco relativo 1,95, IC 95% 1,75 a 2,17). Esta maior taxa de óbito foi mantida nas análises mesmo após serem feitos ajustes pela presença ou não de disfunção do ventrículo direito, de HP, de FA, de IM ou de disfunção de VE.^152^

O tratamento intervencionista de escolha, quando indicado, será a plástica da valva tricúspide, com utilização de anel protético capaz de diminuir o diâmetro do anel tricuspídeo, melhorar a coaptação dos folhetos valvares e corrigir a regurgitação. A troca valvar fica reservada aos pacientes sem condição anatômica para que seja realizada plástica com resultado satisfatório. Cabe observar que a abordagem cirúrgica isolada sobre a valva tricúspide segue sendo pouco indicada atualmente, e apresenta o maior risco cirúrgico entre as cirurgias valvares, com taxas de mortalidade cirúrgica que variam de 8,8% a 9,7%. Além disso, apesar dos estudos que demonstram aumento da taxa de mortalidade em pacientes com IT moderada a importante, ainda não há dados demonstrando melhora de sobrevida com o tratamento cirúrgico. Assim, a indicação cirúrgica ainda tem como principal objetivo, nesta população, a melhora dos sintomas e a prevenção de disfunção importante do ventrículo direito.^153^^–^^155^

**Figura 10:**
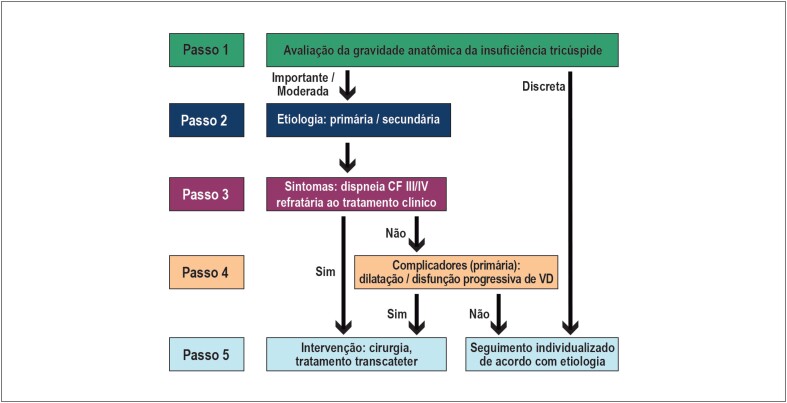
Algoritmo para tomada de decisão na insuficiência tricúspide. VD: ventrículo direito.

Paralelamente à cirurgia, tem crescido o número de estudos voltados ao tratamento intervencionista percutâneo da IT. Diversos dispositivos foram desenvolvidos, com estratégias que se baseiam na diminuição do anel valvar tricuspídeo, ou na melhora da coaptação entre os folhetos, ou ainda nos implantes valvares transcateter. Novos dados estarão disponíveis no futuro, e poderão dar maior suporte ao *Heart Team* para a indicação do implante destes dispositivos em pacientes com IT (Quadros 46, 47 e Figura 10) ^149^^,^^156^^–^^158^

**Quadro 46:** Passo 5: Tipo de intervenção na insuficiência tricúspide importante^149^^,^^151^^–^^158^

Tipo	Considerações
Plástica tricúspide com anel protético	Tratamento de escolha
	Indicações: – Abordagem cirúrgica de outra valvopatia na presença de: anel tricuspídeo ≥ 40 mm e/ou IT moderada a importante – IT isolada, refratária ao tratamento clínico, sem contraindicações e de baixo risco cirúrgico.
	Contraindicações: disfunção sistólica do ventrículo direito importante
Troca valvar cirúrgica	Quando plástica contraindicada Preferência por prótese biológica.
Implante valvar transcateter	Sintomas refratários ao tratamento clínico, com contraindicação ou alto risco a tratamento cirúrgico (em estudo)

IT: insuficiência tricúspide.

**Quadro 47:** Insuficiência tricúspide: Recomendações^1^^,^^2^^,^^149^^,^^151^^–^^158^

Intervenção	Condição clínica	SBC	AHA	ESC
Plástica tricúspide com anel protético	Abordagem de outra valvopatia e IT importante	I C	I C	I C
	Abordagem de outra valvopatia e anel tricúspide ≥ 40 mm	IIa C	IIa B	IIa C
	Abordagem de outra valvopatia, IT importante e sinais de disfunção de ventrículo direito	IIa C	IIa B	IIa C
	Abordagem de outra valvopatia, IT moderada a importante e/ou anel ≥ 40 mm com PSAP ≥ 70 mmHg	IIa C	IIb C	IIa C
	IT importante isolada refratária ao tratamento clínico	IIa C	IIa C	IIa C
	IT importante primária assintomática isolada com dilatação ou perda de função progressiva de ventrículo direito	IIb C	IIb C	IIa C
Troca valvar cirúrgica	IT com indicação de abordagem sem possibilidade de plástica	I C	I C	I C
	Preferência para prótese biológica	I B	–	–
Implante valvar tricúspide transcateter	Refratária ao tratamento clínico, com contraindicação ou alto risco a tratamento cirúrgico (em estudo)	IIb C*	–	–

* Considerar discussão junto ao Heart Team. AHA: American Heart Association; ESC: European Society of Cardiology; IT: insuficiência tricúspide; SBC: Sociedade Brasileira de Cardiologia.

## 12. Disfunção de Prótese Valvar

Após cirurgia de troca valvar, os seguimentos clínico e ecocardiográfico periódicos devem ser realizados para a detecção precoce de disfunção de prótese, assim como para a identificação de sintomas e complicadores anatômicos e funcionais secundários a essa disfunção.

O principal exame para suspeita e diagnóstico da disfunção de prótese é o ecocardiograma transtorácico. Entretanto, ecocardiograma transesofágico e a angiotomografia de aorta com sincronizada ao ECG ganham espaço principalmente na avaliação de trombose de bioprótese (Quadros 48 e 49).^159^

**Quadro 48:** Passo 1: Diagnóstico de disfunção de prótese valvar importante

	Características da Disfunção de Prótese Valvar Importante
Exame físico	Sinais clínicos conforme o tipo da disfunção de prótese predominante
Eletrocardiograma	Alterações condizentes com o tipo de disfunção de prótese predominante
Radiografia de tórax	Alterações condizentes com a disfunção de prótese predominante
Ecocardiograma transtorácico	Avaliação do tipo de disfunção valvar e confirmar gravidade da disfunção – espessamento de folhetos – calcificação e mobilidade de folhetos – redução da AOE – gradiente transvalvar – regurgitação valvar Avaliar disfunção ventricular sistólica Avaliação evolutiva dos diâmetros das câmaras cardíacas
Ecocardiograma transesofágico	Janela inadequada para ecocardiograma transtorácico Regurgitação paravalvar importante com anatomia favorável para tratamento percutâneo Indicado quando necessária melhor avaliação anatômica
Estudo hemodinâmico com manometria	Necessário nos casos de dissociação clinicoecocardiográfica
Angiotomografia de aorta sincronizada ao ECG	Avaliação da aorta Avaliação de trombose de bioprótese e TAVI

AOE: área efetiva do orifício; ECG: eletrocardiograma; TAVI: implante transcateter de bioprótese aórtica.

**Quadro 49:** Passo 2: Avaliação da etiologia da disfunção de prótese valvar importante^159^

	Características etiológicas
Estenose de prótese	**Prótese mecânica:** trombose *pannus* **Prótese biológica:** degeneração de folhetos calcificação de folhetos *mismatch* prótese-paciente (AOE indexada ≤ 0,85 cm^2^/m^2^)
Regurgitação de prótese	**Central:** degeneração de folheto (ruptura, perfuração) calcificação de folheto **Paravalvar:** endocardite infecciosa degeneração do anel

AOE: área efetiva do orifício.

O sintoma mais frequentemente encontrado é a dispneia, decorrente da congestão venocapilar pulmonar consequente a disfunção valvar (Quadro 50).

**Quadro 50:** Passo 3: Avaliação de sintomas da disfunção de prótese valvar importante

Sintomas	
Dispneia (NYHA II – IV)	Fisiopatologia conforme disfunção predominante, determinando congestão venocapilar pulmonar

NYHA: New York Heart Association.

A avaliação de complicadores na disfunção de prótese é complexa pois em muitos casos o paciente já apresenta HP, dilatação ou disfunção ventricular decorrente da valvopatia prévia. Dessa maneira, a progressão de tais alterações deve ser notada e levada em consideração para indicação de intervenção (Quadro 51).

**Quadro 51:** Passo 4: Avaliação de complicadores da disfunção de prótese valvar importante

	Complicadores
Ecocardiograma	Progressão de disfunção ventricular sistólica Progressão de remodelamento de VE (caso diâmetros iniciais já elevados) HP Calcificação importante de bioprótese
Anemia hemolítica	Ocorre em casos de regurgitação importante de prótese valvar, sobretudo se paravalvar

HP: hipertensão pulmonar; VE: ventrículo esquerdo.

Novos procedimentos, como tratamento percutâneo de regurgitação paravalvar e *valve-in-valve* já são realidade e estão incluídos nas novas Diretrizes (Quadros 52 e 53).^159^^–^^162^

**Quadro 52:** Passo 5: Tipo de intervenção da disfunção de prótese valvar importante ^159^^–^^162^

Tipo	Considerações
Cirurgia (retroca valvar)	Tratamento de escolha Indicações: disfunção importante de prótese valvar, com sintomas e/ou anemia hemolítica grave
Intervenção por cateter - *Valve-in-valve*	Disfunção de bioprótese mitral ou aórtica, em pacientes sintomáticos e com alto risco para cirurgia ou inoperáveis (após avaliação do *Heart Team*)
Oclusão percutânea de regurgitação paravalvar	Em casos de regurgitação paravalvar importante, associada à anemia hemolítica ou sintomas de insuficiência cardíaca (CF III/IV), em pacientes com alto risco para cirurgia e com anatomia favorável ao procedimento, em centro capacitado

CF: classe funcional.

**Quadro 53:** Disfunção de prótese valvar: Recomendações^1^^,^^2^^,^^159^^–^^162^

Intervenção	Condição clínica	SBC	AHA	ESC
Retroca valvar cirúrgica	Disfunção importante de prótese valvar, com sintomas	I B	I B	I C
	Anemia hemolítica	IB	I B	I C
	Disfunção importante de prótese valvar, assintomático, com baixo risco para cirurgia	IIa C	IIa C*	IIa C
Oclusão percutânea de regurgitação paravalvar	Hemólise ou sintomas, com anatomia favorável e alto risco para cirurgia, após avaliação do *Heart Team*.	IIa B	IIa B	–
Valve-in-valve	Disfunção importante de bioprótese, em pacientes sintomáticos, com alto risco para cirurgia ou inoperáveis, após avaliação do *Heart Team*.	IIa B	IIa B	IIa C

* Bioprótese aórtica com regurgitação. AHA: American Heart Association; ESC: European Society of Cardiology; SBC: Sociedade Brasileira de Cardiologia.

## 13. Doença Multivalvar

Considera-se doença multivalvar o acometimento primário de duas ou mais valvas. Portanto, são excluídas dessa classificação valvopatias secundárias a uma valvopatia primária, como é o caso da IT funcional, consequente à doença mitral, assim como a IM secundária ao remodelamento ventricular esquerdo consequente à valvopatia aórtica (Quadro 54).^163^^–^^165^

**Quadro 54:** Passo 1: Diagnóstico de doença multivalvar importante^163^^–^^165^

	Características de Doença Multivalvar Importante
Exame físico	Presença de sopros distintamente caracterizados como mitral e aórtico - insuficiência, estenose ou dupla lesão. Excluir possibilidade de sopro causado por interferência hemodinâmica (ex.: sopro de Austin-Flint) Excluir possibilidade de valvopatias secundárias a uma valvopatia primária (ex.: IT secundária à doença mitral). Exame físico especialmente importante para definir a predominância de uma das valvopatias
Eletrocardiograma	Sobrecarga de câmaras esquerdas, dependendo da valvopatia predominante FA em valvopatias mitrais importantes
Radiografia de tórax	Índice cardiotorácico aumentado, especialmente em associação de valvopatias regurgitantes Sinais de congestão pulmonar Sinais de sobrecarga do ventrículo direito em lesão mitral estenótica associada
Ecocardiograma	Os achados ecocardiográficos variam de acordo com as valvopatias
Estudo hemodinâmico	Indicado na dissociação clinicoecocardiográfica

FA: fibrilação atrial; IT: insuficiência tricúspide; VE: ventrículo esquerdo.

No Brasil, a doença multivalvar é resultado do acometimento reumático na maioria das vezes, porém há um aumento progressivo de doença mitroaórtica degenerativa calcífica (Quadro 55).^159^

**Quadro 55:** Passo 2: Avaliação da etiologia da doença multivalvar importante^159^^,^^163^^–^^165^

	Características etiológicas
Febre reumática	> 95% dos casos Típico de pacientes jovens Frequente evolução extemporânea. Sintomas entre os 20 e 40 anos Fusão comissural, espessamento de cúspides, frequente dupla disfunção - fisiopatologia complexa Comprometimento do aparelho subvalvar
Endocardite Infecciosa	Insuficiência valvar por destruição do aparelho mitral e/ou aórtico Infecção metastática aorticomitral
Calcificação do aparelho valvar	Pacientes idosos ou muito idosos Associação com valvopatia aórtica aterosclerótica Calcificação do anel valvar mitral com calcificação caseosa Ausência de fusão comissural Relação com calcificação aórtica e coronariana
Síndrome de Marfan/Ehlers-Danlos	Insuficiências valvares mitral e aórtica Pesquisar acometimento de aorta ascendente

Os sintomas geralmente estão associados à valvopatia de maior gravidade anatômica e, nos casos em que ambas são importantes, a valvopatia mais proximal costuma prevalecer (Quadro 56).

**Quadro 56:** Passo 3: Avaliação de sintomas da doença multivalvar importante

	Sintomas
Dispneia (NYHA II – IV)	Principal sintoma Inicialmente com eventos que aumentam a pressão venocapilar pulmonar Pode ser acompanhada por palpitações, hemoptise, disfonia, disfagia, tosse Insuficiência cardíaca direita associada em portadores de hipertensão pulmonar
Dor precordial	Especialmente com associação de valvopatia aórtica regurgitante ou estenótica Pode ser causada por HP
Baixo débito/síncope	Presente especialmente na associação EAo + IM

EAo: estenose aórtica; HP: hipertensão pulmonar; IM: insuficiência mitral.

Os complicadores, quando presentes, decorrem da valvopatia com maior gravidade anatômica (Quadro 57).

**Quadro 57:** Passo 4: Avaliação de complicadores da doença multivalvar importante

	Complicadores
Hipertensão pulmonar	PSAP ≥ 50 mmHg em repouso Mais presente quando há estenose mitral associada Sintomas de insuficiência cardíaca direita Relação com aumento do risco cirúrgico
Fibrilação atrial de início recente	Relação com remodelamento do AE
Aumento de diâmetros ventriculares	Considerar diâmetros a depender do tipo de lesão valvar

AE: átrio esquerdo; PSAP: pressão sistólica da artéria pulmonar.

O tratamento padrão da doença mitroaórtica com sintomas e/ou complicadores é o tratamento cirúrgico, no entanto, as estratégias transcateter podem ser indicadas em casos selecionados, principalmente em pacientes com alto risco presumido para cirurgia convencional (Quadros 58 e 59).^163^^–^^165^

**Quadro 58:** Passo 5: Tipo de intervenção da doença multivalvar importante^163^^–^^165^

Tipo	Considerações
Valvoplastia mitral por cateter-balão	Casos de EM importante com anatomia favorável e valvopatia aórtica moderada
Tratamento cirúrgico (comissurotomia/troca valvar)	Cirurgia conservadora da valva mitral quando há predomínio de estenose Evitar plástica valvar aórtica - frequente recorrência da valvopatia e sintomas, mesmo com bom resultado imediato Abordagem da valvopatia anatomicamente moderada concomitante a intervenção da valvopatia importante
Tratamento transcateter – *Valve-in-Valve*	Disfunção de bioprótese mitral e aórtica, em pacientes sintomáticos e com alto risco para cirurgia ou inoperáveis (após avaliação do *Heart Team*)
Tratamento transcateter – TAVI e clipagem percutânea mitral	EAo importante e IM primária importante, em pacientes sintomáticos ou com complicadores, com alto risco para cirurgia ou inoperáveis (após avaliação do *Heart Team*)

EAo: estenose aórtica; EM: estenose mitral; IM: insuficiência mitral; TAVI: implante transcateter de bioprótese aórtica.

**Quadro 59:** Doença multivalvar: Recomendações^1^^,^^2^^,^^163^^–^^165^

Intervenção	Condição clínica	SBC	AHA	ESC
Valvoplastia mitral por cateter-balão	EM: estenose mitral importante sintomática com anatomia favorável e lesão aórtica moderada	I A	–	–
Tratamento cirúrgico/troca valvar	Doença multivalvar sintomática	I B	I B	I B
	Doença multivalvar com complicadores	IIa C	–	–
	Abordagem de lesão valvar moderada concomitante ao tratamento de valvopatia importante ou outra cirurgia cardíaca ou de aorta ascendente	I C	I C	I C
Tratamento transcateter – Valve-in-Valve	Disfunção de prótese biológica mitral e aórtica com sintomas e alto risco cirúrgico	IIb C	–	–
	Disfunção de prótese biológica mitral e aórtica com complicadores e alto risco cirúrgico	IIb C	–	–
Tratamento transcateter – TAVI e clipagem percutânea mitral^®^	EAo importante e IM primária importante com sintomas e alto risco cirúrgico	IIb C	–	–
	EAo importante e IM primária importante com complicadores e alto risco cirúrgico	IIb C	–	–

AHA: American Heart Association; EAo: estenose aórtica; ESC: European Society of Cardiology; IM: insuficiência mitral; SBC: Sociedade Brasileira de Cardiologia; TAVI: implante transcateter de bioprótese aórtica.

## 14. Avaliação da Doença Arterial Coronariana

Pacientes com indicação de cirurgia valvar devem ser submetidos à avaliação de doença arterial coronariana com cineangiocoronariografia se: idade maior que 40 anos, suspeita de doença arterial coronariana (fatores de risco para aterosclerose [diabetes, dislipidemia, hipertensão arterial, dentre outros], eventos prévios, angina), disfunção ventricular esquerda ou para avaliação de etiologia na IM secundária.^166^^–^^168^ A avaliação poderá ser com angiotomografia de coronária nos casos de pacientes com baixa ou intermediária probabilidade de doença arterial coronária. Se a angiotomografia demonstrar lesões significativas ou duvidosas, o paciente deverá ser submetido à cineangiocoronariografia (Quadro 60).^169^^–^^171^

**Quadro 60:** Intervenção na doença arterial coronária concomitante à intervenção Valvar: Recomendações^1^^,^^2^^,^^166^^–^^171^

Intervenção	Condição clínica	SBC	AHA	ESC
Revascularização miocárdica cirúrgica	Indicação de intervenção valvar cirúrgica e lesão coronária ≥ 70%	I C	IIa C	I C
Angioplastia coronária	Indicação de intervenção valvar transcateter e lesão coronária ≥ 70% em segmento proximal	IIa C	IIa C	IIa C

AHA: American Heart Association; ESC: European Society of Cardiology; IM: insuficiência mitral; SBC: Sociedade Brasileira de Cardiologia.

## 15. Anticoagulação

Os dois complicadores de maior impacto na história natural da doença valvar são as alterações hemodinâmicas e o tromboembolismo. O acidente vascular cerebral é o evento tromboembólico de maior significância clínica, acometendo até 20% dos indivíduos com FA associada à doença valvar. Está recomendada a aplicação do escore CHA_2_DS_2_-VASc para decisão quanto à anticoagulação, exceto nos pacientes portadores de EM reumática e naqueles com prótese mecânica. Os critérios para anticoagulação são os mesmos em portadores de FA paroxística, persistente ou permanente. As principais indicações de anticoagulação estão descritas no Quadro 61.

**Quadro 61:** Indicações de anticoagulação oral^1^^,^^2^^,^ ^172^^–^^183^

Condição clínica	Medicação	SBC	AHA	ESC
**Valva nativa**				
EM com FA e/ou trombo atrial esquerdo*	Varfarina	I B	I B	I B
	DOACs	III C	III C	III C
	AAS	IIb B	–	–
Demais valvopatias com FA	Varfarina	I B	I C	I B
	DOACs	IIa C	IIa C	IIa B
	AAS	IIb B	–	–
Evento embólico prévio sem FA	Varfarina	I B	I B	–
	DOACs	III C	–	–
	AAS	IIb C	–	–
**Prótese biológica**				
FA	Varfarina	I B	I B	I C
	DOACs	IIb B	–	–
	AAS	IIb C	–	–
Ritmo sinusal – prótese mitral (primeiros 3-6 meses)	Varfarina	IIb	IIa B	IIa C
	DOACs	III C	–	–
	AAS	IIb	–	–
Ritmo sinusal – prótese aórtica (primeiros 3-6 meses)	Varfarina	IIb B	IIa B	IIb C
	DOACs	III C	–	–
	AAS	IIb B	–	IIa C
**TAVI**				
FA	Varfarina	I B	–	–
	DOACs	IIb C	–	–
	AAS + clopidogrel	III B	–	–
	AAS	III C	–	–
Ritmo sinusal	Varfarina	III B	IIb B 3 meses	IIb C 3 meses
	DOACs	III B	–	–
	AAS ou clopidogrel indefinidamente	IIa B	–	IIb C
	AAS + clopidogrel 3-6 meses	IIb B	IIb C	IIa C
Ritmo sinusal + angioplastia com stent (doença arterial coronária crônica)	AAS + clopidogrel até 12 meses conforme tipo de stent	IIa C	IIb	–
FA + angioplastia com stent (doença arterial coronária crônica)	DOAC + clopidogrel	IIa C	–	–
	Varfarina + AAS + clopidogrel 1m, seguido de varfarina + clopidogrel até 12m	IIb C	–	–
**Prótese mecânica**				
	Varfarina	I B	I A	I B
	DOACs	III B	III B	III B
	Varfarina + AAS rotineiramente	III C	IIa B	–
	Varfarina + AAS após evento tromboembólico com INR terapêutico	IIa B	–	IIa C

* Considerar anticoagulação com varfarina nos indivíduos com EM com episódios de taquicardia atrial sustentada ou aumento de AE (≥ 50 mm de diâmetro anteroposterior ou ≥ 50 ml/m² de volume de AE) e contraste espontâneo. AAS: ácido acetilsalicílico; AHA: American Heart Association; DOACs: anticoagulantes orais diretos; ESC: European Society of Cardiology; EM: estenose mitral; FA: fibrilação atrial; IM: insuficiência mitral; INR: razão normalizada internacional; SBC: Sociedade Brasileira de Cardiologia; TAVI: implante transcateter de bioprótese aórtica.

Atualmente a anticoagulação oral como forma de prevenir eventos tromboembólicos nos portadores de doença valvar ainda é feita predominantemente com antagonistas da vitamina K (sigla em inglês VKA – *vitamin K antagonist*), sendo a varfarina o atual representante desta classe no Brasil. É uma estratégia segura iniciar a varfarina na dose de 5mg/dia para indivíduos abaixo dos 65 anos e 2,5 mg/dia acima dos 65 anos. O tempo de protrombina deverá ser dosado no 3º dia para avaliação de hiper-responsividade à medicação e novamente no 5º dia, data a partir da qual a dose passa a ser ajustada. Nesta fase os exames devem ser feitas com intervalo de até 5 dias, até que se atinja nível terapêutico. A razão normalizada internacional (INR) deverá ficar entre 2,0 e 3,0, exceto para os portadores de prótese mecânica em posição mitral, prótese mecânica aórtica associada à FA, estados de hipercoagulabilidade e eventos cardioembólicos na vigência de INR entre 2,0 e 3,0. Nestes casos, o alvo passa a ser 2,5 a 3,5. O controle da INR habitualmente é realizado mensalmente, sendo razoável o controle a cada dois meses em pacientes com doses estáveis de longa data e que não foram expostos a novos fatores que interajam com a varfarina (Quadro 62). No caso de INR fora do alvo, deve ser coletado novo exame mais precocemente, em 1 a 2 semanas. O ajuste de dose deve ser em média 10-15% da dose semanal e devem ser investigados os fatores que ocasionaram a oscilação da INR. O monitoramento do tempo de protrombina com dispositivos *point-of-care* fornece informações rápidas e confiáveis, porém sua disponibilidade ainda é limitada pelo alto custo do aparelho e tiras.

**Quadro 62:** Ajuste de dose da varfarina

Valor de INR	Ajuste de dose
≤ 1,5	Aumentar 15% na dose semanal
1,51 – 1,99	Aumentar 10% na dose semanal
2 – 3*	Manter dose
3,01 – 4,0	Reduzir 15% na dose semanal
4,01 – 4,99	Suspender 1 dose e reduzir 10% na dose semanal
5,0 – 8,99	Suspender a varfarina até INR 2-3 e reiniciar com 15% menos da dose semanal
≥ 9,00	Internação hospitalar, suspender a varfarina em média por 4 dias, prescrever vitamina K na dose de 1 a 2,5 mg por via oral a ser repetida em 24-48 horas se não houver redução para INR <5,0 e reiniciar a anticoagulação após INR próximo aos valores-alvo (abaixo de 4)

* Considerar manutenção da dose semanal da varfarina se INR até 3,5; desde que a medicação não tenha sido iniciada recentemente e realizar nova dosagem em 1-2 semanas. No caso de alvo terapêutico de INR entre 2,5 a 3,5 o ajuste de dose deverá ser realizado para valores 0,5 maiores, exceto se INR ≥ 9,0. INR: razão normalizada internacional.

Sabe-se que quanto maior o tempo no alvo terapêutico (sigla em inglês TTR – *time in therapeutic range*), menor o risco de eventos tromboembólicos e de sangramento. Em um estudo com 119 pacientes com doença valvar mitral e FA, 78,2% dos indivíduos apresentavam INR <2,0 no momento do evento tromboembólico. Para valores de INR <1,7 a probabilidade destes dobrou e para valores abaixo de 1,5, triplicou. As dificuldades no manejo dos VKAs decorrem da grande variabilidade de dose individual, interação com alimentos e medicações, além da necessidade de monitorização frequente. Os pacientes devem ser aconselhados a evitar o consumo de álcool e manter equilíbrio na dieta, especialmente em relação aos alimentos ricos em vitamina K, como verduras e legumes verdes. Estes alimentos não devem ser excluídos da rotina alimentar.

Nos últimos anos, o papel dos anticoagulantes orais diretos (sigla em inglês DOACs – *direct oral anticoagulants*) tem se tornado progressivamente maior. A dosagem das medicações disponíveis no Brasil encontra-se no Quadro 63. Múltiplos ensaios clínicos envolvendo portadores de valvopatias estão em andamento. A maior parte das informações atuais são provenientes de análises de subgrupos dos principais estudos com os DOACs, além de estudos de coorte retrospectiva.

**Quadro 63:** Dose dos anticoagulantes orais diretos para profilaxia de eventos tromboembólicos na fibrilação atrial^177^^–^^180^

Anticoagulante	Dose habitual	Ajuste de dose	Contraindicações
Dabigatrana	150 mg 2x/dia	≥ 80 anos e/ou alto risco de sangramento: 110 mg 2x/dia	Clearance de creatinina < 30 mL/min, uso concomitante de cetoconazol
Rivaroxabana	20 mg 1x/dia	15mg 1x/dia se Clearance de creatinina < 50 mg/dL	Clearance de creatinina < 15 mL/min, doença hepática associada à coagulopatia
Apixabana	5 mg 2x/dia	2,5 mg 2x/dia em pacientes com pelo menos 2 de: idade ≥ 80 anos, peso corporal ≤ 60 kg ou creatinina sérica ≥ 1,5 mg/dL	Clearance de creatinina <15mL/min, doença hepática associada à coagulopatia
Edoxabana	60 mg 1x/dia	30mg 1x/dia	Clearance de creatinina > 95 mL/min ou < 15 mL/min

Nos portadores de próteses mecânicas, ensaios pré-clínicos envolvendo animais sugeriram que o uso de DOACs poderia ser tão seguro e eficaz quanto a varfarina. No entanto, o estudo clínico RE-ALIGN (*Dabigatran versus Warfarin in Patients with Mechanical Heart Valves*) que comparou dabigatrana *versus* varfarina foi interrompido precocemente devido a maior ocorrência do desfecho combinado acidente vascular cerebral, acidente isquêmico transitório, embolia sistêmica, infarto do miocárdio e morte (9% *versus* 5%; *hazard ratio* 1,94, IC 95% 0,64-5,86) e de sangramento (27% *versus* 12%, p < 0,05) no primeiro grupo. O estudo envolveu 252 pacientes e utilizou dabigatrana nas dosagens de 150, 220 e 300mg administrados a cada 12 horas de acordo com o clearance de creatinina, tendo sido a dose ajustada para nível sérico acima de 50 ng/mL. Sendo assim, não indicamos o uso de DOACs nos portadores de prótese mecânica. ^176^

Embora os grandes ensaios clínicos que validaram o uso dos DOACs na FA tenham excluído indivíduos com EM importante e próteses valvares mecânicas, estes estudos incluíram indivíduos com outras valvopatias. No estudo ARISTOTLE (*Apixaban versus Warfarin in Patients with Atrial Fibrillation*) 26,4% dos participantes apresentavam valvopatia moderada ou importante, no RE-LY (*Dabigatran versus Warfarin in Patients with Atrial Fibrillation*) 21,8%, no ROCKET AF (*Rivaroxaban versus Warfarin in Nonvalvular Atrial Fibrillation*) 14,1% e, por fim, no ENGAGE AF (*Edoxaban versus Warfarin in Patients with Atrial Fibrillation*) 13%. As subanálises destes estudos sugeriram eficácia dos DOACs em comparação à varfarina nos indivíduos com FA e doença valvar, excluídas as próteses mecânicas e EM importante. O ARISTOTLE e o ENGAGE-AF contemplaram indivíduos com biopróteses. ^177^^–^^180^

Apesar dos resultados negativos nos indivíduos portadores de próteses valvares mecânicas, a dabigatrana mostrou-se eficaz em prevenir a formação de trombos intracardíacos em indivíduos com prótese biológica aórtica e/ou mitral no estudo unicêntrico brasileiro DAWA (*Dabigatran Versus Warfarin After Bioprosthesis Valve Replacement for the Management of Atrial Fibrillation Postoperatively*).^175^

Uma coorte sul coreana com 2230 pacientes avaliou indivíduos com FA e EM de diferentes etiologias e gravidades anatômicas comparando o uso *off-label* dos DOACs em relação à varfarina. Os eventos isquêmicos ocorreram em 2,22%/ano no grupo DOAC versus 4,19%/ano no grupo varfarina (*hazard ratio* 0,28; IC 95% 0,18-0,45) e os sangramentos intracranianos em 0,49% no grupo DOAC versus 0,93% no grupo varfarina (*hazard ratio* 0,53; IC 95% 0,22-1,26). Este estudo reforça a hipótese da eficácia dos DOACs na EM. Deve-se ter atenção para o fato de que o tempo de INR em faixa terapêutica (*TTR – time in therapeutic range*) não foi avaliado nesta coorte. ^181^ Em um estudo observacional multicêntrico, os pacientes coreanos tinham apenas 31% de valores de INR no alvo terapêutico.

O primeiro regime antitrombótico que se adotou para os indivíduos submetidos ao TAVI em ritmo sinusal foi a dupla antiagregação com AAS e clopidogrel por 6 meses, extrapolando-se a experiência com stents e tendo em vista o período esperado para que ocorra endotelização da prótese. Uma metanálise de três ensaios clínicos pequenos recentes mostrou que a antiagregação com AAS ou clopidogrel isoladamente não mostrou aumento de mortalidade em 30 dias frente à dupla antiagregação (*odds ratio* 5,2 *versus* 3,2%, p = 0,447), bem como de eventos isquêmicos (3,8 versus 3,8%, p = 0,999), além de ter ocorrido maior chance de sangramento no grupo dupla antiagregação (*odds ratio* 2,24; IC 95% 1,12-4,46; p = 0,022). ^173^

Há evidências através de ecocardiograma transesofágico e angiotomografia computadorizada da ocorrência de espessamento de folhetos após a TAVI em até 13% dos pacientes, o que pode corresponder a formação de trombos e tem se associado ao aumento na incidência de acidentes isquêmicos transitórios e acidente vascular cerebral.^174^ Coortes observacionais nas quais os indivíduos receberam VKA ou DOACs indicaram que o uso destas medicações poderia ser seguro na prevenção de eventos. Entretanto, foi publicado recentemente o estudo multicêntrico GALILEO (*Global Study Comparing a Rivaroxaban-based Antithrombotic Strategy to an Antiplatelet-based Strategy After Transcatheter Aortic Valve Replacement to Optimize Clinical Outcomes*) que envolveu 1644 pacientes sem indicação de dupla antiagregação ou anticoagulação prévios e comparou o uso de rivaroxabana 10 mg/dia (associada a AAS 75-100 mg/dia nos primeiros 3 meses) *versus* AAS 75-100 mg (associada a clopidogrel 75mg/dia nos primeiros 3 meses). O estudo foi interrompido precocemente devido a maior ocorrência de eventos trombóticos no grupo rivaroxabana (9,8 e 7,2 por 100 pessoas-ano; *hazard ratio* 1,35, IC 95% 1,01-1,81; p = 0,04), bem como sangramentos (4,3 e 2,8 por 100 pessoas-ano; *hazard ratio* 1,5, IC 95% 0,95-2,37; p = 0,08) e mortes (5,8 e 3,4 por 100 pessoas-ano; *hazard ratio* 1,69, IC 95% 1,13-2,53). ^172^

### 15.1. Procedimentos Cirúrgicos

Nos procedimentos cirúrgicos com baixo risco de sangramento e possibilidade de hemostasia, como na cirurgia de catarata, glaucoma, dermatológicas pequenas, cirurgias odontológicas gengival, raspagem periodontal e extração de até 3 dentes simultaneamente sugere-se a manutenção da anticoagulação oral. No caso da varfarina, a INR deverá estar em faixa terapêutica, tendo sido dosado nas 24 a 48 horas que precedem o procedimento. No caso dos DOACs, idealmente o procedimento não deverá ser feito nas horas subsequentes ao uso destes medicamentos para evitar seu pico de ação.

Diante de procedimentos que impliquem em maior risco de sangramento devido ao porte cirúrgico ou dificuldade de hemostasia, está indicada a ponte com heparina nos indivíduos em uso de VKA. Fazem parte destes a cineangiocoronariografia, endoscopia ou colonoscopia com polipectomia, postectomia, vasectomia, biópsias de órgãos internos e cirurgias de maior porte. Neste caso, a varfarina deverá ser suspensa nos 5 dias que precedem o procedimento, devendo a heparina ser iniciada 3 dias antes do mesmo. No caso da heparina de baixo peso molecular, a última dose deverá ser administrada 24 horas antes do procedimento e a heparina não fracionada deverá ser suspensa 4 a 6 horas antes. A heparina em geral é reintroduzida 12 horas após, desde que a hemostasia tenha sido adequada. A varfarina, em geral, é reiniciada no dia subsequente. Deverá ser coletado INR em 5 dias e a heparina suspensa assim que for atingido o alvo terapêutico. Nas cirurgias de emergência, idealmente deverá ser administrado o complexo protrombínico 50 UI/kg intravenoso.

O rápido início da ação dos DOACs (2-4 horas) e a meia vida curta dispensam a necessidade do uso da ponte de heparina. Para procedimento eletivo de baixo risco de sangramento, recomenda-se a suspensão 24 horas antes da cirurgia, e nos casos de elevado risco de sangramento ou sítio de difícil hemostasia 48 horas antes. Na cirurgia emergencial, recomenda-se o uso do antídoto idarucizumab nos indivíduos em uso de dabigatrana na dose total de 5 g endovenosa (duas alíquotas de 2,5 g). Ainda não está disponível no Brasil o andexanet alfa (AndexXa), antídoto dos inibidores do fator Xa.

## 16. Trombose de Prótese Valvar

A trombose de prótese valvar é evento incomum, sendo mais frequente em próteses mecânicas, principalmente em posição mitral, este evento cursa com alta morbimortalidade. Pode ser assintomática ou se manifestar com síndrome de insuficiência cardíaca, baixo débito e até mesmo a morte. O diagnóstico e suspeita usualmente se dão através do ecocardiograma transtorácico, podendo ser confirmado pelo método transesofágico (Quadros 64, 65 e 66).

**Quadro 64:** Passo 1: Diagnóstico de trombose de prótese valvar

	Características da Trombose de Prótese
Avaliação clínica	Sintomas e sinais sugestivos de insuficiência cardíaca aguda/exacerbada (dispneia, dor torácica, baixo débito/síncope) Sopro compatível com valvopatia estenótica Abafamento do clique metálico Possibilidade de anticoagulação não eficaz (INR fora da faixa terapêutica)
Eletrocardiograma	Compatível com a doença de base que motivou a cirurgia valvar. Raramente com alteração aguda
Radiografia de tórax	Compatível com a doença de base que motivou a cirurgia valvar Raramente com alteração aguda na silhueta cardíaca Pode ter congestão pulmonar
Ecocardiograma	Exame fundamental para diagnóstico. Idealmente transesofágico. Documentação de trombo aderido à prótese, identificação do local e tamanho da trombose
Estudo hemodinâmico (fluoroscopia)	Mobilidade inadequada de um ou mais folhetos da prótese mecânica

INR: razão normalizada internacional.

**Quadro 65:** Passo 2: Avaliação da etiologia da trombose de prótese valvar

	Características etiológicas
Anticoagulação ineficaz	Interrupção da anticoagulação Interação medicamentosa/comportamental INR abaixo do alvo terapêutico

INR: razão normalizada internacional.

**Quadro 66:** Passo 3: Avaliação de sintomas da trombose de prótese valvar

	Sintomas
Dispneia	Principal sintoma Distinguir entre discreta piora CF I e sintomas mais nítidos (CF II-IV)
Dor Precordial	Possibilidade de embolia de coronária
Baixo débito/Síncope	Indicativo de obstrução importante

CF: classe funcional.

O principal complicador da trombose é o tamanho do trombo devido ao risco de embolia e obstrução valvar (Quadro 67).

**Quadro 67:** Passo 4: Avaliação de complicadores da trombose de prótese valvar

	Complicadores
Alto risco de embolização associada à trombólise	Trombo > 8 mm Trombo móvel (pedunculado)
Hipertensão pulmonar	PSAP ≥ 50 mmHg em repouso Mais frequente nos casos de EM associada Clinicamente – sintomas de insuficiência cardíaca direita Relação com aumento do risco cirúrgico
FA de início recente	Relação com remodelamento importante do AE

AE: átrio esquerdo; EM: estenose mitral; FA: fibrilação atrial; PSAP: pressão sistólica da artéria pulmonar.

As recomendações das diretrizes internacionais são heterogêneas em relação ao tratamento e há carência de estudos randomizados nesta área (Quadros 68 e 69). Na trombose de prótese sem repercussão hemodinâmica significativa (CF I e II) e sem obstrução de fluxo valvar nos exames complementares, é indicada anticoagulação oral e monitorização ambulatorial com imagem. No caso de trombo grande (especialmente maior do que 8mm) e/ou móvel, portanto, com elevado risco de embolização, está indicada internação hospitalar para anticoagulação venosa. Não havendo resolução do trombo em exames de imagem realizados a cada 5 a 7 dias, pode ser considerada fibrinólise e/ou cirurgia.^184^^,^^185^

**Quadro 68:** Passo 5: Tipo de intervenção da trombose de prótese valvar^184^^,^^185^

Tipo	Considerações
Trombólise	Terapia prioritária rTPA 10 mg em bolus seguido por 90 mg em 2 horas OU Estreptoquinase 500.000 UI em 20 minutos, seguido de 1.500.000 UI em 10 horas
Cirurgia valvar	Reservada para casos com alto risco de complicações hemorrágicas ou embólicas associadas à trombólise

rTPA: recombinant tissue plasminogen activator.

**Quadro 69:** Trombose de prótese: Recomendações^1^^,^^2^^,^^184^^,^^185^

Intervenção	Condição clínica	SBC	AHA	ESC
Trombólise	Trombose valvar em câmara direita	IIa B	IIa B	–
	Trombo pequeno (< 0,8 cm²), NYHA I-III, câmaras esquerdas se persistência do trombo após heparinização endovenosa	IIa B	IIa B	–
Cirurgia valvar	NYHA IV, câmaras esquerdas	IB	I B	I C
	Trombo móvel ou grande (> 0,8 cm²), câmaras esquerdas	IIa C	IIa C	IIa C (trombo >10mm)

NYHA: New York Heart Association.

Nos casos em que há comprometimento hemodinâmico mais significativo (CF III e IV), habitualmente são indicadas administração de terapia fibrinolítica ou cirurgia valvar. Recentemente, há tendência de priorização da fibrinólise frente à cirurgia, com base em dados de metanálise de 48 estudos. Na decisão sobre estas últimas duas estratégias, é recomendada discussão em *Heart Team*, devendo ser pesados riscos da fibrinólise (procedimento de primeira escolha) e da cirurgia individualmente. Favorecem a fibrinólise: alto risco cirúrgico, baixo risco de sangramento, acometimento de valvas direitas, primeiro episódio de trombose valvar, trombo menor do que 1cm². Havendo instabilidade hemodinâmica, o tratamento de escolha é a cirurgia, podendo ser considerada a fibrinólise em indivíduos de elevado risco cirúrgico. Os fatores que favorecem o procedimento cirúrgico são: contraindicação à fibrinólise, alto risco de sangramento, baixo risco cirúrgico, suspeita de *pannus* associado à trombose e necessidade de outros procedimentos cirúrgicos cardíacos concomitantes (ex: revascularização do miocárdio).^184^^,^^185^

## 17. Profilaxia da Febre Reumática

A FR, e consequente a Cardiopatia Reumática Crônica, permanece como a mais importante causa de valvopatia adquirida no Brasil. A Doença Reumática é das que acarretam maiores custos para o Sistema Único de Saúde e para a comunidade em geral, pois acomete indivíduos muito jovens e frequentemente determina múltiplas internações hospitalares e cirurgias. Permanece como a principal causa de valvopatia adquirida em nosso meio. Diminuir esta incidência permanece objetivo importantíssimo, considerado que esta, das doenças cardiológicas, é com certeza a mais facilmente prevenível.

### 17.1. Profilaxia Primária da Febre Reumática

Para diminuir a incidência de FR, a medida de maior impacto é a profilaxia primária, impedindo que os indivíduos suscetíveis venham a contrair a doença (Quadros 70 e 71). Recentemente encontramos dificuldades sérias para realizar a profilaxia primária: o suprimento de Penicilina G benzatina está errático, com frequentes desabastecimentos da medicação. Além disso, restrições a locais de aplicação da medicação por preocupações com reações alérgicas e falta de familiaridade de profissionais de saúde primária com a aplicação intramuscular fazem com que seja cada vez mais difícil a realização da profilaxia primária por via intramuscular. Este fato irá certamente contribuir para o aumento da incidência da doença nos próximos anos.

**Quadro 70:** Regimes terapêuticos indicados para a faringoamigdalite estreptocócica – profilaxia primária da febre reumática^186^^–^^193^

Medicação		Dose	Via de administração / Duração	Comentários
**Penicilinas e derivados**				
	Benzilpenicilina G benzatina	600.000 UI até 25 kg, 1.200.000 UI acima de 25 kg	Intramuscular Dose única	Medicação de escolha: dose única, alta eficácia e baixo custo
	Amoxicilina	50 mg/kg para crianças e 1,5g diárias para adultos, divididos em 2 a 3 tomadas	Via Oral 10 dias	Baixa aderência ao tratamento completo
	Fenoximetilpenicilina	250 mg 2 a 3x ao dia até 25 kg, 500 mg 3x ao dia > 25 kg	Via Oral 10 dias	Baixa aderência ao tratamento completo
**Para pacientes alérgicos à penicilina**				
	Clindamicina	20 mg/kg dividido 3x ao dia, adultos: 300 a 600 mg 3x ao dia	Via Oral 10 dias	Frequente intolerância gastrointestinal
	Azitromicina	12 mg/kg em dose única diária. Para adultos, 500 mg 1x ao dia	Via Oral 5 dias	Única antibioticoterapia por via oral que pode erradicar o estreptococo em menos de 10 dias
	Claritromicina	15 mg/kg 2x ao dia ou para adultos, 250 mg 2x ao dia	Via Oral 10 dias	

IM: insuficiência mitral.

**Quadro 71:** Recomendações para profilaxia primária da febre reumática^186^^–^^193^

**Classe I** – Benzilpenicilina G benzatina para pacientes com amigdalite estreptocócica – Benzilpenicilina G benzatina para pacientes com suspeita de amigdalite estreptocócica, mesmo sem confirmação diagnóstica – Antibioticoterapia por via oral para pacientes com amigdalite estreptocócica naqueles pacientes alérgicos à penicilina
**Classe IIa** – Uso de antibióticos por via oral para tratamento de faringoamigdalite estreptocócica em pacientes não alérgicos à penicilina – Realização de testes rápidos para a detecção de estreptococos em orofaringe para decisão sobre tratamento com penicilina.
**Classe III** – Realização de cultura de orofaringe em pacientes com suspeita de amigdalite para decisão sobre tratamento com peniclina.

Terapias por via oral não devem ser usadas rotineiramente, pois em geral é necessário o uso de 10 dias de terapêutica para a completa erradicação dos estreptococos da orofaringe. Por isso há um risco muito grande da não aderência ao tratamento completo, fazendo com que o paciente tenha risco de desenvolver surto reumático. Tratamentos com base em 5 dias de Azitromicina foram propostos, mas ainda não possuem estudo clínico validando seu uso em faringoamigdalites.^186^^–^^193^

### 17.2. Profilaxia Secundária da Febre Reumática

Para pacientes que já tem o diagnóstico de FR, é indicada a profilaxia secundária para a prevenção de novos surtos de FR (Quadros 72 e 73). A droga de escolha é a Benzilpenicilina G benzatina, nas mesmas doses de 600.000 UI para crianças com até 27 kg e 1.200.000 UI acima deste peso, com intervalo máximo de três semanas. Aplicações mensais de Penicilina Benzatina não proporcionarem proteção adequada aos portadores de doença reumática em países com alta endemicidade da doença como o nosso.^194^^–^^198^ Para pacientes com alergia a penicilina está indicada a Sulfadiazina, na dose de 1 g/dia, sendo necessário o controle de possíveis quadros leucopênicos.

**Quadro 72:** Profilaxia secundária para a febre reumática: Medicações recomendadas e posologia^194^^–^^200^

Medicação		Dose e periodicidade	Recorrência/Notas
	Benzilpenicilina G benzatina	< 25 kg – 600.000UI > 25 kg – 1.200.000 UI 15/15 dias nos dois primeiros anos do surto 21/21 dias nos anos subsequentes	Recorrência de 0,3% ao ano Medicação de escolha
	Fenoximetilpenicilina	250 mg por boca 2x ao dia	Recorrência de 5% ao ano – não deve ser usada como alternativa à penicilina G benzatina
Para pacientes alérgicos à penicilina	Sulfadiazina	< 25 kg – 500 mg ao dia > 25 kg – 1g ao dia	Recorrência de 1,3% ao ano Pode ser usado até concluída dessensibilização à penicilina
Para alérgicos à Penicilina e à sulfadiazina	Eritromicina	250 mg 2x ao dia	Regime de profilaxia empírico, não foi objeto de estudos em profilaxia secundária da FR – só deve ser usado excepcionalmente.

FR: febre reumática.

**Quadro 73:** Recomendações para a profilaxia secundária da febre reumática^194^^–^^200^

**Classe I** – Benzilpenicilina G benzatina para profilaxia secundária de FR, de 15/15 dias nos dois primeiros anos após o surto e de 21/21 dias nos anos subsequentes. – Uso de Benzilpenicilina G benzatina até os 18 anos, ou 5 anos após o último surto em pacientes com FR sem cardite. – Uso de Benzilpenicilina G benzatina até os 25 anos, ou 10 anos após o último surto em pacientes com FR com cardite, mas sem sequelas cardíacas ou sequelas leves, desde que não sejam lesões estenóticas. – Uso de até os 40 anos e em pacientes com FR com cardite e com sequelas graves ou cirurgia cardíaca para correção da valvopatia. – Uso de Benzilpenicilina G benzatina após os 40 anos em pacientes com exposição ocupacional a estreptococos. – Sulfadiazina para antibioticoprofilaxia da FR em pacientes alérgicos à penicilina
**Classe IIa** – Uso de antibioticoprofilaxia via oral para pacientes com FR em pacientes não alérgicos à penicilina
**Classe IIb** – Uso de eritromicina para antibioticoprofilaxia para pacientes com FR em pacientes alérgicos à penicilina e às sulfas
**Classe III** – Suspensão da antibioticoprofilaxia para FR após a realização de cirurgia cardíaca com implante de prótese (s) valvar (es), mesmo com demais valvas sem lesão aparente.

FR: febre reumática.

A alternativa frente ao desabastecimento recente de Penicilina G benzatina é a sulfadiazina, que frequentemente está disponível para doenças reumatológicas no sistema público de saúde, listada nos regimes de medicação de alto custo. Devemos lembrar também que apenas a Penicilina G Benzatina e a Sulfadiazina possuem estudos controlados com eficácia comprovada para a profilaxia secundária da FR.^199^^–^^200^

### 17.3. Os Critérios de Suspensão das Profilaxias (Quadro 74)

**Quadro 74:** Duração da profilaxia secundária para febre reumática

Categoria	Duração
FR sem cardite: quadros puros de artrite ou coréia	Até os 18 anos ou 5 anos após o último surto de FR, o que for mais longo
FR com cardite, mas sem sequelas ou com sequelas valvares muito leves (exceto lesões estenóticas, mesmo que leves)	Até os 25 anos ou 10 anos após o último surto
FR com cardite e sequelas graves. Pacientes submetidos à cirurgia cardíaca	Até os 40 anos no mínimo. Por toda a vida se exposição ocupacional.

FR: febre reumática.

– Pacientes sem acometimento cardíaco, apenas com manifestação articular ou coréia “pura” – suspender aos 18 anos ou 5 anos após o surto reumático;
– Pacientes com cardite durante o surto agudo que não apresentam sequelas tardias ou apresentam sequelas muito discretas – suspender aos 25 anos ou dez anos após o último surto reumático;
– Pacientes nos quais é retirada a profilaxia e os sintomas retornam deverão ter profilaxia mantida por mais 5 anos.
– Pacientes com acometimento cardíaco, mesmo discreto, deverão ter profilaxia prolongada, de preferência por toda a vida, e quando isso não for possível até a quarta década. Devemos quando da suspensão da medicação sempre pesquisar sobre contato ocupacional com fontes de estreptococos.

## 18. Profilaxia de Endocardite Infecciosa nas Valvopatias

A EI é complicação grave das valvopatias, sendo frequentemente fatal. Desta forma, havendo a possibilidade de fazer profilaxia para tal entidade, a mesma deveria ser aplicada. Com este intuito, foram utilizados vários esquemas antibióticos, porém com pouca evidência em estudos controlados, principalmente pela dificuldade em se realizar estudo controlado de grande porte com medicações já em domínio público.

Os estreptococos fazem parte da flora normal da orofaringe e trato gastrointestinal e causam pelo menos 50% das EI adquiridas na comunidade em nosso meio. Demonstrou-se bacteremia pelos estreptococos do grupo viridans em até 61% dos pacientes, após extração dentária e cirurgia periodontal (36% a 88%). E estudos experimentais em animais mostraram que a profilaxia antibiótica era capaz de evitar EI por estreptococos viridans e enterococos.^201^^,^^202^

Mais recentemente tem se notado que há bacteremias espontâneas, de origem especialmente dentária e gengival, em situações do dia-a-dia. Assim, atividades prosaicas rotineiras, como escovação de dentes (0 a 50%), uso de fio dental (20% a 68%), uso de palito de dentes e mesmo mastigação de refeição (7% a 51%), são associadas à bacteremia. Desse modo, a carga de bacteremia espontânea, não determinada por intervenção odontológica, seria maior do que a determinada por tratamentos dentários. Um estudo teórico da bacteremia cumulativa, durante cerca de um ano, calculou que a bacteremia do dia-a-dia é seis vezes maior do que a bacteremia causada por uma extração dentária isolada. Considerando que a indicação de profilaxia dentária recomenda duas visitas por ano ao dentista, percebe-se um impacto das atividades do dia-a-dia na geração de bacteremias muito maior do que a própria intervenção dentária. Trabalhos epidemiológicos recentes não mostram relação entre tratamento dentário duas semanas antes e episódios de EI.^203^^–^^208^

Por este motivo, mais importante que a profilaxia antes de procedimentos dentários é a manutenção de ótima saúde bucal em valvopatas. Aqueles com boa saúde bucal tem menor possibilidade de bacteremia em atividades cotidianas. Assim devemos focar mais na prevenção não farmacológica que na profilaxia farmacológica. Faz parte da profilaxia não farmacológica da EI reforçar em todas as consultas a necessidade de se manter uma ótima saúde bucal e aumentar a frequência das consultas odontológicas, de duas (recomendação para a população em geral) para quatro vezes ao ano. Devemos ressaltar que muitas das afecções odontológicas que mais causam EI são oligossintomáticas, como a gengivite e lesões periapicais endodônticas.^209^

Para pacientes submetidos a intervenções dentárias, existe crescente evidência de que a profilaxia antibiótica previne apenas um número muito pequeno de casos. Entretanto há evidências recentes que abolir totalmente a profilaxia antibiótica pode levar ao aumento da incidência da EI. O Instituto Nacional de Saúde e Excelência Clínica (NICE – sigla em inglês), instituição britânica, propôs que não seja realizada profilaxia para EI em nenhuma ocasião.^210^ Como consequência observou-se uma diminuição da prescrição da profilaxia antibiótica antes de tratamentos dentários seguindo de um aumento do número de casos de EI.^211^ Assim temos evidência empírica que abolir o completamente a profilaxia antibiótica pode levar a um aumento dos casos de EI. Desta maneira preconizamos a manutenção da profilaxia antibiótica antes de procedimentos dentários, gastrointestinais e geniturinários.

Todo paciente com valvopatia moderada a importante, seja de etiologia reumática, degenerativa, ou portador de prótese valvar deve realizar profilaxia não farmacológica e farmacológica para EI, visto que todos pacientes com EI apresentam alta morbimortalidade.

### 18.1. Profilaxia Não Farmacológica da Endocardite Infecciosa

A profilaxia não farmacológica da EI pode ser mais eficaz que a farmacológica por atuar na prevenção primária de fontes comprovadas de bacteremia (Quadro 75). Destacamos como medidas prioritárias para o valvopata manutenção de ótima saúde bucal, evitar procedimentos de arte corporal invasiva, como o implante de *piercings* e tatuagens.

**Quadro 75:** Profilaxia não farmacológica da endocardite infecciosa

Recomendação	Grau de recomendação	Nível de evidência
Reforço da necessidade de manter boa saúde bucal e hábitos adequados de higiene durante consultas médicas.	I	C
Consultas odontológicas trimestrais	I	C
Tatuagem	III	C
*Piercings* em pele	III	C
*Piercings* língua e mucosas	III	C

Com relação à arte corporal (procedimentos como tatuagens e *piercings*) a mesma deve ser contraindicada. O *piercing* leva à formação de um trajeto que precisa ser epitelizado, e até este processo ser completo é fonte de contínua bacteremia, sendo abundantes na literatura relatos de endocardite, inclusive com desfecho fatal, relacionado a implantes de *piercings*. É importante que os pacientes sejam informados dos riscos do procedimento, assim como os médicos devem sempre abordar esse assunto quando atendem pacientes que tem ou que pretendem ter arte corporal.^212^

### 18.2. Profilaxia da Endocardite Infecciosa para Procedimentos Dentários (Quadros 76, 77 e 78)

**Quadro 76:** Indicações de profilaxia para procedimentos dentários

Com alta probabilidade de bacteremia significativa	Sem alta probabilidade de bacteremia significativa
Procedimentos que envolvem a manipulação de tecido gengival, região periodontal ou perfuração da mucosa oral.	Anestesia local em tecido não infectado
	Radiografia odontológica
	Colocação ou remoção de aparelhos ortodônticos
	Ajuste de aparelhos ortodônticos
	Colocação de peças em aparelhos ortodônticos
	Queda natural de dente-de-leite
	Sangramento oriundo de trauma da mucosa oral ou lábios

**Quadro 77:** Profilaxia antibiótica de endocardite em valvopatias

Indicação	Recomendação	Nível de evidência
Pacientes com valvopatia moderada e importante, assim como portadores de prótese valvar, e que serão submetidos a procedimentos odontológicos de alta probabilidade de bacteremia significativa.	I	C
Pacientes com risco elevado para endocardite infecciosa grave* e que serão submetidos a procedimentos geniturinários ou gastrointestinais associados à lesão de mucosa.	IIa	C
Pacientes com risco elevado para endocardite infecciosa grave* e que serão submetidos a procedimentos esofágicos ou do trato respiratório associado à lesão de mucosa.	IIa	C
Pacientes com PVM sem regurgitação, pacientes após cirurgia de revascularização miocárdica ou após colocação de stents, com sopros cardíacos inocentes, portadores de marcapasso ou desfibrilador, com doença de Kawasaki ou FR sem disfunção valvar, que serão submetidos a procedimentos odontológicos, do trato respiratório, geniturinário ou gastrointestinal.	III	C
Pacientes submetidos a procedimentos que não envolvam risco de bacteremia.	III	C

* Risco elevado para EI grave: prótese valvar cardíaca, EI prévia, cardiopatia congênita não reparada ou corrigida parcialmente ou corrigida com material protético, transplantado cardíaco com valvopatia. FR: febre reumática; PVM: prolapso da valva mitral.

**Quadro 78:** Esquemas de profilaxia para endocardite infecciosa antes de procedimentos dentários

Via de administração	Medicação	Dose única 1 hora antes do procedimento	
		**Criança**	**Adulto**
Oral	Amoxicilina	50 mg/kg	2 g
Oral (alergia à penicilina)	Clindamicina	20 mg/kg	600 mg
	Azitromicina ou claritromicina	15 mg/kg	500 mg
Parenteral (endovenoso ou intramuscular)	Ampicilina	50 mg/kg	2 g
	Cefazolina ou ceftriaxone	50 mg/kg	1 g
Parenteral (endovenoso ou intramuscular) (alergia à penicilina)	Clindamicina	20 mg/kg	600 mg

IM: insuficiência mitral.

O antibiótico deve ser ministrado, uma hora antes do procedimento. O regime usado deve impedir a bacteremia por estreptococos viridans sempre que for manipulado tecido da gengiva ou da região periapical do dente. O antibiótico de escolha, se não houver alergia, é a amoxicilina, por sua absorção adequada e pela suscetibilidade do agente infeccioso. No entanto, têm sido descritas resistências de várias cepas desse micro-organismo a esse antibiótico. Para pacientes alérgicos a penicilina utiliza-se clindamicina, azitromicina ou claritromicina.

### 18.3. Profilaxia da Endocardite Infecciosa para Procedimentos no Trato Respiratório

Pacientes a serem submetidos à incisão ou biópsia da mucosa do trato respiratório, como cirurgias otorrinolaringológicas, devem receber esquema antibiótico semelhantes ao utilizados para afecções da boca.

### 18.4. Profilaxia da Endocardite Infecciosa para Procedimentos nos Tratos Geniturinário e Gastrointestinal

Os enterococos fazem parte da flora normal do trato gastrointestinal e podem causar EI. Assim, considerando a falta de adequada evidência científica, as diretrizes americanas e europeias passaram a não mais indicar profilaxia antibiótica antes de intervenções nestas localizações.^213^^,^^214^ No entanto, considerando a gravidade de uma eventual ocorrência de EI decorrente destas fontes, foi optado, no atual documento, por considerar a profilaxia para pacientes com risco elevado para EI grave e que serão submetidos a procedimentos geniturinários ou gastrointestinais associados a lesão de mucosa (Quadro 79).^215^ Na presença de infecções instaladas nos tratos geniturinário e gastrointestinal, o tratamento deve incluir antibióticos que ajam contra o enterococo.

**Quadro 79:** Profilaxia antibiótica parenteral para procedimentos do trato gastrointestinal e geniturinário

Via de administração	Medicação	Dose única 1 hora antes do procedimento		
		Criança		Adulto
Parenteral (endovenoso)	Ampicilina +	50 mg/kg		2 g
	Gentamicina		1,5 mg/kg	
Parenteral (endovenoso) - alergia à penicilina	Vancomicina +	20 mg/kg		1 g
	Gentamicina		1,5 mg/kg	

## 19. Gravidez, Planejamento Familiar e Contracepção

### 19.1. Aconselhamento Prévio à Gravidez

A estratificação do risco das doenças valvares para o planejamento de gravidez deve ser fundamentada no diagnóstico anatômico da lesão valvar que classifica a gestação em riscos alto, intermediário e aceitável (Quadro 80).

**Quadro 80:** Classificação dos riscos das doenças valvares para gravidez

Risco Alto	Risco intermediário	Risco aceitável
EM importante	Prótese biológica com disfunção moderada	Doença valvar discreta
EAo importante Prótese biológica estenótica/calcificada Prótese mecânica com disfunção	Estenose valvar pulmonar	Prótese biológica sem disfunção
	Prótese mecânica mitral > Prótese mecânica aorta	Sem fatores complicadores

EAo: estenose aórtica; EM: estenose mitral.

A concomitância de fatores complicadores deve ser considerada como agravante do prognóstico materno e fetal, (Quadro 81).^216^

**Quadro 81:** Condições agravantes do prognóstico da gravidez em portadoras de doença valvar^216^

Fatores complicadores: FA, HP, disfunção ventricular, eventos prévios (insuficiência cardíaca, tromboembolismo, endocardite infecciosa) Lesões obstrutivas à esquerda de grau moderado à importante Doenças de aorta associada com diâmetros aumentados de aorta ascendente Síndrome de Marfan (Diâmetro de aorta > 40 mm) Valva aórtica bicúspide (Diâmetro de aorta > 45 mm) NYHA CF III/IV Doença valvar com indicação de intervenção cirúrgica ou percutânea Necessidade do uso de anticoagulantes (transitória ou permanente)

CF: classe funcional; FA: fibrilação atrial; HP: hipertensão pulmonar; NYHA: New York Heart Association.

No planejamento de gravidez, considera-se que a intervenção percutânea ou cirúrgica deva ser indicada em pacientes com doença valvar importante, mesmo em pacientes assintomáticas, porque CF I/II não assegura boa evolução materna, em lesões obstrutivas graves. (Quadro 82).^217^

**Quadro 82:** Recomendações para conduta em doença valvar nativa no planejamento familiar e durante a gestação^217^

Doença valvar	Planejamento Familiar Intervenção	Gestação		
		Risco Materno	Risco fetal	Intervenção
Estenose mitral importante AVM < 1,5 cm^2^	Considerar VMCB ou cirurgia: CF III/IV ou CF I/II + PSAP > 50 mmHg ou FA início recente	Risco aumentado: CF III/IV e/ou FA	Prematuridade Restrição de crescimento intrauterino Perda fetal Aumentado em CF III/IV	Betabloqueador Diurético Anticoagulação se FA Se refratária CF materna III/IV considerar VMCB ou cirurgia
Estenose aórtica importante AVA ≤ 1 cm^2^	Considerar valvoplastia cateter-balão ou cirurgia: Sintomática ou Assintomática + Teste ergométrico alterado ou FEVE < 50% ou AVA < 0,7 cm^2^ gradiente médio > 60 mmHg ou Valva bicúspide + diâmetro de aorta > 45 mm	Risco aumentado IC Arritmia Síncope Morte súbita Dissecção Ao	Complicações Prematuridade Restrição de crescimento intrauterino Perda fetal	Repouso Uso de diuréticos é controverso Considerar betabloqueador ou bloqueador do canal de cálcio + Anticoagulação se FA Considerar valvoplastia cateter-balão ou cirurgia se insuficiência cardíaca ou síncope
Insuficiência mitral importante	Considerar cirurgia (plástica/prótese): CF ≥ II ou Assintomática + FEVE ≤ 60% + PSAP ≥ 50 mmHg + DSVE ≥ 40 mm	Insuficiência cardíaca FA Risco aumentado se FEVE < 35%	Baixo risco	Diurético, vasodilatador Digoxina, Betabloqueador Considerar cirurgia ou clipagem percutânea mitral^®^ se insuficiência cardíaca refratária
Insuficiência Aórtica importante	Considerar cirurgia: Sintomática CF ≥ II ou Fatores complicadores FEVE < 50% DDVE > 70 mm (75 se reumática) DSVE > 50 mm (55 se reumática) Considerar intervenção em aorta proximal: Valva bicúspide isolada diâmetro de aorta > 45 mm	Baixo risco se assintomática e FEVE normal Risco de Insuficiência cardíaca e/ou FA se CF > II ou FEVE < 35%	Baixo risco	Diurético, vasodilatador, Digoxina Considerar cirurgia se insuficiência cardíaca refratária Considerar intervenção em aorta proximal: Valva bicúspide isolada com diâmetro de aorta > 45 mm

AVA: área valvar aórtica; AVM: área valvar mitral; CF: classe funcional; DDVE: diâmetro diastólico do ventrículo esquerdo; DSVE: diâmetro sistólico do ventrículo esquerdo; FA: fibrilação atrial; FEVE: fração de ejeção do VE; IC: insuficiência cardíaca; PSAP: pressão sistólica da artéria pulmonar; VMCB: valvoplastia mitral por cateter-balão.

Em contrapartida, as lesões de regurgitação apresentam melhor prognóstico quando a fração FEVE é preservada e os raros casos que complicam são aqueles que já apresentavam indicação cirúrgica prévia a gestação.

Durante a gestação, o princípio básico para a prevenção e tratamento das complicações é priorizar as medidas gerais e selecionar fármacos não teratogênicos com doses ajustadas à idade gestacional. O Quadro 83 apresenta os fármacos e doses diárias mais frequentemente utilizados no controle das complicações da doença valvar durante a gestação.^218^

**Quadro 83:** Recomendações Gerais e farmacológicas durante a gestação^218^

Restrição de atividades físicas e dieta hipossódica (4 g/dia) Profilaxia da doença reumática deve ser mantida (exceto a sulfadiazina) Se indicado tratamento farmacológico, considerar: ○ Diurético: Furosemida (< 80 mg/dia) ○ Betabloqueador: Propranolol (< 80 mg/dia) ou Succinato de Metoprolol (< 100 mg/dia), carvedilol < 50 mg ○ Bloqueador dos canais de cálcio não dihidropiridínicos: Verapamil (< 240 mg/dia) ○ Vasodilatador: Hidralazina (< 100 mg/dia) ○ Digital: digoxina (0,25 mg/dia)

Medidas intervencionistas em doenças valvares durante a gestação são reservadas para os casos refratários ao tratamento clínico. Os procedimentos percutâneos devem ser preferidos à cirurgia e as propostas de tratamento devem ser discutidas pelo *Heart Team,* compartilhada com a equipe obstétrica. A valvoplastia por cateter balão na EAo tem sido indicada quando a etiologia é congênita ou na tentativa de resgate da vida materna em casos extremos de gravidade. Em contrapartida a VMCB é segura com resultados equivalentes aos da cirurgia, contudo requer critérios clássicos de indicação tais como a ausência de trombo em AE, IM no máximo de grau discreto e escore ecocardiográfico de Wilkins-Block ≤ 8.

### 19.2. Próteses Valvares

Do ponto de vista hemodinâmico, tanto as próteses mecânicas como as biológicas melhoram a capacidade funcional e proporcionam semelhante evolução clínica durante a gravidez, contudo a prótese biológica parece ser mais vantajosa porque não requer anticoagulação (Quadro 84). A durabilidade limitada com possibilidade de reoperação em curto prazo, inclusive durante a gravidez, são as maiores restrições no implante de próteses biológicas em mulheres jovens.

**Quadro 84:** Prótese valvar com função normal e riscos para a gestação

Prótese biológica com FEVE normal		Prótese mecânica com FEVE normal	
Risco materno	Risco fetal	Risco materno	Resultados fetais
Risco baixo	Risco baixo	Risco intermediário Requer anticoagulação	Alto risco
Não requer anticoagulação		Embolia sistêmica Trombose de prótese Hemorragia	Embriopatia varfarínica Perdas fetais Prematuridade Hemorragia perinatal

FEVE: fração de ejeção do VE.

A conduta perante a disfunção de prótese durante a gravidez deve sempre priorizar a vida materna e as propostas devem ser discutidas com *Heart Team,* e compartilhadas com a equipe obstétrica (Quadro 85).

**Quadro 85:** Conduta em próteses com disfunção durante a gestação

Prótese biológica		Prótese mecânica	
Risco materno	Risco fetal	Risco materno	Risco fetal
Disfunção com predomínio de insuficiência, CF I/II e FEVE normal Considerar medidas farmacológicas	Risco baixo	Disfunção com insuficiência “paravalvar” leve a moderada, sem hemólise significativa ou insuficiência cardíaca grave Considerar medidas farmacológicas para insuficiência cardíaca e anemia IM severa ou hemólise significativa Considerar intervenção Insuficiência cardíaca e/ou hemólise sintomáticas Considerar fechamento percutâneo do “leak” paravalvar ou cirurgia (alto risco de recidiva)	Alto risco fetal se cirurgia
Disfunção com predomínio de estenose valvar com calcificação (mitral, aórtica ou tricúspide) Riscos de insuficiência cardíaca grave, choque, morte súbita Sempre considerar Implante percutâneo ou transapical (*valve-in-valve*) ou cirurgia	Alto risco fetal Perda fetal Prematuridade	Trombose prótese mecânica Considerar intervenção de emergência (trombólise ou cirurgia) Estenose de prótese mecânica por crescimento endotelial intravalvar- *Pannus ou mismatch* Necessidade de intervenção é rara Se necessário, considerar cirurgia	Alto risco fetal se cirurgia

CF: classe funcional; FEVE: fração de ejeção do VE; IM: insuficiência mitral.

O esquema da anticoagulação para pacientes portadoras de prótese mecânica é ainda controverso.^218^^,^^219^ Até o momento não há uma orientação uniforme e aceita amplamente. Fatores a serem considerados incluem: preferência da paciente, *expertise* do médico assistente, recursos no atendimento e disponibilidade de controle adequado da coagulação.

**Figura 11:**
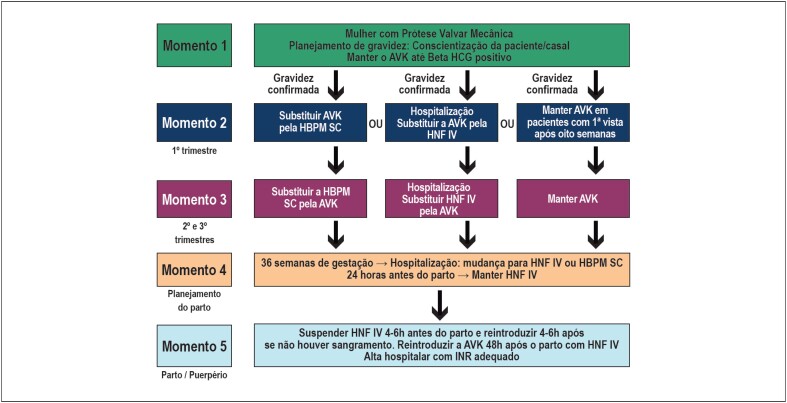
Algoritmo com recomendações para anticoagulação em portadoras de próteses mecânicas para gravidez, parto e puerpério. AVK: antagonista da vitamina K, HCG: gonadotrofina coriônica, HBPM SC: heparina de baixo peso molecular subcutânea; HNF IV: heparina não fracionada intravenosa.

As recomendações para a prevenção de tromboembolismo em próteses mecânicas pretendem atender aos requisitos ideais de um posicionamento baseadas na documentação da literatura, na vivência dos autores e que seja efetiva para a realidade dos diversos Serviços. Entende-se que a dinâmica da anticoagulação permanente para portadoras de próteses mecânicas seja multidisciplinar e fragmentada em cinco momentos: preconcepção, cada trimestre, parto e puerpério, apresentada no Quadro 86 e Figura 11. O rigoroso controle da anticoagulação e doses dos anticoagulantes devem ser ajustados de acordo com as metas convencionais.

**Quadro 86:** Controles da anticoagulação em prótese mecânica durante a gravidez

Idade gestacional (semanas)	Anticoagulante	Controle
Entre 6ª e 12ª	Heparina de baixo peso molecular 1,0 mg/kg subcutânea 12/12 horas ou Heparina não fracionada endovenosa 18 UI/kg/hora em bomba infusão (< 30.000 UI)	Anti-Xa: 0.8-1.2 U/ml/ TTPa 1,5 vez a 2,0 vezes do valor controle
12ª até a 36ª	Varfarina na dose de acordo com INR	Aórtica INR entre 2,5 e 3,0 Mitral INR 3,0 e 3,5
Após 36ª até o parto	Heparina de baixo peso molecular 1,0 mg/kg subcutânea 12/12 horas ou Heparina não fracionada endovenosa 18 UI/kg/hora em bomba de infusão (< 30.000 UI)	Anti-Xa: 0.8-1.2 U/ml TTPa 1,5 vez a 2,0 vezes do valor controle
Puerpério	Heparina de baixo peso molecular 1,0 mg/kg subcutânea 12/12 horas Heparina não fracionada endovenosa 18 UI/kg/hora em bomba de infusão (< 30.000 UI) Varfarina alcançar INR alvo para alta hospitalar	Anti-Xa: 0.8-1.2 U/ml TTPa 1,5 vez a 2,0 vezes do valor controle INR entre 2,0 e 2,5

TTPa: tempo de tromboplastina parcial ativada; INR: razão normalizada internacional.

Momento 1 - *Orientação quanto ao diagnóstico precoce da gravidez:* Esclarecimento sobre a obrigatoriedade em manter a anticoagulação, a disponibilidade dos anticoagulantes e os seus riscos em todas as etapas da gestação, parto e puerpério. A orientação inclui a informação sobre a importância do diagnóstico precoce da gravidez em reduzir a ocorrência da embriopatia, que ocorre entre 6ª e 9ª semana de gestação. Nesta consulta é fornecido um pedido de exame para a dosagem de gonadotrofina coriônica beta (βHCG) que deve ser realizado à primeira dúvida de atraso menstrual.

Momento 2 - *Primeiro trimestre:* confirmado o diagnóstico da gravidez (βHCG e ultrassonografia obstétrica), deve ser realizado a substituição da varfarina pela heparina que permite uma conciliação entre o benefício da prevenção de trombose materna e o malefício da embriopatia. Em pacientes que chegam na 1ª visita médica com idade gestacional além da 6ª semana, a varfarina não deve ser suspensa. O casal deve ser informado da possibilidade da embriopatia e que os riscos da substituição pela heparina, neste momento, não são mais justificados.

Momento 3 - *Segundo trimestre:* retorno ao anticoagulante oral. O retorno para à varfarina ajusta-se à suposição de abreviar o uso da heparina e do menor risco da embriopatia. A proposta é manter as doses de varfarina de acordo com as metas de anteriores à gestação, com controle da INR semanal ou quinzenal. A reintrodução da varfarina deve obedecer a dinâmica da transição, ou seja, simultânea à heparina de baixo peso molecular subcutânea ou à heparina não fracionada endovenosa até o alcance da meta da INR.

Momento 4 - *terceiro trimestre:* considerar a hospitalização, redirecionar para a anticoagulação parenteral e planejar o parto. A hospitalização deve ser programada com 36 semanas de gestação para o uso de heparina de baixo peso molecular subcutânea ou heparina não fracionada endovenosa.

Momento 5 - *puerpério:* reintrodução da anticoagulação oral e alta hospitalar. Decorrido 6 horas do parto e em ausência de complicação materna, a heparina não fracionada endovenosa ou heparina de baixo peso molecular subcutânea em doses terapêuticas devem ser reintroduzidas. A varfarina deve ser prescrita 48 horas após o parto, obedecendo a dinâmica de transição em conjunto com a heparina até o valor de 2,0 da INR, quando é dada a alta hospitalar.

### 19.3. Parto e Puerpério

A programação do parto deve ser multidisciplinar a partir da 34ª semana de gestação. Considera-se o parto vaginal mais vantajoso porque está associado a menor perda sanguínea e menores riscos trombótico e infeccioso. As técnicas anestésicas sequenciais, com bloqueio do neuroeixo, apresentam vantagens hemodinâmicas porque permitem a forma gradual do bloqueio simpático. Os casos de indicação materna de cesárea geralmente requerem anestesia geral (Quadro 87).

**Quadro 87:** Recomendações para via de parto e anestesia em portadoras de doença valvar

Parto vaginal e anestesia peridural/raquidiana são preferenciais para doença valvar de risco baixo e intermediário Parto cesárea deve ser considerado Doença Valvar de Alto Risco (lesões obstrutivas graves) Doenças de aorta torácica ascendente Parto na vigência de anticoagulação História de dissecção de aorta Profilaxia antibiótica na ocasião do parto não é mais rotina. Contudo, pode ser considerada em próteses valvares ou história de endocardite infecciosa: Ampicilina 2,0 g endovenoso + Gentamicina 1,5 mg/kg/dia IM uma hora antes do parto Sem restrições à amamentação

### 19.4. Contracepção

A seleção dos métodos de contracepção para mulheres com doenças valvares exige a parceria multidisciplinar – ginecologista e cardiologista – para busca da segurança, eficácia, tolerância e fácil acesso. Nesse sentido, a orientação para a prescrição deve se apoiar nos Critérios de Elegilibidade dos Contraceptivos que classifica os anticoncepcionais em quatro categorias de risco e no índice de Pearl que calcula a eficácia do método considerando o número de gravidez em 100 mulheres no primeiro ano do uso do método.^220^^,^^221^ Para pacientes portadoras de doença valvar a tendência atual é se indicar os métodos que contenham progesterona isolada ou os combinados de progesterona com estrógeno natural na forma injetável/mensal, porque são seguros, eficazes e de fácil acesso (Quadro 88). Embora os dispositivos intrauterinos estejam classificados na categoria 2 eles não têm sido indicados em portadoras de doenças valvares mesmo que não complicada, pelo presumível risco inerente de EI.

**Quadro 88:** Critérios médicos de elegibilidade (modificado)* e índice de eficácia para o uso de contraceptivo em portadoras de doença valvar^220^^,^^221^

Contraceptivos disponíveis	AHCO	Injetável mensal	Pílula de progesterona	Injetável de progesterona	Implante de progesterona	DIU de cobre	DIU com levonorgestrel
Doença valvar							
Não complicada	2	1	1	1	1	3/4	3/4
Fatores complicadores	4	4	1	1	1	4	4
Eficácia	8	3	3	3	0,05	0,8	0,1

* Fatores complicador: Eficácia (Índice de Pearl) calculada em número de gravidez em 100 mulheres com uso habitual do método. Critérios de Elegibilidade: categoria 1- não há restrição quanto ao uso do método; categoria 2- vantagens de usar o método geralmente superam os riscos teóricos ou comprovados; categoria 3- os riscos teóricos ou comprovados geralmente superam as vantagens de usar o método e categoria 4- condição que representa um risco de saúde inaceitável se o método contraceptivo for usado. AHCO: anticoncepcional hormonal combinado oral; DIU: dispositivo intrauterino.

## Lista de Abreviaturas:

βHCG: gonadotrofina coriônica beta
ACC/AHA: American College of Cardiology/American Heart Association
AD: átrio direito
AE: átrio esquerdo
AOE: área efetiva do orifício
AAS: ácido acetilsalicílico
AHCO: anticoncepcional hormonal combinado oral
AVA: área valvar aórtica
AVM: área valvar mitral
BNP: peptídeo natriurético cerebral
CF: classe funcional
DDVE: diâmetro diastólico do ventrículo esquerdo
DIU: dispositivo intrauterino
DOACs: anticoagulantes orais diretos
DSVE: diâmetro sistólico do ventrículo esquerdo
EAo: estenose aórtica
ECG: eletrocardiograma
EI: endocardite infecciosa
EM: estenose mitral
ERO: área efetiva do orifício regurgitante
ESC/EACTS: European Society of Cardiology/European Association for CardioThoracic Surgery
ET: estenose tricúspide
FA: fibrilação atrial
FEVE: fração de ejeção do ventrículo esquerdo
FR: febre reumática
HP: hipertensão pulmonar
IAo: insuficiência aórtica
IC: insuficiência cardíaca
IM: insuficiência mitral
INR: razão normalizada internacional
IT: insuficiência tricúspide
NYHA: New York Heart Association
PHT: pressure half time
PSAP: pressão sistólica da artéria pulmonar
rTPA: recombinant tissue plasminogen activator.
SBC: Sociedade Brasileira de Cardiologia
STS: Society of Thoracic Surgeons
TAVI: implante transcateter de bioprótese aórtica
TTPa: tempo de tromboplastina parcial ativada
VE: ventrículo esquerdo
VKA: antagonistas da vitamina K
VMCB: valvoplastia mitral por cateter-balão
VTCB: valvoplastia tricúspide por cateter-balão
