# Supplementary material for: Atualização das Diretrizes Brasileiras de Valvopatias – 2020
Source: Arq Bras Cardiol. 2020 Oct 13;115(4):720–75. [Article in Portuguese] doi: 10.36660/abc.20201047 (PMC8386977; doi:10.36660/abc.20201047)
Supplement: Translation

# Translation: Update of the Brazilian Guidelines for Valvular Heart Disease – 2020

**Authors:** Flavio Tarasoutchi, Marcelo Westerlund Montera, Auristela Isabel de Oliveira Ramos, Roney Orismar Sampaio, Vitor Emer Egypto Rosa, Tarso Augusto Duenhas Accorsi, Antonio de Santis, João Ricardo Cordeiro Fernandes, Lucas José Tachotti Pires, Guilherme S. Spina, Marcelo Luiz Campos Vieira, Paulo de Lara Lavitola, Walkiria Samuel Ávila, Milena Ribeiro Paixão, Tiago Bignoto, Dorival Júlio Della Togna, Evandro Tinoco Mesquita, William Antônio de Magalhães Esteves, Fernando Atik, Alexandre Siciliano Colafranceschi, Valdir Ambrósio Moises, Alberto Takeshi Kiyose, Pablo M. A. Pomerantzeff, Pedro A. Lemos, Fabio Sandoli de Brito, Clara Weksler, Carlos Manuel de Almeida Brandão, Robinson Poffo, Ricardo Simões, Salvador Rassi, Paulo Ernesto Leães, Ricardo Mourilhe-Rocha, José Luiz Barros Pena, Fabio Biscegli Jatene, Márcia de Melo Barbosa, Alexandre Abizaid, Henrique Barbosa Ribeiro, Fernando Bacal, Carlos Eduardo Rochitte, José Honório de Almeida Palma da Fonseca, Samira Kaissar Nasr Ghorayeb, Marcelo Antonio Cartaxo Queiroga Lopes, Salvador Vicente Spina, Ricardo H. Pignatelli, José Francisco Kerr Saraiva

**Affiliations:** 1 São PauloSP Brazil Instituto do Coração (Incor) do Hospital de Clínica da Faculdade de Medicina da Universidade de São Paulo (HCFMUSP), São Paulo, SP – Brazil; 2 Rio de JaneiroRJ Brazil Hospital Pró-Cardíaco, Rio de Janeiro, RJ – Brazil; 3 São PauloSP Brazil Instituto Dante Pazzanese de Cardiologia, São Paulo, SP – Brazil; 4 Rio de JaneiroRJ Brazil Universidade Federal Fluminense, Rio de Janeiro, RJ – Brazil; 5 Belo HorizonteMG Brazil Universidade Federal de Minas Gerais, Belo Horizonte, MG – Brazil; 6 São PauloSP Brazil Fundação Universitária de Cardiologia (FUC), São Paulo, SP – Brazil; 7 São PauloSP Brazil Universidade Federal de São Paulo (UNIFESP), São Paulo, SP – Brazil; 8 São PauloSP Brazil Faculdade de Medicina da Universidade de São Paulo (FMUSP), São Paulo, SP – Brazil; 9 São PauloSP Brazil Hospital Israelita Albert Einstein, São Paulo, SP – Brazil; 10 São PauloSP Brazil Fundação Zerbini, São Paulo, SP – Brazil; 11 Rio de JaneiroRJ Brazil Instituto Nacional de Cardiologia, Rio de Janeiro, RJ – Brazil; 12 Belo HorizonteMG Brazil Faculdade Ciências Médicas de Minas Gerais, Belo Horizonte, MG – Brazil; 13 GoiâniaGO Brazil Universidade Federal de Goiás, Goiânia, GO – Brazil; 14 Porto AlegreRS Brazil Santa Casa de Misericórdia de Porto Alegre, Porto Alegre, RS – Brazil; 15 Rio de JaneiroRJ Brazil Hospital Universitário Pedro Ernesto, Rio de Janeiro, RJ – Brazil; 16 Belo HorizonteMG Brazil Hospital Felício Rocho, Belo Horizonte, MG – Brazil; 17 Belo HorizonteMG Brazil Hospital Socor, Belo Horizonte, MG – Brazil; 18 João PessoaPB Brazil Hospital Alberto Urquiza Wanderley, João Pessoa, PB – Brazil; 19 Houston USA Texas Children’s Hospital, Houston – USA; 20 CampinasSP Brazil Sociedade Campineira de Educação e Instrução Mantenedora da Pontifícia Universidade Católica de Campinas, Campinas, SP – Brazil

## 1. Introduction

There are currently a wide modalities of interventional strategies - both transcatheter and surgical - which can be indicated for patients with valvular heart diseases (VHD), with the objective of reducing the morbidity and mortality. The correct timing for indication and the type of interventional treatment are linked to the precise anatomical and functional diagnosis of the VHD, and comprehensive global evaluation of the patient. The 2020 Update of the Brazilian Guidelines for VHD, in addition to compiling scientific evidence and expert opinion, continues with the ideal of being useful in supporting decision making for patients with VHD, and has three unique characteristics, namely:

- Maintenance of the innovative flowcharts proposed in the 2017 edition, with sequential steps guiding anatomical, etiological, and functional diagnosis, defining conduct aligned with best practices and rational use of resources (Figure 1);
- The increase of the recommendations number in the attempt to contemplate the diverse possibilities in view of increasing complexity of patients;
- Comparison of the recommendations of these guidelines with the leading international ones, the American College of Cardiology/American Heart Association (ACC/AHA) 2017 and the European Society of Cardiology/European Association for Cardiothoracic Surgery (ESC/EACTS) 2017 Guidelines, allowing for individualization of the Brazilian population.^1^^,^^2^

**Figure 1:** Flowchart showing steps of anatomical, etiological, and functional diagnosis, in addition to the intervention decision making.

This 2020 edition considers the evaluation process for patients with non-severe VHD, and it emphasizes the need to weigh the possibility of transcatheter intervention in elderly patients, regardless of surgical risk, in addition patients with native or prosthetic valves with high surgical risk. Notwithstanding great advances and increased availability of imaging exams, these guidelines maintain the recommendation of detailed clinical evaluation, which continues to be indispensable to diagnosis, decisions making, and the doctor-patient relationship.

Bellow, the 5 recommended steps:

- First step: verify whether the VHD is anatomically severe; if so, proceed to the second step. In the event of non-severe valvular disease, investigate differential diagnoses in symptomatic patients and monitor evolution in asymptomatic patients;
- Second step: evaluate etiology, including clinical and past history, beside complementary exams;
- Third step: evaluate symptoms; this is fundamental to intervention decision making. Pharmacological treatment is indicated to alleviate symptoms until interventional takes place;
- Fourth step: evaluation of anatomical and/or functional prognostic factors (especially pulmonary hypertension [PH], ventricular remodeling, systolic dysfunction, aneurysmatic dilation of the aorta, and atrial fibrillation [AF]). This can be decisive regarding intervention in asymptomatic patients;
- Fifth step: type of intervention. The procedure can be surgical or transcatheter, with individualized indication depending on operative risks, comorbidities, and the Heart Team’s decision.

## 2. The Heart Team

The Heart Team is a group of different professionals with experience in valve diseases who share the decision regarding the most appropriate treatment for a given patient. Given the wide variety of interventional strategies available, the Heart Team is fundamental to risk-benefit and cost-effectiveness analyses and decision making. The Heart Team comprises diverse cardiological subspecialties; the members will play different fundamental roles during each step of care, from the clinical cardiologist, who is responsible for patient selection and indication, besides pre- and post-intervention follow-up, to the cardiac surgeon and the hemodynamicist, who will be responsible to perform the procedures indicated by the Heart Team. The radiologist will also be important to data analysis in order to evaluate the technical possibility of each intervention, and the echocardiographer, in addition to evaluating preoperative data, will also monitor the procedure, collaborating for better results.^1^^,^^2^

## 3. Operative Risk Evaluation

Indication of intervention for patients with VHD should always be based on the benefits and risks of the proposed procedure. For this purpose, we utilize online scores, including the EuroSCORE II (http://www.euroscore.org/calc.html) and the Society of Thoracic Surgeons (STS) score (http://riskcalc.sts.org/stswebriskcalc/#/calculate), which have been validated in different populations regarding their predictive ability of 30-day mortality. Patients with STS < 4% are conventionally considered at low surgical risk, while those with scores between 4% and 8% have intermediate risk, and those with scores > 8% have high risk. Rearding the EuroSCORE II, patients are considered low risk when it is lower than 4%, and, if the score is > 4%, they are considered at high surgical risk. In the event of a discrepancy between the two scores, we must use the one whose estimated higher risk.^3^^–^^8^

It is important to point that both scores omit some factors related to prognostic outcomes, such as frailty and specific contraindications to procedures, such as porcelain aorta. Furthermore, risk evaluation does not substitute the individual clinical evaluation, and the decision regarding intervention should always be shared with patients and their families.

## 4. Frailty

Frailty is an entity that denotes a state of vulnerability in elderly patients, associated with physical weakness and low physiological reserve. It is extremely relevant to individualized evaluation, mainly due to the following two factors:

– It is a predictor of events, such as mortality, length of hospital stay, and functional decline, after surgical or transcatheter intervention;
– It is not taken into consideration in conventional risk scores.

Several scores and tools are available for evaluating and quantifying frailty, through measurement of data related to functional status, instrumental daily activities, nutrition, cognition, independence for activities, and other factors. It is important that evaluation of frailty is not only subjective (“eyeball test”), but rather a set of clinical impression associated with different objective measurements and scores.^9^^–^^14^

## 5. Mitral Stenosis

Physical examination is the first resource applied for anatomical evaluation of mitral stenosis (MS). Patients with mild to moderate MS may already present an opening snap as well as a decrescendo rumbling diastolic murmur in the mitral area, starting immediately after the click. In patients in sinus rhythm, the murmur shows presystolic reinforcement in the end of diastole. In patients with severe MS, however, these clinical changes become more evident, as electrocardiographic and radiologic changes get evident. The characteristics present in patients with severe MS are shown in Table 1.

**Table 1:** Step 1: Diagnosis of severe mitral stenosis^15^

	Characteristics of severe mitral stenosis
Physical examination	*Facies mitralis* Early opening snap Hyperphonetic first heart sound Hyperphonetic second heart sound Rumbling diastolic murmur, with presystolic reinforcement for patients in sinus rhythm Signs of pulmonary congestion and right heart failure Presence of TR
Electrocardiogram	LA enlargement Right chambers overload AF
Chest radiography	Normal cardiothoracic index Signs of enlarged LA: Elevated left main bronchus (“ballerina sign”) Double atrial contour on the right 4^th^ arch in the cardiac silhouette on the left Signs of pulmonary congestion
Echocardiogram	MVA < 1.5 cm^2^ Average diastolic transmitral gradient ≥ 10 mmHg Resting SPAP ≥ 50 mmHg SPAP ≥ 60 mmHg during exertion
Hemodynamic study	Indicated in the event of discordance between clinical and echocardiographic findings Diastolic transmitral gradient ≥ 10 mmHg (spontaneous or after atropine and volume) SPAP ≥ 50 mmHg

AF: atrial fibrillation; LA: left atrium; LV: left ventricle; MVA: mitral valve area; SPAP: systolic pulmonary artery pressure; TR: tricuspid regurgitation.

Echocardiography is the main complementary exam for mitral valve anatomical evaluation, and it is fundamental for defining the severity of VHD, hemodynamic repercussions, and parameters regarding intervention success, with evaluation of the components of the valve (valve annulus, valve cusps, and subvalvular apparatus).

The echocardiographic parameters of severe MS are mitral valve area (MVA), which may be measured by planimetry, pressure half time (PHT), or the continuity equation, and transmitral diastolic gradient.^15^

From the epidemiological point of view (Table 2), the main etiology of MS continues to be rheumatic fever (RF), which remains prevalent in developing countries, including Brazil. In these countries, rheumatic valve disease maintains an estimated prevalence of 1 to 7 per 1,000 children in clinical studies; this number is up to 10 times higher when echocardiography is used for population screening. Regarding developed countries, statistics indicate that MS is responsible for 9% of all VHD in Europe, and 0.1% in the United States. In these countries, cases occur predominantly in elderly patients and young immigrants from developing countries.^16^^–^^18^

**Table 2:** Step 2: Evaluation of etiology of severe mitral stenosis ^16^^,^^17^

	Etiological characteristics
Rheumatic fever	> 90% of cases in developing countries Symptoms between the third and fourth decades of life Commissural fusion, thickening of cusps Compromised subvalvular apparatus Dome opening of the anterior cusp and reduced mobility of the posterior cusp Mitral-aortic involvement
Degenerative	12% to 26% of cases in developed countries More common in elderly patients May reach 60% of cases in patients over 80 years Calcification of the mitral valve annulus Absence of commissural fusion Related to aortic and coronary calcification
Rare causes	Congenital Rheumatologic diseases (lupus or rheumatoid arthritis) Medication (methysergide or anorexigenic drugs) Carcinoid syndrome Fabry disease Actinic injury – post-radiotherapy

In addition to the rheumatic etiology, there is a proportional increase in the number of patients with mitral annulus calcification (MAC), which may extend to the base of the valve leaflets, leading to restricted cusp movement and restriction of atrial emptying. The estimated prevalence of MAC is around 10% of the elderly population and approximately 1% to 2% of these patients develop MS.^19^

Other rare causes of MS include: rheumatologic diseases (systemic lupus erythematosus or rheumatoid arthritis), deposit diseases (such as Fabry disease), Whipple disease, therapy with methysergide or anorexigenic drugs, carcinoid syndrome, or congenital anatomical abnormalities of the mitral valve, such as parachute mitral valve or mitral valve hypoplasia.

In patients with severe MS, it is necessary to pay attention to the symptoms (Table 3), the most common being dyspnea (New York Heart Association [NYHA] functional class [FC] II to IV). In particular, dyspnea may appear in situations that lead to increased pulmonary capillary pressure (physical exertion, pregnancy, or AF). Over time, it may also appear at rest, even with orthopnea. Other symptoms that may appear are palpitations, hemoptysis, dysphonia, dysphagia, cough, and embolic events.

**Table 3:** Evaluation of severe mitral stenosis symptoms

	Symptoms
Dyspnea (NYHA FC II to IV)	Main symptom Initially with situations that increase pulmonary capillary pressure (physical exertion, atrial fibrillation, or pregnancy) Resting dyspnea and nocturnal paroxysmal dyspnea May be accompanied by palpitations, hemoptysis, dysphonia, dysphagia, cough May be accompanied by embolic events (cerebral, mesenteric, or extremities)

FC: functional class; NYHA: New York Heart Association.

In parallel to the evaluation of symptoms, possible prognostic factors should be investigated (Table 4). With respect to severe MS, relevant signals are the presence of significant PH (systolic pulmonary artery pressure – SPAP above 50 mmHg when resting or above 60 mmHg during exertion) or recent onset AF (triggered in the recent months).

**Table 4:** Step 4: Evaluation of severe mitral stenosis prognostic factors

	Prognostic Factors
Pulmonary hypertension	Resting SPAP ≥ 50 mmHg SPAP ≥ 60 mmHg during exertion (exercise test or echocardiography with pharmacological stress)
Recent onset AF	Relation to LA enlargement Maintain INR between 2.0 and 3.0

**AF: atrial fibrillation; LA: left atrium; INR: international normalized ratio; SPAP: systolic pulmonary artery pressure.:**

Types of intervention and their indications are described in Tables 5 and 6 and Figure 2. Percutaneous balloon mitral valvuloplasty (PBMV) remains the treatment of choice for patients with MS of rheumatic etiology, wherein calcification and commissural fusion are predominant. There is need for favorable valve anatomy (as evaluated by the Wilkins-Block score [Table 7]) and no procedure contraindications (moderate to severe mitral regurgitation [MR] and left atrium [LA] thrombus). The Wilkins-Block score consists of echocardiographic evaluation of the mitral valve, with emphasis on description of structural aspects. The following four parameters are taken into consideration: leaflet mobility, valve thickening, degree of cuspid calcification, and involvement of the subvalvular apparatus. Values from 1 to 4 points for each item result in scores ranging from 4 to 16 points. Patients with Wilkins-Block score less than or equal to 8 are candidates for PBMV, since there are no contraindications. Surgical treatment of the mitral valve is the treatment of choice for patients with unfavorable anatomy or contraindications for PBMV in the presence of symptoms (NYHA FC III or IV) or prognostic factors. Surgery may consist of mitral commissurotomy or, in cases of very significant valve impairment, valve replacement with a biological or mechanical prosthesis.^20^^,^^21^

**Table 5:** Step 5: Type of mitral stenosis intervention^15^^,^^17^^,^^20^^–^^25^

Type	Considerations
Percutaneous balloon mitral valvuloplasty	Treatment of choice in rheumatic etiology Indications: Symptoms (NYHA FC II to IV) and/or prognostic factors Wilkins-Block echocardiographic score ≤ 8 * (subvalvular apparatus and calcification ≤ 2) In pregnant women or patients with high surgical risk, consider if: echocardiographic score 9 to 10 (subvalvular apparatus and calcification ≤ 2) Contraindications: – LA thrombus – Moderate or severe MR – Recent embolic phenomenon
Surgical treatment (commissurotomy/valve replacement)	Rheumatic MS with NYHA FC III to IV and contraindications to PBMV Rheumatic MS with prognostic factors, not eligible for PBMV Degenerative MS, refractory to medical treatment
Transcatheter mitral valve implantation (valve-in-MAC)	Degenerative MS, refractory to medical treatment, with contraindication or high surgical risk (currently under study)

* Individualize in cases with echocardiographic scores 9 to 10. Patients with calcification and subvalvular apparatus scores below 3 have higher rates of successful PBMV. PBMV: percutaneous balloon mitral valvuloplasty; FC: functional class; LA: left atrium; MAC: mitral annulus calcification; MR: mitral regurgitation; MS: mitral stenosis.

**Table 6:** Mitral stenosis: Recommendations^1^^,^^2^^,^^15^^,^^17^^,^^20^^–^^25^

Intervention	Clinical condition	SBC	AHA	ESC
Percutaneous balloon mitral valvuloplasty	Rheumatic MS, NYHA FC II to IV, in the absence of contraindications	I A	I A	I B
	Asymptomatic rheumatic MS, with prognostic factors, in the absence of contraindications	I C	IIb C (if AF)	IIa C (if high thromboembolic risk or risk of hemodynamic deterioration)
Surgical treatment (commissurotomy/valve replacement)	• Rheumatic MS, NYHA FC III to IV, with contraindications to PBMV	I B	I B	I C
	Asymptomatic rheumatic MS with prognostic factors, not eligible for PBMV	IIa C	IIb C (Recurrent embolism)	–
	Degenerative MS refractory to medical treatment	IIb C*	–	–
	Asymptomatic rheumatic MS with other concomitant heart surgery	I C	I C	–
Transcatheter mitral valve implantation (valve-in-MAC)	Degenerative MS refractory to medical treatment, with contraindication or high surgical risk	IIb C*	–	–

* Consider evaluation of the Heart Team. AHA: American Heart Association; PBMV: percutaneous balloon mitral valvuloplasty; ESC: European Society of Cardiology; FC: functional class; MAC: mitral annulus calcification; MS: mitral stenosis; SBC: Sociedade Brasileira de Cardiologia (Brazilian Society of Cardiology).

**Table 7:** Wilkins-Block echocardiographic score

Leaflet mobility: - High valve mobility with restriction only in the extremities of the leaflets - The medial and basal regions show normal mobility - The valve continues moving forward in diastole, mainly at the base - Minimal or no movement of the leaflets in diastole
Subvalvular thickening: - Minimal subvalvular thickening exactly below the mitral leaflets - Chordal thickening extending for more than a third of length - Thickening extending to the distal third of the chordae - Extensive thickening and shortening of all structures of the chordae extending to the papillary muscles
Leaflet thickness: - Thickening of the leaflets, with thickness close to normal (4 – 5 mm) - Normal medial layers, considerable thickening of margins (5 – 8 mm) - Thickening extending throughout all the layer (5 – 8 mm) - Considerable thickening of the entire tissue layer (> 8 – 10 mm)
Valve calcification: - Single area of increased brightness - Minimal areas of brightness confined to the leaflet margins - Brightness extending inside the middle portion of the leaflets - Extensive brightness, beyond the limits of the leaflets

**Figure 2:** Flowchart for decision making in mitral stenosis. AF: atrial fibrillation; PBMV: percutaneous balloon mitral valvuloplasty.

For patients with degenerative MS, on the other hand, PBMV is not a therapeutic option, as there is no commissural fusion or calcification, but rather valve annulus calcification. Furthermore, in these patients, who are usually elderly and often have multiple comorbidities, surgical risk is significantly higher. The surgical procedure is technical difficulty and is more likely to have complications, including atrioventricular disjunction, circumflex artery injury, and ventricular wall bleeding. The initial treatment of choice is, thus, clinical: heart rate control with betablockers, calcium channel blocker or ivabradine (for patients in sinus rhythm who have not tolerated previous medications), associated with diuretics.^22^ If this strategy works, patients may continue with medical treatment, without indication for further interventions. For patients who are refractory to clinical treatment, however, it is necessary to consider the possibility of surgical intervention, in cases with low to moderate risk, or eventual transcatheter implantation of a mitral prosthesis. In these cases, transcatheter implantation uses the MAC to support the valve prosthesis, in a procedure routinely referred to as valve-in-MAC. There is still limited experience with this procedure, which is most frequently performed in clinical studies via the transeptal or transapical route. It still has a high rate of complications, including paravalvular leak, left ventricle (LV) outflow tract obstruction, and prosthesis embolization, and the mortality rate may reach 25% in 30 days and 54% in 12 months. Further studies are needed in order to broaden its indications.^23^^–^^25^

Clinical follow-up of patients, as long as they present non-severe VHD, consists of periodic consultations and echocardiographic reevaluation (Table 8). In patients with non-severe MS, reevaluation may be performed on a yearly basis. Patients with valve area ≥ 1.5 cm^2^ are not normally expected to develop symptoms or prognostic factors. In the event that these changes occur, before the patient develops anatomically severe VHD, it is imperative to consider the possibility that other differential diagnoses are present. Patients with severe MS, on the other hand, should be reevaluated at shorter intervals, usually every 6 to 12 months.

**Table 8:** Mitral stenosis: Individualized follow-up^1^^,^^2^

Mitral stenosis	Follow-up	SBC	AHA	ESC
Severe and asymptomatic, without prognostic factors	Clinical and echocardiographic evaluation	Every 6 to 12 months	Every 12 months	Every 12 months
	Concomitant surgical intervention in patients who will undergo other cardiac surgical procedure (coronary revascularization, ascending aorta, or other valve procedures)	I C	IIb C	–
Non-severe (MVA > 1.5 cm² and mean transmitral gradient < 5 mmHg)	Clinical and echocardiographic reevaluation	Every 1 years	Every 3 to 5 years	Every 2 to 3 years

AHA: American Heart Association; ESC: European Society of Cardiology; LA: left atrium; LV: left ventricle; MVA: mitral valve area; SBC: Sociedade Brasileira de Cardiologia (Brazilian Society of Cardiology).

## 6. Primary Chronic Mitral Regurgitation

For the clinical decision making in primary chronic MR, it is recommended that the 5 steps of the flowchart for treating VHD are followed, as detailed below and subsequently summarized in Figure 3.

**Figure 3:** Flowchart for decision making in primary chronic mitral regurgitation. AF: atrial fibrillation; LVEF: left ventricular ejection fraction; LVSD: left ventricular systolic diameter; SPAP: systolic pulmonary artery pressure.

In addition to confirming the presence of VHD, transthoracic echocardiogram is the main exam used to define the anatomical severity of MR. Diverse parameters may be used for this quantification; detailed, thorough examination is of fundamental importance (Table 9).

**Table 9:** Step 1: Diagnosis of severe primary mitral regurgitation^26-32^

	Characteristics of severe primary mitral regurgitation
Physical examination	Apex beat shifted to the left and downward Hypophonetic S1 (frequently audible in patients with rheumatic MR and MR due to prolapse, and loss of intensity may be considered a marker of severity of ventricular dysfunction, chordal rupture, among others) Hyperphonetic S2 Regurgitative systolic murmur ≥ +++/6+ Clinical signs of right heart failure
Electrocardiogram	Left ventricular hypertrophy Left atrial enlargement Arial or ventricular arrhythmias (PVCs, tachycardia) and AF
Chest radiography	Enlarged cardiac silhouette with LV and LA dilation Signs of pulmonary congestion
Echocardiogram	Jet area ≥ 40% of LA area Regurgitant fraction ≥ 50% Regurgitant volume ≥ 60 mL/beat Vena contracta ≥ 0.7 cm Effective regurgitant orifice area (EROA) • ≥ 0.40 cm²
Hemodynamic study	Indicated in cases of disagreement between clinical and echocardiographic findings Left ventriculography (severe if > 3+) Evaluation of intracavitary pressures
Magnetic resonance	In cases of disagreement between clinical and echocardiographic findings or limited quality of echocardiographic image Confirmation of degree of MR before scheduled mitral valve intervention Degree of MR Evaluation of mitral annulus disjunction in the complex of myxomatous disease and mitral valve prolapse

AF: atrial fibrillation; LA: left atrium; LV: left ventricle; MR: mitral regurgitation; PVCs: premature ventricular contractions.

Patients with anatomically mild or moderate MR should continue with periodic clinical and echocardiographic follow-up, and there is no indication for intervention (medical or surgical) in order to interrupt the natural history of the valve disease. On the other hand, patients with severe MR should proceed as per the flowchart for specific evaluation, investigating symptoms that are secondary to the VHD and/or the presence of prognostic factors.

In patients with MR, it is necessary to define the etiology of the VHD, given that clinical follow-up and therapeutic planning (timing and type of intervention), when indicated, can be different according to the cause of MR (Table 10). In spite of advances in diagnostic tests, transthoracic echocardiogram remains the first and main exam indicated for anatomical quantification and etiological evaluation of patients with MR. ^33^^–^^35^

**Table 10:** Step 2: Evaluation of severe primary mitral regurgitation etiology^33^^–^^35^

	Etiological characteristics
Rheumatic	Most prevalent cause in Brazil Thickening with cusp retraction Commissural involvement Mitral-aortic involvement Frequent in young adults
Mitral valve prolapse and associated diseases (Barlow syndrome)	Second most frequent cause in Brazil Cusp protrusion into the LA ≥ 2 mm More frequent in middle-aged and elderly populations
Other causes	Infective endocarditis Marfan syndrome Systemic lupus erythematosus Traumatic lesions Congenital deformities

LA: left atrium.

The main symptom in patients with anatomically severe MR is dyspnea, which should be taken in account, even if it does not limit routine activities (NYHA FC II). If there are doubts regarding the presence of symptoms, an exercise test or cardiopulmonary test may be requested (Table 11). Once the presence of symptoms has been confirmed, and if they are secondary to MR, patients should be referred for valvular intervention, as described in Step 5 (Table 12).

**Table 11:** Step 3: Evaluation of severe primary mitral regurgitation symptoms

	Symptoms
Dyspnea (NYHA FC II-IV) and fatigue/weakness	Pulmonary congestion Initially with events that increase pulmonary capillary pressure (physical exertion, AF, pregnancy) Resting dyspnea and nocturnal paroxysmal dyspnea May be accompanied by palpitations, coughing, edema May be accompanied by embolic events

AF: atrial fibrillation; FC: functional class.

**Table 12:** Step 5: Type of severe primary mitral regurgitation intervention^39^^–^^52^

Type of intervention	Considerations
Mitral valve repair	Treatment of choice Rheumatic patients: less favorable results. Prolapse of the posterior cusp of the mitral valve (isolated P2): better results.
Mitral valve replacement	Indicated in cases where valve repair is not possible.
Percutaneous mitral valve repair	Reserved for high-risk patients or patients with surgical contraindication and refractory symptoms Degenerative MR due to prolapse Favorable anatomical conditions Indicated following decision by the Heart Team

MR: mitral regurgitation.

Patients with severe asymptomatic MR should be periodically reevaluated to determine the development of anatomical and/or functional changes secondary to valve disease (Table 13). The following prognostic factors are associated with MR: LV systolic dysfunction (left ventricular ejection fraction [LVEF] < 60%), LV dilation (left ventricular systolic diameter [LVSD] ≥ 40 mm), PH (SPAP ≥ 50 mmHg at rest or ≥ 60 mmHg during exertion), and new onset AF (recent months).^36^^–^^38^ Increased LA volume (especially ≥ 60 ml/m^2^) may be considered an anatomical prognostic factor in MR, and it should be taken into consideration for intervention decision, given that it is associated with worse prognosis. In addition, if there is a progressive decline in LVEF or progressive dilation of the LV on serial imaging tests, mitral valve intervention should be considered, even before the previously mentioned limits have been reached.

**Table 13:** Step 4: Evaluation of severe primary mitral regurgitation prognostic factors^36^^–^^38^

	Prognostic factors
Echocardiogram	LVEF ≤ 60% or progressive decline in LVEF (within normal range) Progressive remodeling (LVSD ≥ 40 mm) Resting SPAP ≥ 50 mmHg or ≥ 60 mmHg during exercise LA volume ≥ 60 ml/m²
Electrocardiogram	Recent onset AF (< 1 year)

AF: atrial fibrillation; LA: left atrium; LVEF: left ventricular ejection fraction; LVSD: left ventricular systolic diameter; SPAP: systolic pulmonary artery pressure.

After confirming the existence of anatomically severe MR, with a defined etiology, and, finally, verifying the presence of symptoms that are secondary to the VHD and/or the presence of prognostic factors, the patient should be referred for valvular intervention, if there is no contraindication (Table 13 and 14). In these cases, surgical mitral repair is the treatment of choice, provided that the etiology (especially prolapse) and the anatomy are favorable and that the procedure is performed in a qualified hospital with an experienced surgeon. Otherwise, surgical mitral valve replacement is indicated.^39^^–^^52^

**Table 14:** Primary mitral regurgitation: Recommendations^1^^,^^2^^,^^39^^–^^52^

Intervention	Clinical condition	SBC	AHA	ESC
Mitral valve repair (centers with experience)	**Rheumatic**			
	Symptomatic (NYHA FC ≥ II)	IIb C	IIb C	–
	Asymptomatic, with prognostic factors: – LVEF between 30% and 60% and/or LVSD ≥ 40 mm	IIb B	IIb B	–
	- SPAP ≥ 50 mmHg or AF	IIb B	–	–
	Rheumatic, asymptomatic MR without prognostic factors	III	–	–
	**Non-rheumatic**			
	NYHA FC ≥ II, with favorable anatom	I B	I B	I B
	Asymptomatic, with favorable anatomy and prognostic factors: – LVEF between 30% and 60% and/or LVSD ≥ 40 mm	I B	I B	I B (LVSD ≥ 45 mm)
	- SPAP ≥ 50 mmHg or AF	IIa B	IIa B	IIa B
	Asymptomatic MR due to prolapse, with favorable anatomy, without prognostic factors	IIa B	IIa B	IIa C (LA ≥ 60 ml/m² and sinus rhythm)
Mitral valve replacement	**Rheumatic**			
	Symptomatic (NYHA FC ≥ II)	I B	–	–
	Asymptomatic, with prognostic factors: – LVEF between 30% and 60% and/or LVSD ≥ 40 mm	I B	–	–
	- SPAP ≥ 50 mmHg or AF	IIa B	–	–
	Rheumatic, asymptomatic MR, without prognostic factors	III	–	–
	**Non-rheumatic**			
	NYHA FC ≥ II, with unfavorable anatomy for valve repair	I B	I B	I B
	Asymptomatic, with unfavorable anatomy for valve repair, and prognostic factors: – LVEF between 30% and 60% and LVSD ≥ 40 mm	I B	I B	I C (LVSD ≥ 45 mm)
	- SPAP ≥ 50 mmHg or AF	IIa C	IIa C	IIa B
	Asymptomatic MR due to prolapse, with unfavorable anatomy for valve repair, without prognostic factors	III	III	III
Percutaneous mitral valve repair	Non-rheumatic MR, with high risk or contraindication to surgery, with refractory symptoms	IIa B*	IIb B	IIb C

* In centers with a Heart Team. AF: atrial fibrillation; AHA: American Heart Association; ESC: European Society of Cardiology; FC: functional class; LVEF: left ventricular ejection fraction; LVSD: left ventricular systolic diameter; SBC: Sociedade Brasileira de Cardiologia (Brazilian Society of Cardiology); SPAP: systolic pulmonary artery pressure.

Indication of transcatheter interventions is restricted to patients with primary MR, and the decision should be made following discussion with the Heart Team. In the same manner, for patients with contraindication or high risk associated with conventional surgery, prior discussion with the Heart Team needs to take place for the best decision making.

When patients, notwithstanding the presence of anatomically severe MR, do not show symptoms or have prognostic factors, they should receive individualized follow-up, with biannual clinical follow-up and echocardiographic evaluation at maximum 1-year intervals (Table 15).

**Table 15:** Primary mitral regurgitation: Individualized follow-up^1^^,^^2^

Primary mitral regurgitation	Follow-up	SBC	AHA	ESC
Severe and asymptomatic, without prognostic factors	Clinical and echocardiographic reevaluation	Every 6 months to 1 year	Every 6 months to 1 year	Every 6 months
	Concomitant intervention in patients who will undergo another cardiac surgical procedure (coronary revascularization, ascending aorta, or other valve procedures)	I B	I B	–
Moderate (Jet area 20% – 40% of LA area, regurgitant fraction 30% – 49%, regurgitant volume 30 – 59 mL/beat, vena contracta 0.3 – 0.69 cm, EROA 0.2 – 0.39 cm²)	Clinical and echocardiographic reevaluation	Every 1 to 2 years	Every 1 to 2 years	Every 1 to 2 years
	Concomitant intervention in patients who will undergo another cardiac surgical procedure (coronary revascularization, ascending aorta, or other valve procedures)	IIa C	IIa C	–
Mild (Jet area < 20% of LA area, regurgitant fraction < 30%, regurgitant volume < 30 mL/beat, vena contracta < 0.3 cm, EROA < 0.2 – 0.39 cm²)	Clinical and echocardiographic reevaluation	Every 2 to 3 years	Every 3 to 5 years	–

AHA: American Heart Association; EROA: effective regurgitant orifice area; ESC: European Society of Cardiology; LA: left atrium; SBC: Sociedade Brasileira de Cardiologia (Brazilian Society of Cardiology).

On the other hand, patients with anatomically moderate MR should receive annual clinical evaluation and undergo echocardiogram every 2 years.

## 7. Secondary Mitral Regurgitation

Secondary MR results from ventricular changes (dysfunction and/or dilation), while the mitral valve leaflets and chordae are normal. In this context, additional LV overload occurs due to mitral regurgitation, culminating in worse prognosis. The main etiologies are coronary artery disease (ischemic MR) and dilated cardiomyopathy (annular dilation and/or poor positioning). For these reasons, the ideal treatment is controversial, given that valve correction is not curative. In general, intervention is indicated in patients who remain symptomatic, in spite of optimized medical treatment. Even so, the therapeutic decision must be individualized and, whenever possible, shared with the Heart Team.^53^

As physical examination for diagnosis of secondary MR is often poor, transthoracic echocardiogram is a fundamental test. There is evidence that lower limits of the regurgitant orifice area and the regurgitant volume are associated with worse prognosis, in comparison with primary MR. Nevertheless, for quantification of anatomical severity of secondary MR, the echocardiographic limits applied are the same as those for primary MR. In the event of disagreement between clinical and echocardiographic findings, hemodynamic study with left ventriculography or magnetic resonance may assist in definition (Table 16).^27^^–^^32^^,^^54^

**Table 16:** Step 1: Diagnosis of severe secondary mitral regurgitation^27^^–^^32^^,^^54^

	Characteristics of severe secondary mitral regurgitation
Physical examination	Hypophonetic or normophonetic S1 Protomesosystolic or holosystolic murmur, radiating to the axillary line
Electrocardiogram	Left ventricular hypertrophy Left atrial enlargement Signs suggestive of the underlying pathology
Chest radiography	Enlarged cardiac silhouette due to dilation of left chambers
Echocardiogram	Quantification of regurgitation*: – Regurgitant fraction ≥ 50% – Regurgitant volume ≥ 60 mL/beat – EROA ≥ 0.40 cm²
Hemodynamic study	Disagreement between clinical and echocardiographic findings Degree of MR on left ventriculography
Magnetic resonance	Disagreement between clinical and echocardiographic findings or limited quality of echocardiographic image Confirmation of the degree of MR before scheduled mitral valve intervention Degree of MR

* Consider the possibility of anatomically severe mitral regurgitation if EROA is between 0.3 and 0.4 cm ² when associated with severe systolic dysfunction. EROA: effective regurgitant orifice area; MR: mitral regurgitation.

Echocardiogram provides the main information required for establishing the etiology of secondary MR, especially regarding analysis of LV changes (Table 17). Coronary cineangiography, in turn, plays an important role in diagnosis of obstructive coronary artery disease, which may be the cause of MR.^53^

**Table 17:** Step 2: Evaluation of severe secondary mitral regurgitation etiology^53^

	Etiological characteristics
Ischemic	Segmental changes in contractility Inadequate arrangement of the papillary muscles or leaflets (tenting leaflet or with apical traction – tethering – and/or due to failed leaflet coaptation) Mitral annular dilation or deformity Evaluation of coronary arteries on coronary cineangiography Evaluation of viability on cardiac magnetic resonance
Dilated	Valve annulus dilation – ventricular dilation Ventricular systolic dysfunction Inadequate arrangement of the papillary muscles or leaflets (tenting leaflet or with apical traction – tethering – and/or due to failed leaflet coaptation) Ventricular dyssynchrony Altered atrioventricular mechanical coupling

Tests for myocardial viability evaluation (such as nuclear magnetic resonance) may be useful in patients with ischemic MR who are scheduled for myocardial revascularization.

The main symptom present in patients with secondary MR is dyspnea, which may result from LV dysfunction and/or associated mitral regurgitation (Table 18).

**Table 18:** Step 3: Evaluation of severe secondary mitral regurgitation symptoms

	Symptoms
Dyspnea and fatigue/weakness	Increased end diastolic pressure Pulmonary capillary congestion May be accompanied by palpitations, cough, ascites, edema, or chest pain May be accompanied by embolic events

Patients with important symptoms (NYHA FC III and IV) that persist in spite of optimized treatment for heart failure (including resynchronization therapy, when indicated) should be considered for intervention in an individualized manner.

There are no specific prognostic factors for patients with secondary MR, given that the origin of the problem lies in ventricular disease (Table 19). Nonetheless, in the event that LV dilation and/or dysfunction worsen, without any clear causal factor, the concomitant mitral valve disease may be considered responsible.^55^^,^^56^

**Table 19:** Step 4: Evaluation of severe secondary mitral regurgitatio nprognostic factors^55^^,^^56^

Prognostic factors	
Clinical and echocardiographic evaluation	Worsening of underlying conditions without other attributable causes (increased SPAP, increased ventricular diameters, or decreased LVEF) Symptoms refractory to optimized clinical treatment

LVEF: left ventricular ejection fraction; SPAP: systolic pulmonary artery pressure.

Indication of intervention for patients with secondary MR is controversial (Tables 20 e 21). In patients with ischemic MR who are candidates for myocardial revascularization surgery, simultaneous approach to the mitral valve disease should be considered. On the other hand, in patients who are not indicated for revascularization, isolated surgical approach to MR is associated with high mortality and high rates of MR recurrence, and there is no evidence of its benefit in terms of survival.^53^^,^^57^^–^^66^

**Table 20:** Step 5: Type of severe secondary mitral regurgitatio n intervention^53^^,^^57^^–^^72^

Type	Considerations
Surgery (valve repair or replacement)	Valve replacement or repair + myocardial revascularization, when indicated
Percutaneous mitral valve repair	May be considered after evaluation by the Heart Team, especially in patients with LVEF ≥ 20% and LVSD < 70 mm

LVEF: left ventricular ejection fraction; LVSD: left ventricular systolic diameter.

**Table 21:** Secondary mitral regurgitation: Recommendations^1^^,^^2^^,^^53^^,^^57^^–^^72^

Intervention	Clinical condition	SBC	AHA	ESC
Mitral valve replacement or repair	**Ischemic**			
	Symptomatic (NYHA FC ≥ III) Associated revascularization	IIb B	IIb B	IIb C
		IIa B	IIa B	I C (LVEF > 30%) IIa C (LVEF < 30%)
	**Dilated**			
	Symptomatic (NYHA FC ≥ III)	IIb B	IIb B	IIb C
Percutaneous mitral valve repair	**Ischemic**			
	Refractory symptoms (NYHA FC ≥ III), with high risk or contraindication to surgery	IIa B	–	IIb C (LVEF < 30%)
	**Dilated**			
	Refractory symptoms (NYHA FC ≥ III) with high risk or contraindication to surgery	IIa B	–	IIb C (LVEF < 30%)

AHA: American Heart Association; ESC: European Society of Cardiology; FC: functional class; LVEF: left ventricular ejection fraction; SBC: Sociedade Brasileira de Cardiologia (Brazilian Society of Cardiology).

In patients with MR secondary to dilated cardiomyopathy, indication of intervention in mitral valve disease is even more restricted. While isolated mitral valve surgery has not demonstrated a benefit in this scenario, new evidences have shown a benefit to transcatheter intervention in patients with secondary MR and LVEF ≥ 20%, who remained symptomatic in spite of optimized clinical treatment, provided that the procedure is not indicated in more advanced phases of the natural history of VHD. ^67^^–^^72^

For more appropriate indication and more thorough approach, cases of secondary MI should be discussed with the Heart Team before the decision is made (Figure 4).

**Figure 4:** Flowchart for decision making in secondary mitral regurgitation.FC: functional class.

## 8. Aortic Stenosis

Aortic stenosis (AS) shows a growing prevalence due to increased life expectancy and consequent aging of the Brazilian population. The most common cause of AS is aortic calcification/degeneration, which mainly affects elderly patients. Transcatheter treatment has become an alternative to surgical valve replacement, not only in frail and high-risk patients, but also in patients with intermediate or low risk. Therefore, Heart Team is becoming increasingly important and necessary in decision making regarding intervention in these patients.73

According to current evidence and following the recommendations of the 2017 Brazilian guidelines, the first step for evaluating patients with AS is to define the VHD severity (Table 22). To date, only patients with anatomically severe AS benefit from intervention. Severe AS is defined in echocardiography as an aortic valve area (AVA) ≤ 1.0 cm² and/or indexed AVA ≤ 0.6 cm²/m² in the presence of mean transaortic gradient ≥ 40 mmHg or maximum aortic jet velocity ≥ 4.0 m/s. Patients with low-flow, low-gradient AS (AVA ≤ 1.0 cm² and mean transaortic gradient < 40 mmHg), once anatomical severity has been confirmed, may also undergo intervention. In cases with low-flow, low-gradient AS and preserved LVEF, it is necessary to measure aortic calcium score (severe AS if over 1,300 AU for women and over 2,000 AU for men).^74^^–^^82^ In patients with low-flow, low-gradient AS and low LVEF, dobutamine stress echocardiogram is indicated. Severe AS is defined when, in the presence of contractile reserve, AVA remains reduced,.^83^^–^^86^ In the absence of contractile reserve, it is also necessary to measure valve calcium score in order to define anatomical severity.^74^^–^^78^^,^^87^ Patients with no contractile reserve also benefit from surgical or transcatheter intervention.

**Table 22:** Step 1: Diagnosis of severe aortic stenosis^74^^–^^87^

	Characteristics of severe aortic stenosis
Physical examination	Pulsus parvus et tardus Ejective systolic murmur with telesystolic peak Hypophonetic second heart sound Hypophonetic first heart sound Gallavardin phenomenon Paradoxical double second heart sound or single second heart sound
Electrocardiogram	Left chamber overload Altered ventricular repolarization (strain pattern)
Chest radiography	Cardiothoracic index may be normal Signs of pulmonary congestion
Echocardiogram	AVA ≤ 1.0 cm^2^ Indexed AVA ≤ 0.6 cm^2^/m^2^ Mean transaortic gradient ≥ 40 mmHg Maximum aortic jet velocity ≥ 4.0 m/s Flow rate ratio between LV outflow tract and aortic valve < 0.25
Dobutamine stress echocardiogram	Indicated for evaluation of anatomical severity in patients with low-flow, low-gradient AS, with low LVEF, defined as AVA ≤ 1.0 cm^2^, LVEF < 50% and mean transaortic gradient < 40 mmHg* In the presence of contractile reserve (increase of ≥ 20% in stroke volume and/or increase of > 10 mmHg in mean transaortic gradient), patients with reduction or preservation in peak AVA during stress have severe AS (increase of up to 0.2 cm^2^ in AVA is accepted as a criterion of preserved AVA). Patients with increasing in AVA ≥ 0.3 cm² are defined as moderate AS (pseudo-severe AS) In the absence of contractile reserve, it is necessary to corroborate anatomical severity with the aortic calcium score
Multidetector chest computed tomography	Aortic valve calcium score above 1,300 AU for women and 2,000 AU for men confirms severe AS
Hemodynamic study	Transaortic gradient (peak) ≥ 50 mmHg
Special situation	Low-flow, low-gradient AS with preserved LVEF (“paradoxical”), defined as: AVA ≤ 1.0 cm^2^, LVEF > 50%, and transaortic mean gradient < 40 mmHg*. In these cases, we must evaluate the following parameters for defining severe AS: – Indexed AVA ≤ 0.6 cm^2^/m^2^ – High aortic valve calcium score – Systolic arterial pressure ≤ 140 mmHg – Indexed stroke volume < 35 mL/m^2^ Patients with all of the above parameters, but normal indexed stroke volume (> 35 ml/m²) are defined as having normal-flow, low-gradient AS. This entity has been recently described; evidence is scarce, and these patients appear to benefit from valve intervention when they are symptomatic^88^^,^^89^

* In cases of low-flow, low-gradient AS with preserved or low LVEF, we must pay attention to possible errors in echocardiographic measurement. AS: aortic stenosis; AVA: aortic valve area; LV: left ventricle; LVEF: left ventricular ejection fraction.

The second step is the evaluation of etiology (Table 23).^88^^,^^89^ In developed countries, there is greater prevalence of degenerative/calcification etiology in elderly patients, whereas, in developing countries, rheumatic and bicuspid etiologies are predominant in young patients. In Brazil, we may observe a bimodal peak in the prevalence AS. In other words, there are patients with all etiologies in different age ranges due to the transitional age pyramid typical of developing countries. The etiology of AS also reflects in the choice of treatment (Step 5). Patients with rheumatic AS are usually young, and have not been considered in transcatheter aortic valve implantation (TAVI) studies. The majority of patients studied had degenerative etiology. There is, however, already evidence regarding the procedure’s feasibility in patients with bicuspid aortic valve.^90^

**Table 23:** Step 2: Evaluation of severe aortic stenosis etiology^88^^,^^89^

	Etiological characteristics
Atherosclerotic/degenerative	Associated with old age Prevalence: 3% to 5% of the population over 75 years old Related to aortic valve calcification Presence of risk factors related to atherosclerosis
Rheumatic	Commissural fusion Mitral-aortic involvement Younger patients Associated with a range of aortic regurgitation degrees
Bicuspid	Prevalence: 2% of the population Associated with aortopathy (70% of cases) Latero-lateral orientation of the commissural cleft: predictor of the evolution of aortic stenosis

The third step is the evaluation of symptoms related to the VHD (Table 24). Intervention is unequivocally indicated for patients with severe AS and dyspnea, angina, or syncope.

**Table 24:** Step 3: Evaluation of severe aortic stenosis symptoms

	Symptoms
Dyspnea	Diastolic dysfunction: LV hypertrophy ➔ reduced compliance ➔ shifting of the ventricular pressure/volume curve up and to the left ➔ increased filling pressures ➔ pulmonary capillary hypertension Systolic dysfunction: related to afterload mismatch and low-flow/low-gradient states Patients with unclear symptomology (pseudo-asymptomatic) may undergo exercise test (ergometry or ergospirometry) for evaluation of dyspnea during exertion
Angina	Imbalance in oxygen supply/consumption in the hypertrophic myocardium Reduced myocardial perfusion gradient (elevated end diastolic pressure)
Syncope	Results from inability to increase cardiac output in situations of significant reduction in total peripheral resistance May result from use of vasodilators (common triggering agents) 50% of cases are associated with cardioinhibitory reflex

In cases where there are no symptoms, we must evaluate the presence of prognostic factors that justify indication of intervention (Table 25).^91^^–^^95^ The following prognostic factors are currently taken into consideration in the current guidelines:

**Table 25:** Step 4: Evaluation of severe aortic stenosis prognostic factors^91^^–^^98^

	Prognostic factors
Echocardiogram	LV dysfunction: LVEF < 50% Markers of very severe AS: AVA < 0.7 cm^2^, maximum aortic jet velocity > 5.0 m/s, mean transaortic gradient > 60 mmHg
Ergometry/ergospirometry test	Limited functional capacity Inadequate pressure response: increase in systolic arterial pressure less than 20 mmHg or systolic arterial pressure with a decrease greater than 10 mmHg Arrhythmias: ventricular tachycardia or more than 4 successive ventricular extrasystoles ST segment horizontal or descending depression ≥ 2 mm Contraindicated in symptomatic patients and/or patients with LV dysfunction

AVA: aortic valve area; LV: left ventricle; LVEF: left ventricular ejection fraction.

– Echocardiogram: LV dysfunction (LVEF < 50%) and/or markers of very severe AS (AVA < 0.7 cm^2^, maximum aortic jet velocity > 5.0 m/s, mean transaortic gradient > 60 mmHg).^96^
– Exercise test (ergometry): absence of inotropic reserve and/or low functional capacity, arterial hypotension during exertion (20 mmHg decrease in systolic arterial pressure) and/or presence of symptoms with low loads.^97^^,^^98^

The fifth and final step is choice of intervention (Tables 26 and 27 and Figures 5, 6, and 7). Transfemoral TAVI is preferable in relation to other thoracic access approaches (transaortic and transapical). Transfemoral approach is less invasive, and has a lower rate of complications. For this reason, other approachs are recommended only when there is a technical contraindication to femoral access.

**Table 26:** Step 5: Type of severe aortic stenosis intervention^90^^,^^99^^–^^132^

Type	Considerations
Aortic valve replacement surgery*	First choice for patients under 70 years without contraindication or high surgical risk* May be indicated for patients with intermediate risk or elderly patients with low risk, depending on the Heart Team’s decision and the availability of the transcatheter procedure
TAVI	Requires evaluation of the institutional Heart Team Transfemoral approach is preferred First choice in patients with prohibitive surgical risk, contraindications to surgery, frailty, or intermediate risk Expanded indication for patients with low surgical risk (STS < 4%, EuroSCORE II < 4%, logistic EuroSCORE < 10%) * Transfemoral access appears to be better than surgery in these patients There is a lack of data regarding TAVI in patients < 70 years and prosthesis durability Thus, in patients with low risk, age < 70 years, without other specific indications for TAVI, this procedure should be avoided Angiotomography of the aorta is the exam of choice for evaluating which access to use, valve size, type of valve, and feasibility of the procedure, as well as for predicting possible complications. Contraindicated in patients with estimated life expectancy of less than 12 months
Percutaneous balloon aortic valvuloplasty	“Bridge” for definitive procedures (surgery or TAVI) in patients with hemodynamic instability or advanced symptoms Palliative in cases with definitive contraindications to conventional surgery or TAVI.

* All current guidelines consider TAVI the preferred intervention, rather than surgery, for patients who are inoperable or frail and/or patients with high surgical risk (evaluated by the STS and EuroSCORE II scores). However, following the publication of American and European guidelines, 4 studies comparing TAVI and surgery in patients with low surgical risk were published. Meta-analysis of these studies demonstrated reduced 1-year mortality in transfemoral TAVI. These results suggest that transfemoral TAVI should be the preferred treatment in these patients. However, it is relevant to note that the mean age of the studied population was 75.4 years. Thus, in low-risk patients, extending to intermediate risk, we should avoid TAVI in patients under 70 years of age, until more robust data have been published regarding the durability of the prostheses. STS: Society of Thoracic Surgeons; TAVI: transcatheter aortic valve implantation.

**Table 27:** Aortic stenosis: Recommendations^1^^,^^2^^,^^90^^,^^99^^–^^132^

Intervention	Clinical condition	SBC	AHA	ESC
Surgical aortic valve replacement or TAVI*	Symptoms (NYHA FC ≥ 2, syncope and angina)	I A	I A	I B
	Asymptomatic, with prognostic factors: LVEF < 50%	I B	I B	I C
	Exercise test +	IIa B	IIa B	I C
	Asymptomatic, with very severe AS: AVA < 0.7 cm^2^ Maximum jet velocity > 5.0 m/s Mean transaortic gradient > 60 mmHg	IIa C	IIa B	IIa C (Elevated BNP; SPAP > 60 mmHg; maximum jet velocity > 5.5 m/s)
	**Special situations**			
	Severe low-flow, low-gradient AS with low LVEF: – With contractile reserve – Without contractile reserve + elevated aortic calcium score Severe symptomatic paradoxical AS	IIa B	IIa B	I C
		IIa C	–	IIa C
		IIa C	IIa C	IIa C
Choice of intervention, between surgery and TAVI**	Inoperable, prohibitive risk and/or frailty – TAVI – Surgery	I A	I A	I B
		IIb A	–	–
	High surgical risk – TAVI – Surgery	I A	I A	I B
		IIa A	I A	–
	Intermediate surgical risk – TAVI – Surgery	I A	IIa B	I B
		IIa A	I B	I B
	Low risk > 70 years – TAVI – Surgery	I A	–	–
		I A	I B	I B
	Low risk < 70 years – TAVI – Surgery	IIb C	–	–
		I A	I B	I B
Percutaneous balloon aortic valvuloplasty *	Symptomatic patients with important hemodynamic instability, temporarily impossible to perform definitive intervention (TAVI or conventional surgery) — “therapeutic bridge”	IIa C	IIb C	IIb C
	Palliative treatment in symptomatic patients, with contraindications to surgery and/or TAVI.	IIb C	–	–

* Mandatory prerequisite: evaluation by the institutional Heart Team, evaluating surgical risk, frailty, anatomical conditions, and comorbidities.

** Other aspects, such as the technical feasibility, risks and benefits of each procedure, patient choice, local experience, and availability of procedures, should also be taken into consideration when choosing the technique. The American and European guidelines were published before the studies on TAVI in low surgical risk patients. We should take these data into consideration when comparing the evidence of the 3 guidelines (SBC, AHA, and ESC). AHA: American Heart Association; AS: aortic stenosis; AVA: aortic valve área; ESC: European Society of Cardiology; FC: functional class; LVEF: left ventricular ejection fraction; SBC: Sociedade Brasileira de Cardiologia (Brazilian Society of Cardiology); TAVI: transcatheter aortic valve implantation.

**Figure 5:** Flowchart for decision making in aortic stenosis. BAV: balloon aortic valvuloplasty; ET: exercise test; LVEF: left ventricular ejection fraction; TAVI: transcatheter aortic valve implantation.

**Figure 6:** Flowchart for confirming anatomical severity of low-flow, low-gradient aortic stenosis with low ejection fraction. CT: computerized tomography; LVEF: left ventricular ejection fraction.

**Figura 7:** Flowchart for decision making in paradoxical aortic stenosis. BAV: balloon aortic valvuloplasty; BP: blood pressure; ET: exercise test; iAVA: indexed aortic valve area; LVEF: left ventricular ejection fraction; TAVI: transcatheter aortic valve implantation.

All current guidelines consider TAVI the preferred intervention, rather than surgery, for patients who are inoperable or frail and/or patients with high surgical risk (evaluated by the STS and EuroSCORE II scores).^99^^–^^113^ However, following the publication of American and European guidelines, 4 studies comparing TAVI and surgery in patients with low surgical risk were published. Meta-analysis of these studies demonstrated reduced 1-year mortality in transfemoral TAVI. These results suggest that transfemoral TAVI should be the preferred treatment in these patients. However, it is relevant to note that the mean age of the studied population was 75.4 years. Thus, in low-risk patients, extending to intermediate risk, we should avoid TAVI in patients under 70 years of age, until more robust data have been published regarding the durability of the prostheses. ^100^^,^^114^^–^^120^

Another relevant aspect which is unanimous in Brazilian and international guidelines is the need for a Heart Team to evaluate each case. Other aspects, such as technical feasibility, risks and benefits of each procedure, patient choice, local experience, and availability of procedures, should also be taken into consideration when choosing the type of intervention.

The following groups of patients should be monitored frequently, due to the risk of progression of the VHD (Table 28):

**Table 28:** Aortic stenosis: Individualized follow-up^1^^,^^2^

Aortic stenosis	Follow-up	SBC	AHA	ESC
Severe and asymptomatic, without prognostic factors	Clinical and echocardiographic evaluation	Every 6 months	Every 0.5 to 1 year	Every 6 months
	Concomitant intervention in patients who will undergo another cardiac surgical procedure (coronary revascularization, ascending aorta, or other valve procedures)	I C	I B	I C
Moderate (AVA between 1.0 and 1.5 cm² and mean transaortic gradient 25 – 39 mmHg)	Clinical and echocardiographic evaluation	Every year	Every 1 to 2 years	Every year
	Concomitant intervention in patients who will undergo another cardiac surgical procedure (coronary revascularization, ascending aorta, or other valve procedures)	IIa C	IIa C	IIa C
Mild (AVA > 1.5 cm² and mean transaortic gradient < 25 mmHg)	Clinical and echocardiographic evaluation	Every 2 to 3 years	Every 3 to 5 years	Every 2 to 3 years

AHA: American Heart Association; AVA: aortic valve area; LV: left ventricle; SC: European Society of Cardiology; SBC: Sociedade Brasileira de Cardiologia (Brazilian Society of Cardiology).

– Severe asymptomatic AS, without prognostic factors: To date, these patients are indicated for valve surgery only if other invasive cardiovascular procedures are indicated (coronary revascularization, ascending aorta, or other valve procedures). Studies are underway to evaluate the benefit of early intervention in this group of patients.
– Moderate AS, defined as AVA between 1.0 and 1.5 cm² and mean transaortic gradient 25 to 39 mmHg: These patients are indicated for valve surgery only if other invasive cardiovascular procedures are indicated (coronary revascularization, ascending aorta, or other valve procedures).
– Mild AS, defined as AVA > 1.5 cm² and mean transaortic gradient < 25 mmHg: Clinical and echocardiographic follow-up.

## 9. Chronic Aortic Regurgitation

The five-step clinical approach (Figure 8) is also recommended for management of chronic aortic regurgitation (AR). The first step consists of charactering AR anatomical severity, especially identifying patients with anatomically severe AR. Table 29 shows the main findings of clinical examination and complementary methods for defining severe AR.^133^^,^^134^ In general, transthoracic echocardiogram continues to be the main tool for diagnosing and scoring the severity of AR. Three-dimensional echocardiography has been increasingly incorporated into complementary evaluation, especially in cases where two-dimensional analysis is limited (eccentric jets or anatomical determination, for example, in bicuspid valve disease). Furthermore, there has recently been an increase of studies on cardiac magnetic resonance for evaluation of AR, making it possible to acquire new diagnostic and prognostic parameters, such as regurgitant fraction and estimated LV end diastolic volume.^134^

**Table 29:** Step 1: Diagnosis of severe aortic regurgitation ^133^^,^^134^

	Characteristics of severe aortic regurgitation
Physical examination	Decrescendo blowing diastolic murmur with hypophonetic second heart sound Hyperflow midsystolic murmur Austin-Flint murmur (AR jet does not allow mitral valve opening, generating a rumbling diastolic murmur) Water hammer pulse or Corrigan’s pulse: rapid increase and high amplitude Divergence between systolic and diastolic pressures Clinical signs of increased pulse pressure: Musset’s sign, Becker’s sign, carotid dance, Muller’s sign, Quincke’s sign, Rosenbach’s sign, Gerhard’s sign, Traube’s sign, Duroziez’s sign, Mayne’s sign, and Hill’s sign
Electrocardiogram	Signs of left chamber overload
Chest radiography	Enlarged cardiac silhouette due to LV dilation Signs of aortic dilation or ectasia
Echocardiogram	Evaluation of the valve disease etiology, ascending aorta diameter, ventricular diameters, and ventricular function. Quantification of regurgitation: – Vena contracta > 0.6 cm – Jet width > 0.65 cm – Jet area ≥ 60% – Regurgitant fraction ≥ 50% – Regurgitant volume ≥ 60 mL/beat – EROA ≥ 0.30 cm²
Hemodynamic study	Necessary in cases of discordance between clinical and echocardiographic findings (elevated left ventricular end diastolic pressure, aortic regurgitation during aortography)
Magnetic resonance	Evaluation of the aorta Evaluation of ventricular function in borderline cases Evaluation of valve function in cases of disagreement between clinical and echocardiographic findings New prognostic factors: regurgitant fraction and LV end diastolic volume
Angiotomography of the aorta	Evaluation of the aorta

AR: aortic regurgitation; EROA: effective regurgitant orifice area; LV: left ventricle.

**Figure 8:** Flowchart for decision making in chronic aortic regurgitation. LVDD: left ventricular diastolic diameter; LVEF: left ventricular ejection fraction; LVSD: left ventricular systolic diameter.

For the second step (Table 30), it is necessary to verify the etiology of AR. From the etiopathogenic point of view, chronic AR is related to anatomical abnormalities related to the valve leaflets and/or pathologies of the aortic valve annulus. The following causes are related to dysfunction of the valve leaflets: rheumatic fever (still one of the main etiologies in Brazil), infective endocarditis (IE), degenerative causes, congenital malformations such as bicuspid valve disease, and myxomatous degeneration. With respect to abnormalities related to the aortic valve annulus, it is worth underscoring dissection of the ascending aorta, aneurysmatic dilatation (mainly provoked by systemic arterial hypertension and collagen diseases such as Marfan and Ehlers-Danlos syndromes), seronegative spondyloarthropathies (ankylosing spondylitis and Reiter’s disease), syphilitic aortitis, and Takayasu arteritis.^135^^,^^136^

**Table 30:** Step 2: Evaluation of severe aortic regurgitation etiology^135^^,^^136^

	Etiological characteristics
Rheumatic	High prevalence in Brazil Generally associated with mitral lesion Frequent in young adults
Atherosclerotic	Generally associated with AS Frequent in the elderly population
Bicuspid	Associated with abnormalities in the aorta (40% of cases – aneurysm, dissection, coarctation) Frequent in young adults
Diseases related to altered geometry of the aortic root	Systemic arterial hypertension, dissection of the ascending aorta, Marfan syndrome, ankylosing spondylitis, syphilitic aortitis, osteogenesis imperfecta, Ehlers-Danlos syndrome, Reiter’s syndrome, subaortic stenosis, and interventricular septal defect with prolapse of the aortic cusp
Others	Infective endocarditis, myxomatous degeneration, traumatic lesions, rheumatoid arthritis

AS: aortic stenosis.

The third step (Table 31) is characterized by evaluation of symptoms related to AR. Identification of symptoms may be a difficult task during routine healthcare, especially in elderly patients who frequently have physical self-limitation. In these cases, provocative functional tests, such as ergospirometry, can assist in identification of these “pseudo-asymptomatic” patients. Given the high morbidity and mortality related to symptoms, once they are identified, patients should be referred for surgical intervention.

**Table 31:** Step 3: Evaluation of severe aortic regurgitation symptoms

Symptoms	
Dyspnea	Occurs due to increased end diastolic pressure secondary to blood volume overload in the LV and consequent pulmonary capillary congestion.
Angina	Occurs due to reduced myocardial reserve. Nocturnal angina may occur due to increased valve regurgitation resulting from bradycardia during sleep.
Syncope	Low effective cardiac output

LV: left ventricle.

The fourth step (Table 32) focuses on evaluation of prognostic factors. This step is especially relevant for asymptomatic patients. The main prognostic factor of AR is low LV systolic function, related to systolic stress and ventricular dilation. In a retrospective study, Chaliki et al. found reduced survival in patients who had AR with LVEF below 50%. Postoperative mortality rates were also influenced by ventricular function (14% for patients with LVEF below 35%, 6.7% for LVEF between 35% and 50%, and 3.7% for patients with LVEF above 50%, p = 0.02). ^137^

**Table 32:** Step 4: Evaluation of severe aortic regurgitation prognostic factors^134^^,^^137^^,^ ^137^^–^^139^^,^^141^

	Prognostic factors
Echocardiogram	LVEF < 50% LVDD > 70 mm (non-rheumatic) and > 75 mm (rheumatic) LVSD > 50 mm (non-rheumatic) and > 55 mm (rheumatic) Indexed LVSD > 25 mm/m²
Magnetic resonance	Presence of late Gadolinium enhancement images Regurgitant fraction > 33% LV end diastolic volume > 246 mL
Angiotomography	Bicuspid valve with indication of intervention + aortic root > 45 mm

LV: left ventricle; LVDD: left ventricular diastolic diameter; LVEF: left ventricular ejection fraction; LVSD: left ventricular systolic diameter.

Ventricular remodeling continues to show clinical ambivalence: on one hand, increased ventricular diameters is an adaptive mechanism to volume overload; on the other hand, ventricular remodeling may determine worse prognosis, especially in non-rheumatic populations. In a Brazilian study carried out with 75 asymptomatic patients with rheumatic severe AR, the strategy of indicating surgical treatment based on the appearance of symptoms, even in patients with LV diastolic diameter (LVDD) greater than 75 mm and LVSD greater than 55 mm, with normal LVEF, was capable of promoting improvement in quality of life and reversal of dilation, with a 10-year survival rate of 90.6%.^138^ On the other hand, prospective studies with predominant non-rheumatic AR patients found that LVSD above 50 mm was associated with composite clinical outcomes (death, symptoms, and/or ventricular dysfunction) of up to 19% yearly. There is also evidence that it would be more appropriate to used diameters indexed by body surface area, especially in women. A study of 246 patients with asymptomatic AR found that indexed LVSD values equal to or greater than 25 mm/m^2^ were associated with outcomes (mortality, symptoms, and ventricular dysfunction).^139^ More recently, studies have evaluated the role of brain natriuretic peptide (BNP) in AR. Cutoff values of 130 pg/mL for BNP and 602 pg/mL for NT-pro-BNP have been associated with adverse clinical outcomes. The combination of these values of BNP with echocardiographic parameters may improve the ability to stratify asymptomatic patients. Persistent elevations in BNP during clinical follow-up have also been related to adverse clinical events.^140^

Echocardiographic parameters such as longitudinal stress are also predictors of evolution in asymptomatic AR, and they also influence postoperative results. The limitation to the clinical use of longitudinal stress in AR lies in the divergence regarding cutoff points.

Another prognostic factor related to AR is late enhancement myocardial fibrosis. Cardiac magnetic resonance with late enhancement is the main imaging method capable of quantification. Studies have demonstrated that the presence of myocardial fibrosis influences the postoperative period, and it is associated with persistence of symptoms, failure to recover ventricular function, and higher mortality.^141^ Also with respect to magnetic resonance, new studies have demonstrated that regurgitant fraction above 33% and LV end diastolic volume above 246 ml were associated with lower surgery-free survival. These new parameters may improve stratification of asymptomatic patients, thus ensuring more precise surgical indications.^134^

Finally, the fifth step is to define the intervention in AR (Tables 33 and 34). Surgical aortic valve replacement remains the first choice.^142^^,^^143^ Surgical mortality rates range from 1% (valve replacement procedure alone) to 7% (combined procedures). The presence of symptoms, reduced LVEF, and excessive LV remodeling entail worse prognosis, and they are, therefore, the main indications for surgical treatment. As previously stated, new prognostic factors related to myocardial fibrosis, left ventricular remodeling, and biomarkers may represent potential future parameters for intervention. The clinical follow-up of patients without indication of intervention is described in Table 35.

**Table 33:** Step 5: Type of severe aortic regurgitation intervention^142^^,^^143^

Type of intervention	Considerations
Surgery (aortic valve replacement)	Treatment of choice Valve replacement combined with correction of the ascending aorta, when indicated
TAVI	Requires further studies

TAVI: transcatheter aortic valve implantation.

**Table 34:** Aortic regurgitation: Recommendations^1^^,^^2^^,^^142^^,^^143^

Intervention	Clinical condition	SBC	AHA	ESC
Aortic valve replacement surgery	Symptoms	I B	I B	I B
	LVEF < 50%	I B	I B	I B
	Ventricular diameters	IIa B Rheumatic LVDD > 75 mm or LVSD > 55 mm IIa B Non-rheumatic LVDD > 70 mm or LVSD > 50 mm or indexed LVSD >25 mm/m²	IIa C LVDD > 70 mm or LVSD > 50 mm or indexed LVSD > 25 mm/m²	IIa B LVDD > 70 mm or LVSD > 50 mm or indexed LVSD > 25 mm/m²
Transcatheter aortic valve implantation – TAVI *	Symptomatic, with life expectancy > 1 year and contraindications/prohibitive risk of conventional surgery	IIb C*	–	–

* Consider discussion in the Heart Team. AHA: American Heart Association; ESC: European Society of Cardiology; LVDD: left ventricular diastolic diameter; LVEF: left ventricular ejection fraction; LVSD: left ventricular systolic diameter; SBC: Sociedade Brasileira de Cardiologia (Brazilian Society of Cardiology).

**Table 35:** Aortic regurgitation: Individualized follow-up^1^^,^^2^

Aortic regurgitation	Follow-up	SBC	AHA	ESC
Severe and asymptomatic, without prognostic factors	Clinical and echocardiographic evaluation	Every 0.5 to 1 year	Every 0.5 to 1 year	Every 3 to 6 months
	Concomitant intervention in patients who will undergo another cardiac surgical procedure (coronary revascularization, ascending aorta, or other valve procedures)	I C	I C	I C
Moderate (Vena contracta 0.3 – 0.6 cm, jet width 0.25 – 0.64 cm, regurgitant fraction 30% – 49%, regurgitant volume 30 – 59 mL/beat, EROA 0.10 – 0.29 cm²)	Clinical and echocardiographic evaluation	Every 1 to 2 years	Every 1 to 2 years	Every 1 to 2 years
	Concomitant intervention in patients who will undergo another cardiac surgical procedure (coronary revascularization, ascending aorta, or other valve procedures)	IIa C	IIa C	–
Mild (Vena contracta < 0.3 cm, jet width < 0.25 cm, regurgitant fraction < 30%, regurgitant volume < 30 ml/beat, EROA < 0.10 cm²)	Clinical and echocardiographic evaluation	Every 3 to 5 years	Every 3 to 5 years	Every 1 to 2 years

AHA: American Heart Association; EROA: effective regurgitant orifice area; ESC: European Society of Cardiology; SBC: Sociedade Brasileira de Cardiologia (Brazilian Society of Cardiology).

## 10. Tricuspid Stenosis

Tricuspid stenosis (TS) is a rare VHD, usually associated with tricuspid regurgitation (TR). Echocardiography remains the main tool to define anatomical severity (Table 36). ^144^

**Table 36:** Step 1: Diagnosis of severe tricuspid stenosis^144^

	Characteristics of severe tricuspid stenosis
Physical examination	Early opening snap Hyperphonetic first heart sound Rumbling diastolic murmur, with presystolic reinforcement in patients in sinus rhythm in the left sternal border, increasing with inspiration Systemic congestion: hepatomegaly, ascites, lower limbs edema, jugular venous stasis, Kussmaul’s sign
Electrocardiogram	Overload of the RA AF
Chest radiography	RA enlargement
Echocardiogram	Tricuspid valve area ≤ 1.0 cm^2^ Mean diastolic RA/right ventricle gradient ≥ 5mmHg Isolated RA enlargement Tricuspid PHT ≥ 190 ms
Hemodynamic study	Cases of clinical and echocardiographic discordance Diastolic RA/right ventricle gradient ≥ 5 mmHg
Magnetic resonance	Cases of clinical and echocardiographic discordance or limited quality of echocardiographic image

AF: atrial fibrillation; PHT: pressure half time; RA: right atrium.

The most common etiology of TS is rheumatic disease. It generally occurs concomitantly to mitral valve and/or aortic valve disease. Thickening and cusp retraction occur with commissural involvement. Other possible causes of TS, which are even rare, are described in Table 37.^145^^–^^147^

**Table 37:** Step 2: Evaluation of severe tricuspid stenosis etiology^145^^–^^147^

	Etiological characteristics
Rheumatic	Most prevalent cause Associated with other valvular heart diseases Thickening with cusp retraction Commissural involvement Frequent in young adults
Other	Infective endocarditis Systemic lupus erythematosus Carcinoid syndrome Congenital deformities Atrial myxoma Actinic injury (post-radiotherapy) Deposit disease: amyloidosis, Fabry disease Whipple disease

Both symptoms and physical examination are usually limited to patients with anatomically severe TS. The most commonly found symptom is fatigue, which may be associated with symptoms of right-sided heart failure (Table 38).

**Table 38:** Step 3: Evaluation of severe tricuspid stenosis symptoms

	Symptoms
Fatigue	Main symptom Associated with lower limbs pain and edema Absence of dyspnea May be associated with palpitations, ascites, or signs of hepatic dysfunction

When asymptomatic patients have severe TS, it is necessary to evaluate whether or not there present prognostic factors (Table 39).

**Table 39:** Step 4: Evaluation of severe tricuspid stenosis prognostic factors

	Prognostic factors
Electrocardiogram	AF
Systemic congestion	Evaluation of hepatic impairment (altered enzymes or coagulogram)

AF: atrial fibrillation.

In the presence of these symptoms or prognostic factors, intervention is indicated. In spite of the rarity of cases and the scarcity of data in the literature, percutaneous balloon tricuspid valvuloplasty (PBTV) remains the treatment of choice (Tables 40 and 41 and Figure 9).^148^

**Table 40:** Step 5: Type of severe tricuspid stenosis intervention^148^

Type	Considerations
Percutaneous balloon tricuspid valvuloplasty	Treatment of choice Moderate TR is not a contraindication Contraindicated in the presence of atrial thrombus despite anticoagulation and/or vegetation
Tricuspid valve replacement	Option when balloon valvuloplasty is contraindicated Bioprosthesis is preferable Preferable if associated with surgery for treatment of mitral valve disease

**Table 41:** Tricuspid stenosis: Recommendations^1^^,^^2^^,^^148^

Intervention	Clinical condition	SBC	AHA	ESC
Percutaneous balloon tricuspid valvuloplasty	Severe symptomatic TS alone, without contraindications	IIa C	IIb C	–
	Concomitant PBMV	I C	I C	–
	PBTV with severe TR	III	–	–
Tricuspid valve replacement or repair (commissurotomy)	Severe, symptomatic TS with contraindication to PBTV	I C	I C	I C
	Severe, symptomatic TS alone	IIa C	I C	I C
	Bioprosthesis is preferable, when valve replacement is indicated	I C	–	–

AHA: American Heart Association; PBMV: percutaneous balloon mitral valvuloplasty; PBTV: percutaneous balloon tricuspid valvuloplasty; ESC: European Society of Cardiology; SBC: Sociedade Brasileira de Cardiologia (Brazilian Society of Cardiology); TR: tricuspid regurgitation; TS: tricuspid stenosis.

**Figure 9:** Flowchart for decision making in tricuspid stenosis. AF: atrial fibrillation; PBTV: percutaneous balloon tricuspid valvuloplasty.

## 11. Tricuspid Regurgitation

Patients with mild TR usually do not require any type of treatment. Patients with moderate to severe TR will need specific follow-up, especially in order to identify the etiology of the VHD and the repercussions associated (Table 42).^149^

**Table 42:** Step 1: Diagnosis of severe tricuspid regurgitation^149^

	Characteristics of severe tricuspid regurgitation
Physical examination	Pathological jugular venous stasis Hyperphonetic second heart sound (pulmonary arterial hypertension) Regurgitative systolic murmur in the left sternal border associated with Rivero-Carvallo sign Hepatomegaly
Electrocardiogram	Right chamber overload AF
Chest radiography	Signs of enlarged right chambers Pulmonary congestion, only when associated with left side valvular disease Enlargement pulmonary trunk
Echocardiogram	EROA ≥ 0.40 cm² Reverse flow in hepatic veins Regurgitant volume > 45 ml/beat Dense, triangular regurgitant volume, with early peak on continuous Doppler. Vena contracta ≥ 0.7 cm Annulus diameter ≥ 40 mm Failed cusp coaptation
Hemodynamic study	Case of clinical and echocardiographic discordance Measures SPAP in cases with failed cusp coaptation
Magnetic resonance	Case of clinical and echocardiographic discordance or limited quality of echocardiographic image

AF: atrial fibrillation; EROA: effective regurgitant orifice area; SPAP: systolic pulmonary artery pressure.

TR is usually functional, secondary to dilation of the tricuspid valve annulus, mainly secondary the left heart chambers valve diseases or cardiomyopathies and/or PH. Cases of primary TR are generally related to rheumatic disease, interventions (repeated endomyocardial biopsies, presence of pacemaker electrodes, or implanTable cardioverter defibrillator), consequence of IE or other rare causes (Table 43).^150^

**Table 43:** Step 2: Evaluation of severe tricuspid regurgitation etiology^150^

	Etiological characteristics
Primary	Rheumatic fever Prolapse and myxomatous degeneration Actinic injury due to radiation (post-radiotherapy) Closed chest trauma Infective endocarditis Repeated endomyocardial biopsies Carcinoid syndrome Congenital (Ebstein) Pacemaker electrodes or defibrillator
Secondary	Dilation of the tricuspid annulus (> 40 mm or > 21 mm/m²) Left heart valve disease Long-duration AF Primary PH Right ventricular cardiomyopathy (ischemic, arrhythmogenic dysplasia, non-compacted myocardium, hypertrophic cardiomyopathy) Constrictive pericarditis
Rare causes	Rheumatologic diseases Medication (methysergide or anorexigenic drugs) Fabry disease

AF: atrial fibrillation; PH: pulmonary hypertension.

During severe TR natural history, symptoms may arise which will have a significant impact on decision making (Table 44).

**Table 44:** Step 3: Evaluation of severe tricuspid regurgitation symptoms

Symptoms	
Dyspnea (NYHA FC II to IV)	Secondary to left heart disease (pulmonary capillary congestion, pulmonary arterial hypertension). Dyspnea during exertion and nocturnal paroxysmal
Fatigue	Main symptom Associated with lower limbs pain and edema More common in right heart failure

FC: functional class; NYHA: New York Heart Association.

On the other hand, even in asymptomatic patients, right ventricular remodeling can develop, which may justify valve intervention. Thus, right ventricular dilation or dysfunction (except for severe right ventricular dysfunction) should be considered as a prognostic factor (Table 45).

**Table 45:** Step 4: Evaluation of severe tricuspid regurgitation prognostic factors

Prognostic factors	
Echocardiogram	Primary TR: progressive right ventricular dilation or dysfunction

TR: tricuspid regurgitation.

New data have shown the prognostic importance of TR. A recently published study found a prevalence of moderate to severe TR of 0.55%, where 72% of cases were secondary to left VHD (49.5%) or PH (23%). Only 8% of cases were isolated TR. Patients with moderate to severe TR alone show a higher mortality rate (relative risk 1.68, with 95% CI 1.04 to 2.6, p = 0.03), confirming data from the same group published in 2014.^151^ The increase in mortality has also been shown in a recent meta-analysis, including 70 studies, which found almost two-fold mortality in patients with moderate to severe TR (relative risk 1.95, 95% CI 1.75 to 2.17). TR was an independent mortality predictor even after adjusting for the presence of right ventricular dysfunction, PH, AF, MR, or LV dysfunction.^152^

The interventional treatment of choice, when indicated, is tricuspid valve repair, with a prosthetic ring capable of reducing the tricuspid annulus diameter, improving valve leaflet coaptation, and correcting regurgitation. Valve replacement is reserved for patients who do not have anatomical conditions for repair. It should be noted that the isolated surgical approach to the tricuspid valve currently continues to be rarely indicated, and has surgical mortality rates varying from 8.8% to 9.7%. However, there are still no data showing improved survival with TR surgical treatment alone, despite the increased mortality rate in patients with moderate to severe TR in clinical treatment. For this reason, the main reason for surgical indication, in this population, is still to improve symptoms and prevent severe right ventricular dysfunction.^153^^–^^155^

The number of studies on percutaneous interventional treatment of TR has increased. Several devices have been developed, with strategies based on reducing the tricuspid valve annulus, improving coaptation between the leaflets, or even on transcatheter valve implantation. Further data will be available soon (Tables 46 and 47 and Figure 10) ^149^^,^^156^^–^^158^

**Table 46:** Step 5: Type of severe tricuspid regurgitation intervention^149^^,^^151^^–^^158^

Type	Considerations
Tricuspid repair with a prosthetic ring	Treatment of choice
	Indications: – Left valvular heart disease intervention in the presence of tricuspid annulus ≥ 40 mm and/or moderate to severe tricuspid regurgitation – tricuspid regurgitation alone, refractory to clinical treatment, low surgical risk, without contraindications.
	Contraindications: severe right ventricular systolic dysfunction
Surgical valve replacement	If repair is possible Bioprosthesis is preferable
Transcatheter tricuspid valve implantation	Refractory symptoms, with contraindication or high surgical risk (currently under study)

**Table 47:** Tricuspid regurgitation: Recommendations^1^^,^^2^^,^^149^^,^^151^^–^^158^

Intervention	Clinical condition	SBC	AHA	ESC
Tricuspid repair with a prosthetic ring	Left valvular heart disease intervention and severe TR	I C	I C	I C
	Left valvular heart disease intervention and tricuspid annulus ≥ 40 mm	IIa C	IIa B	IIa C
	Left valvular heart disease intervention, severe TR, and signs of right ventricular dysfunction	IIa C	IIa B	IIa C
	Left valvular heart disease intervention, moderate to severe TR, and/or annulus ≥ 40 mm and SPAP ≥ 70 mmHg	IIa C	IIb C	IIa C
	Severe TR alone, refractory to clinical treatment	IIa C	IIa C	IIa C
	Severe primary asymptomatic TR alone, with right ventricular dilation or progressive dysfunction	IIb C	IIb C	IIa C
Surgical valve replacement	Repair not possible	I C	I C	I C
	Bioprosthesis preferable	I B	–	–
Transcatheter tricuspid valve implantation	Refractory to clinical treatment, with contraindication or high surgical risk (currently under study)	IIb C*	–	–

* Consider discussion in the Heart Team. AHA: American Heart Association; ESC: European Society of Cardiology; SBC: Sociedade Brasileira de Cardiologia (Brazilian Society of Cardiology); TR: tricuspid regurgitation.

**Figure 10:** Flowchart for decision making in tricuspid regurgitation. RV: right ventricular.

## 12. Prosthetic Valve Dysfunction

After valve replacement surgery, periodical clinical and echocardiographic follow-up is needed for early detection of prosthesis dysfunction, as well as identification of anatomical and functional symptoms or prognostic factors.

The main test for diagnosis of prosthesis dysfunction is transthoracic echocardiogram. Nonetheless, transesophageal echocardiogram and aorta angiotomography triggered with electrocardiogram (EKG) are useful especially in the evaluation of bioprosthesis thrombosis (Tables 48 and 49).^159^

**Table 48:** Step 1: Diagnosis of severe prosthetic valve dysfunction

	Characteristics of severe prosthetic valve dysfunction
Physical examination	Clinical signs according to the predominant type of prosthesis dysfunction
Electrocardiogram	Abnormalities according to the predominant type of prosthesis dysfunction
Chest radiography	Abnormalities according to with the predominant prosthesis dysfunction
Transthoracic echocardiogram	Evaluation the type of valve dysfunction and confirmation severity of the dysfunction – thickening of leaflets – calcification and mobility of leaflets – reduced EOA – transvalvular gradient – valve regurgitation Evaluation of ventricular systolic dysfunction Progressive evaluation of the cardiac chambers diameters
Transesophageal echocardiogram	Inadequate window for transthoracic echocardiogram Severe paravalvular regurgitation with favorable anatomy for percutaneous intervention Indicated to improve anatomical evaluation
Hemodynamic study with manometry	Cases of clinical and echocardiographic discordance
Angiotomography of the aorta triggered with EKG	Evaluation of the aorta Evaluation of bioprosthesis thrombosis and TAVI

EOA: effective orifice area; EKG: electrocardiogram; TAVI: transcatheter aortic valve implantation.

**Table 49:** Step 2: Evaluation of severe prosthetic valve dysfunction etiology^159^

	Etiological characteristics
Prosthesis stenosis	**Mechanical prosthesis:** thrombosis pannus **Biological prosthesis:** leaflets degeneration leaflets calcification prosthesis-patient mismatch (indexed EOA ≤ 0.85 cm^2^/m^2^)
Prosthesis regurgitation	**Central:** leaflets degeneration (rupture, perforation) leaflets calcification **Paravalvular:** infective endocarditis annulus degeneration

EOA: effective orifice area.

The most frequently symptom is dyspnea, resulting from pulmonary capillary congestion (Table 50).

**Table 50:** Step 3: Evaluation of severe prosthetic valve dysfunction symptoms

Symptoms	
Dyspnea (NYHA FC II to IV)	Pulmonary capillary congestion according to predominant dysfunction

FC: functional class; NYHA: New York Heart Association. FC: functional class; NYHA: New York Heart Association.

The definition of prognostic factors in prosthesis dysfunction is complex. Patient usually already has PH, ventricular dilation, or ventricular dysfunction as a result of prior VHD. Accordingly, the progression of these abnormalities should be taken into consideration for indication of intervention (Table 51).

**Table 51:** Step 4: Evaluation of severe prosthetic valve dysfunction prognostic factors

	Prognostic factors
Echocardiogram	Progression of ventricular systolic dysfunction Progression of LV remodeling (in the event that initial diameters are elevated) PH Severe bioprosthesis calcification
Hemolytic anemia	Occurs in cases of severe prosthetic valve regurgitation, especially if it is paravalvular

LV: left ventricle; PH: pulmonary hypertension.

New procedures, such as percutaneous treatment of paravalvular regurgitation and valve-in-valve, are already included in recent guidelines (Tables 52 and 53).^159^^–^^162^

**Table 52:** Step 5: Type of severe prosthetic valve dysfunction intervention^159^^–^^162^

Type	Considerations
Surgery (valve re-replacement)	Treatment of choice Indications: severe prosthetic valve dysfunction, with symptoms and/or severe hemolytic anemia
Transcatheter intervention – valve-in-valve	Mitral or aortic bioprosthesis dysfunction in symptomatic high surgical risk or inoperable patients (before Heart Team evaluation)
Percutaneous occlusion of paravalvular regurgitation	Severe paravalvular regurgitation associated with hemolytic anemia or heart failure symptoms (NYHA FC III/IV), in patients with high surgical risk and favorable anatomy for the procedure

FC: functional class.

**Table 53:** Prosthetic valve dysfunction: Recommendations^1^^,^^2^^,^^159^^–^^162^

Intervention	Clinical condition	SBC	AHA	ESC
Prosthesis replacement surgery	Symptomatic severe prosthetic valve dysfunction	I B	I B	I C
	Hemolytic anemia	IB	I B	I C
	Severe asymptomatic prosthetic valve dysfunction, with low surgical risk	IIa C	IIa C*	IIa C
Percutaneous occlusion of paravalvular regurgitation	Hemolysis or symptoms, with favorable anatomy and high surgical risk, before Heart Team evaluation.	IIa B	IIa B	–
Valve-in-valve	Severe bioprosthesis dysfunction, in high surgical risk or inoperable symptomatic patients, before Heart Team evaluation.	IIa B	IIa B	IIa C

* Aortic bioprosthesis with regurgitation. AHA: American Heart Association; ESC: European Society of Cardiology; SBC: Sociedade Brasileira de Cardiologia (Brazilian Society of Cardiology).

## 13. Multivalvular Disease

Multivalvular disease is a primary involvement of two or more valves. This classification excludes valve involvement secondary to a primary VHD, which is the case with functional TR as a consequence of mitral valve disease and MR secondary to LV remodeling as a consequence of aortic VHD (Table 54).^163^^–^^165^

**Table 54:** Step 1: Diagnosis of severe multivalvular disease^163^^–^^165^

	Characteristics of severe multivalvular disease
Physical examination	Presence of murmurs distinctly characterized as mitral and aortic – regurgitation, stenosis, or double lesion. Rule out the possibility of murmur caused by hemodynamic interference (for example, Austin-Flint murmur) Rule out the possibility of valve involvement secondary to a primary valvular heart disease (for example, TR secondary to mitral disease) Physical examination is especially important for defining predominance of one of the valvular heart diseases
Electrocardiogram	Left ventricular hypertrophy and/or left atrial enlargement, depending on the predominant valvular heart disease AF in severe mitral valvular heart diseases
Chest radiography	Increased cardiothoracic index, especially in association with regurgitant valvular diseases Signs of pulmonary congestion Signs of right ventricular overload in associated mitral stenotic lesion
Echocardiogram	Echocardiographic findings vary by valvular heart disease
Hemodynamic study	Indicated when there is disagreement between clinical and echocardiographic findings

AF: atrial fibrillation; LV: left ventricle; TR: tricuspid regurgitation.

In Brazil, multivalvular disease is the result of rheumatic involvement in most cases; there has been, however, a progressive increase in degenerative calcific mitral-aortic disease (Table 55).^159^

**Table 55:** Step 2: Evaluation of severe multivalvular disease etiology^159^^,^^163^^–^^165^

	Etiological characteristics
Rheumatic fever	> 95% of cases Typical in young patients Frequent extemporaneous evolution Symptoms between 20 and 40 years Commissural fusion, thickening of leaflets, frequent double dysfunction – complex pathophysiology Impaired subvalvular apparatus
Infective endocarditis	Valve regurgitation due to destruction of the mitral and/or aortic apparatus Aortic-mitral metastatic infection
Valvular apparatus calcification	Elderly patients Associated with degenerative aortic valvular disease Calcification of the mitral valve annulus with caseous calcification Absence of commissural fusion Related to aortic and coronary calcification
Marfan or Ehlers-Danlos syndrome	Mitral and aortic valve regurgitation Investigate involvement of the ascending aorta

Symptoms are generally associated with the most severe valvular disease, and, in cases where both are equally severe, the most proximal valvular disease tends to prevail (Table 56).

**Table 56:** Step 3: Evaluation of symptoms

	Symptoms
Dyspnea (NYHA FC II to IV)	Main symptom Initially with events that increase pulmonary capillary pressure May be accompanied by palpitations, hemoptysis, dysphonia, dysphagia, or cough Associated right heart failure in patients with pulmonary hypertension
Precordial pain	Especially when associated with regurgitant or stenotic aortic valvular heart disease May be caused by PH
Low output or syncope	Especially present with associated AS and MR

AS: aortic stenosis; MR: mitral regurgitation; PH: pulmonary hypertension.

Prognostic factors, when present, result from the most severe valvular disease (Table 57).

**Table 57:** Step 4: Evaluation of severe multivalvular disease prognostic factors

	Prognostic factors
Pulmonary hypertension	Resting SPAP ≥ 50 mmHg Most often present with associated MS Symptoms of right heart failure Related to increased surgical risk
Recent onset AF	Related to LA remodeling
Increased ventricular diameters	Consider diameters depending on the type of valve lesion

LA: left atrium; MS: mitral stenosis; SPAP: systolic pulmonary artery pressure.

The standard treatment of mitral-aortic diseases with symptoms and/or prognostic factors is surgical; nonetheless, transcatheter strategies may be indicated in select cases, especially in patients assumed to be at high risk for conventional surgery (Tables 58 and 59).^163^^–^^165^

**Table 58:** Step 5: Type of severe multivalvular disease intervention^163^^–^^165^

Type	Considerations
Percutaneous balloon mitral valvuloplasty	Cases of severe MS with favorable anatomy and moderate aortic valvular heart disease
Surgical treatment (commissurotomy or valve replacement)	Conservative mitral valve surgery when stenosis is predominant Avoid aortic valve repair – frequent recurrence of valvular heart disease and symptoms, even with good immediate results Treatment of anatomically moderate valvular heart disease concomitant to intervention for severe valvular disease
Transcatheter treatment – valve-in-valve	Mitral and aortic bioprosthesis dysfunction, in symptomatic patients who have high surgical risk or are inoperable (following evaluation by the Heart Team)
Transcatheter treatment – TAVI and percutaneous mitral repair	Severe AS and severe primary MR, in patients with symptoms and/or prognostic factors, when there is a high surgical risk or contraindication to surgery (following evaluation by the Heart Team)

MR: mitral regurgitation; MS: mitral stenosis; TAVI: transcatheter aortic valve implantation.

**Table 59:** Multivalvular disease: Recommendations^1^^,^^2^^,^^163^^–^^165^

Intervention	Clinical condition	SBC	AHA	ESC
Percutaneous balloon mitral valvuloplasty	Severe symptomatic MS with favorable anatomy and aortic moderate lesion	I A	–	–
Surgical treatment/valve replacement	Symptomatic multivalvular disease	I B	I B	I B
	Multivalvular disease with prognostic factors	IIa C	–	–
	Treatment of moderate valve lesion concomitant to treatment of severe valvular disease or other cardiac or ascending aorta surgery	I C	I C	I C
Transcatheter treatment – valve-in-valve	Mitral and aortic biological prosthesis dysfunction with symptoms and high surgical risk	IIb C	–	–
	Mitral and aortic biological prosthesis dysfunction with prognostic factors and high surgical risk	IIb C	–	–
Transcatheter treatment – TAVI and percutaneous mitral repair	Severe AS and severe primary MR with symptoms and high surgical risk	IIb C	–	–
	Severe AS and severe primary MR with prognostic factors and high surgical risk	IIb C	–	–

AHA: American Heart Association; AS: aortic stenosis; MR: mitral regurgitation; MS: mitral stenosis; ESC: European Society of Cardiology; SBC: Sociedade Brasileira de Cardiologia (Brazilian Society of Cardiology); TAVI: transcatheter aortic valve implantation.

## 14. Evaluation of Coronary Artery Disease

Before cardiac valve surgery or transcatheter intervention for VHD, patients must undergo evaluation of coronary artery disease with coronary angiography if they meet any of the following criteria: 40 years of age or older, suspected coronary artery disease (risk factors for atherosclerosis [diabetes, dyslipidemia, arterial hypertension, and others], prior events, or angina), LV dysfunction, or in order to evaluate the etiology in secondary MR.^166^^–^^168^ Coronary tomography angiography may be used in patients with low or intermediate probability of coronary artery disease. If coronary tomography shows significant or unclear lesions, the patient should undergo coronary angiography (Table 60).^169^^–^^171^

**Table 60:** Intervention in coronary artery disease concomitant to valve Intervention: Recommendations^1^^,^^2^^,^^166^^–^^171^

Intervention	Clinical condition	SBC	AHA	ESC
Myocardial revascularization surgery	Indication of valve surgery and coronary lesion ≥ 70%	I C	IIa C	I C
Coronary angioplasty	Indication of transcatheter valve intervention and coronary lesion ≥ 70% in a proximal segment	IIa C	IIa C	IIa C

AHA: American Heart Association; ESC: European Society of Cardiology; SBC: Sociedade Brasileira de Cardiologia (Brazilian Society of Cardiology).

## 15. Anticoagulation

The two prognostic factors with the greatest impact on the natural history of valve disease are hemodynamic repercussions and thromboembolism. Stroke is the most clinically significant thromboembolic event, affecting up to 20% of individuals with AF associated with valve disease. The CHA_2_DS_2_-VASc score is recommended for decision making regarding anticoagulation, except for patients with rheumatic MS or those with mechanical prostheses. The criteria for anticoagulation are the same for patients with paroxysmal, persistent, or permanent AF. The main indications for anticoagulation are described in Table 61.

**Table 61:** Indications for oral anticoagulation^1^^,^^2^^,^ ^172^^–^^183^

Clinical condition	Medication	SBC	AHA	ESC
Native valve				
MS with AF and/or LA thrombus*	Warfarin	I B	I B	I B
	DOACs	III C	III C	III C
	ASA	IIb B	–	–
Other valvular heart diseases with AF	Warfarin	I B	I C	I B
	DOACs	IIa C	IIa C	IIa B
	ASA	IIb B	–	–
Previous embolic event without AF	Warfarin	I B	I B	–
	DOACs	III C	–	–
	ASA	IIb C	–	–
**Biological prosthesis**				
AF	Warfarin	I B	I B	I C
	DOACs	IIb B	–	–
	ASA	IIb C	–	–
Sinus rhythm – mitral bioprosthesis (first 3 to 6 months)	Warfarin	IIb	IIa B	IIa C
	DOACs	III C	–	–
	ASA	IIb	–	–
Sinus rhythm – aortic bioprosthesis (first 3 to 6 months)	Warfarin	IIb B	IIa B	IIb C
	DOACs	III C	–	–
	ASA	IIb B	–	IIa C
**TAVI**				
AF	Warfarin	I B	–	–
	DOACs	IIb C	–	–
	ASA + clopidogrel	III B	–	–
	ASA	III C	–	–
Sinus rhythm	Warfarin	III B	IIb B (3 months)	IIb C (3 months)
	DOACs	III B	–	–
	ASA or clopidogrel, indefinitely	IIa B	–	IIb C
	ASA + clopidogrel, 3 to 6 months	IIb B	IIb C	IIa C
Sinus rhythm + angioplasty with stent (chronic coronary artery disease)	ASA + clopidogrel up to 12 months, according to stent type	IIa C	IIb	–
AF + angioplasty with stent (chronic coronary artery disease)	DOAC + clopidogrel	IIa C	–	–
	Warfarin + ASA + clopidogrel 1 month, followed by warfarin + clopidogrel up to 12 months	IIb C	–	–
**Mechanical prosthesis**				
	Warfarin	I B	I A	I B
	DOACs	III B	III B	III B
	Warfarin + routine ASA	III C	IIa B	–
	Warfarin + ASA after a thromboembolic event within therapeutic INR	IIa B	–	IIa C

* Consider anticoagulation with warfarin in individuals with MS and episodes of sustained atrial tachycardia or enlarged LA (≥ 50 mm anteroposterior diameter or ≥ 50 ml/m² LA volume) and spontaneous contrast. AF: atrial fibrillation; AHA: American Heart Association; ASA: acetylsalicylic acid; DOACs: direct oral anticoagulants; ESC: European Society of Cardiology; INR: international normalized ratio; LA: left atrium; SBC: Sociedade Brasileira de Cardiologia (Brazilian Society of Cardiology); TAVI: transcatheter aortic valve implantation.

Oral anticoagulation, as a means of preventing thromboembolic events in patients with valve disease, is still predominantly carried out with vitamin K antagonists (VKA); warfarin currently represents this class of drugs in Brazil. It is a safe strategy to start warfarin at a dose of 5 mg/day in individuals under 65 years of age and 2.5 mg/day in individuals over 65 years. Prothrombin time should be measured on the third day to evaluate hyper-responsiveness to the medication and again on the fifth day, after which the dose proceeds to be adjusted. During this phase, exams should be carried out at 5-day intervals until the therapeutic level has been reached. The international normalized ratio (INR) should remain between 2.0 and 3.0, except in patients who have mechanical prostheses in the mitral position, aortic mechanical prosthesis associated with AF, hypercoagulable states, and cardioembolic events while INR is between 2.0 and 3.0. In these cases, the target becomes 2.5 to 3.5. INR control is usually performed on a monthly basis; in patients whose doses have been sTable for a long time and who have not been exposed to any new factors that interact with warfarin (Table 62), the control may be done every two months. In the event that INR is above the target, a new exam should be performed on an earlier basis, in 1 to 2 weeks. Dose adjustments should be, on average, 10% to 15% of the weekly dose, and it is necessary to investigate which factors caused the oscillation in INR. Monitoring of prothrombin time with point of care devices provides quick and reliable information; its availability, however, is still limited due to the high cost of the device and the strips.

**Table 62:** Warfarin dose adjustments

INR value	Dose adjustment
≤ 1.5	Increase weekly dose by 15%
1.51 – 1.99	Increase weekly dose by 10%
2 – 3*	Maintain dose
3.01 – 4.0	Reduce weekly dose by 15%
4.01 – 4.99	Suspend 1 dose and reduce weekly dose by 10%
5.0 – 8.99	Suspend warfarin until INR is 2 to 3 then start again with weekly dose reduced by 15%
≥ 9.00	Hospitalization, suspend warfarin for an average of 4 days, prescribe vitamin K at a dose of 1 to 2.5 mg orally, repeating 24 to 48 hours later if INR does not decrease to < 5.0, and restart anticoagulation once INR is close to target value (below 4)

* Consider maintaining the weekly dose of warfarin with INR up to 3.5, provided that the medication has not been initiated recently, and perform new measurement in 1 to 2 weeks. In case of the therapeutic INR goal is between 2.5 and 3.5, dose adjustments should occur adding 0.5 to the above values, with the exception of INR ≥ 9.0. INR: international normalized ratio.

It is known that greater time in therapeutic range (TTR) is associated with lower risk of thromboembolic events and bleeding. In a study including 119 patients with mitral valve disease and AF, 78.2% of individuals had INR < 2.0 at the time of the thromboembolic event. For INR values < 1.7 the likelihood doubled, and it tripled for values < 1.5. The difficulties of managing VKA are result of the wide variability in individual dose and interactions with foods and medications, in addition to the need for frequent monitoring. Patients should be advised to avoid alcohol consumption and to maintain a balanced diet, especially in relation to foods that are rich in vitamin K, such as greens and vegetables. These foods should not be excluded from the dietary routine.

Over the past years, the role of direct oral anticoagulants (DOACs) has progressively increased. Dosages of medications available in Brazil can be found in Table 63. Multiple clinical trials involving patients with VHD are underway. Most of the current information is from analyses of subgroups of the main studies on DOACs, as well as retrospective cohort studies.

**Table 63:** Dose of direct oral anticoagulants for prophylaxis of thromboembolic events in atrial fibrillation^177^^–^^180^

Anticoagulant	Usual dose	Dose adjustment	Contraindications
Dabigatran	150 mg twice daily	≥ 80 years of age and/or high risk of bleeding: 110 mg twice daily	Creatinine clearance < 30 mL/min, concomitant use of ketoconazole
Rivaroxaban	20 mg once daily	15 mg once daily if creatinine clearance is < 50 mg/dL	Creatinine clearance < 15 mL/min, hepatic disease associated with coagulopathy
Apixaban	5 mg twice daily	2.5 mg twice daily in patients with at least 2 of the following criteria: age ≥ 80 years, body weight ≤ 60 kg, or serum creatinine ≥ 1.5 mg/dL	Creatinine clearance < 15 mL/min, hepatic disease associated with coagulopathy
Edoxaban	60 mg once daily	30 mg once daily	Creatinine clearance > 95 mL/min or < 15 mL/min

In patients with mechanical prostheses, pre-clinical trials involving animals have suggested that the use of DOACs could be as safe as warfarin. However, the clinical Dabigatran versus Warfarin in Patients with Mechanical Heart Valves (RE-ALIGN) study, which compared dabigatran and warfarin, was prematurely terminated due to greater occurrence of the combined outcome of stroke, transient ischemic attack, systemic embolism, myocardial infarction, and death (9% versus 5%; hazard ratio 1.94, 95% CI 0.64 to 5.86) and bleeding (27% versus 12%, p < 0.05) in the first group. The study included 252 patients, and it used dabigatran at doses of 150, 220, and 300 mg, administered every 12 hours, according to creatinine clearance, with dose adjustments for serum level above 50 ng/mL. For this reason, we do not indicate the use of DOACs in patients with mechanical prostheses.^176^

Although the large clinical trials that have validated the use of DOACs in AF excluded individuals with severe MS and mechanical valve prostheses, these studies did include individuals with other VHD. In the Apixaban versus Warfarin in Patients with Atrial Fibrillation (ARISTOTLE) study, 26.4% of participants had moderate or severe VHD; in the Dabigatran versus Warfarin in Patients with Atrial Fibrillation (RE-LY) study, 21.8%; in the Rivaroxaban versus Warfarin in Nonvalvular Atrial Fibrillation (ROCKET AF) study, 14.1%; and, finally, in the Edoxaban versus Warfarin in Patients with Atrial Fibrillation (ENGAGE AF) study, 13%. Subanalyses of these studies suggest the efficacy of DOACs in comparison with warfarin in individuals with AF and valve disease, excluding patients with mechanical prostheses and severe MS. The ARISTOTLE and ENGAGE-AF studies included individuals with bioprostheses. ^177^^–^^180^

Notwithstanding the negative results in individuals with mechanical valve prostheses, dabigatran has been shown to be effective in preventing intracardiac thrombus formation in individuals with aortic and/or mitral biological prosthesis in a Brazilian single-center study, Dabigatran Versus Warfarin After Bioprosthesis Valve Replacement for the Management of Atrial Fibrillation Postoperatively (DAWA).^175^

A South Korean cohort with 2,230 patients evaluated individuals with AF and MS of different etiologies and degrees of anatomical severity, comparing off-label use of DOACs in relation to warfarin. Ischemic events occurred in 2.22% yearly in the DOAC group versus 4.19% yearly in the warfarin group (hazard ratio 0.28; 95% CI 0.18 to 0.45), and intracranial bleeding occurred in 0.49% in the DOAC group versus 0.93% in the warfarin group (hazard ratio 0.53; 95% CI 0.22 to 1.26). This study reinforces the hypothesis of the efficacy of DOACs in MS. Attention should be paid to the fact that TTR of INR was not evaluated in this cohort. ^181^ In a multi-center observational study, Korean patients had only 31% of INR values within the therapeutic target.

The first antithrombotic regimen adopted for individuals undergoing TAVI in sinus rhythm was dual antiplatelet therapy with ASA and clopidogrel for 6 months, inferring from experience with stents and based on the expected period for endothelialization of the prosthesis to occur. In a meta-analysis of three recent small clinical trials, antiplatelet therapy with ASA or clopidogrel alone did not show an increase in 30-day mortality (odds ratio 5.2 versus 3.2%, p = 0.447) or ischemic events (3.8 versus 3.8%, p = 0.999), when compared with dual antiplatelet therapy; furthermore, there was a higher chance of bleeding in the dual antiplatelet therapy group (odds ratio 2.24; 95% CI 1.12 to 4.46; p = 0.022). ^173^

There is evidence, from transesophageal echocardiogram and computed tomography angiography, of the occurrence of thickening of the leaflets after TAVI in up to 13% of patients, which may correspond to the formation of thrombi, and it has been associated with increased incidence of transient ischemic attack and stroke.^174^ Observational cohort studies where individuals received VKA or DOACs have indicated that the use of these medications could be safe for prevention of events. However, the recently published multi-center Global Study Comparing a Rivaroxaban-based Antithrombotic Strategy to an Antiplatelet-based Strategy After Transcatheter Aortic Valve Replacement to Optimize Clinical Outcomes (GALILEO) study, which included 1,644 patients without established indication for dual antiplatelet therapy or anticoagulation, comparing the use of rivaroxaban 10 mg/day (associated with ASA 75 – 100 mg/day during the first 3 months) versus ASA 75 – 100 mg (associated with clopidogrel 75 mg/day during the first 3 months). The study was prematurely terminated due to greater occurrence of thrombotic events (9.8 and 7.2 per 100 person-years; hazard ratio 1.35, 95% CI 1.01 to 1.81; p = 0.04), bleeding (4.3 and 2.8 per 100 person-years; hazard ratio 1.5, 95% CI 0.95 to 2.37; p = 0.08), and death (5.8 and 3.4 per 100 person-years; hazard ratio 1.69, 95% CI 1.13 to 2.53) in the rivaroxaban group. ^172^

### 15.1. Surgical Procedures

In surgical procedures with low risk of bleeding, where hemostasis is possible, such as cataract surgery, glaucoma surgery, small dermatological surgeries, dental or gum surgeries, periodontal scraping and simultaneous extraction of up to 3 teeth, it is suggested to maintain oral anticoagulation. In the case of warfarin, INR should be within the therapeutic range, as measured 24 to 48 hours before the procedure. In the case of DOACs, ideally, the procedure should not be performed during the hours following use of these medications, in order to avoid their peak plasma concentrations.

With respect to procedures that imply higher risk of bleeding due to the size of the surgery or difficulty in achieving hemostasis, heparin bridging is indicated in individuals using VKA. These procedures include coronary angiography, endoscopy or colonoscopy with polypectomy, postectomy, vasectomy, internal organ biopsies, and larger surgeries. In these cases, warfarin should be suspended during the 5 days preceding the procedure, starting heparin 3 days before the procedure. In the case of low molecular weight heparin, the last dose should be administered 24 hours before the procedure, and unfractionated heparin should be suspended 4 to 6 hours before the surgery. Heparin is generally reintroduced 12 hours later, provided that hemostasis is adequate. Warfarin is, generally, restarted on the following day. INR should be measured in 5 days, and heparin should be suspended as soon as the therapeutic target has been reached. In emergency surgeries, 50 IU/kg prothrombinic complex should, ideally, be administered intravenously.

The rapid onset of action of DOACs (2 to 4 hours) and their short elimination half-life dispense with the need of using a heparin bridge. For elective procedures with low risk of bleeding, suspension is recommended 24 hours before surgery, and, in cases with elevated risk of bleeding or sites with difficult hemostasis, the recommendation is to suspend 48 hours before. In emergency surgery, use of the antidote idarucizumab is recommended in individuals using dabigatran, with a total dose of 5 g endovenously (two 2.5-g aliquots). Andexanet alfa (Andexxa), an antidote to factor Xa inhibitors, is not yet available in Brazil.

## 16. Prosthetic Valve Thrombosis

Prosthetic valve thrombosis is an uncommon event; it is more frequent in mechanical prostheses, especially in the mitral position, and it is associated with high morbimortality. It may be asymptomatic or it may manifest with heart failure syndrome, low output, and even death. Diagnosis and suspicion are usually made after transthoracic echocardiogram, and they may be confirmed by the transesophageal method (Tables 64, 65, and 66).

**Table 64:** Step 1: Diagnosis of prosthetic valve thrombosis

	Characteristics of prosthesis thrombosis
Clinical evaluation	Symptoms and signs suggestive of acute or exacerbated heart failure (dyspnea, chest pain, low output, or syncope) Murmur compatible with stenotic valvular heart disease Muffled clicking sound Possibility of ineffective anticoagulation (INR outside therapeutic range)
Electrocardiogram	Compatible with the baseline disease that was the reason for valve surgery Rarely shows acute alteration
Chest radiography	Compatible with the baseline disease that was the reason for valve surgery Rarely shows acute alteration of the cardiac silhouette Pulmonary congestion may be present
Echocardiogram	Key test for diagnosis Ideally transesophageal Documentation of thrombus adhering to the prosthesis, identification of location and size of the thrombosis
Hemodynamic study (fluoroscopy)	Inadequate mobility of one or more leaflets of the mechanical prosthesis

INR: international normalized ratio.

**Table 65:** Step 2: Evaluation of prosthetic valve thrombosis etiology

	Etiological characteristics
Ineffective anticoagulation	Interruption of anticoagulation Drug/behavioral interaction INR below therapeutic target

INR: international normalized ratio.

**Table 66:** Step 3: Evaluation of prosthetic valve thrombosis symptoms

	Symptoms
Dyspnea	Main symptom Distinguish between mild worsening (NYHA FC I) and more evident symptoms (NYHA FC II to IV)
Precordial pain	Possibility of coronary embolism
Low output or syncope	Indicative of a severe obstruction

FC: functional class.

The main prognostic factor of thrombosis is thrombus size, due to the risk of embolism and valve obstruction (Table 67).

**Table 67:** Step 4: Evaluation of prosthetic valve thrombosis prognostic factors

	Prognostic factors
High risk of embolization associated with thrombolysis	Thrombus > 8 mm Mobile thrombus (pedunculated)
Pulmonary hypertension	Resting SPAP ≥ 50 mmHg More frequent when there is associated MS Clinically – symptoms of right heart failure Related to increased surgical risk
Recent onset AF	Related to significant LA remodeling

AF: atrial fibrillation; LA: left atrium; MS: mitral stenosis; SPAP: systolic pulmonary artery pressure.

The recommendations of international guidelines are heterogeneous in relation to treatment, and there is a lack of randomized studies in this area (Tables 68 and 69). In prosthesis thrombosis without significant hemodynamic repercussion (NYHA FC I and II), without valve obstruction on complementary tests, oral anticoagulation and outpatient monitoring with imaging are indicated. In the event of a large (especially greater than 8 mm) and/or mobile thrombus, which has an elevated risk of embolization, hospitalization with parenteral anticoagulation is indicated. In the event that the thrombus is not reduced on imaging tests, performed every 5 to 7 days, fibrinolysis and/or surgery may be considered.^184^^,^^185^

**Table 68:** Step 5: Type of prosthetic valve thrombosis intervention^184^^,^^185^

Type	Considerations
Thrombolysis	Priority therapy rTPA 10 mg (bolus), followed by 90 mg in 2 hours OR Streptokinase 500,000 IU in 20 minutes, followed by 1,500,000 IU in 10 hours
Valve surgery	Reserved for cases with high risk of hemorrhagic or embolic complications associated with thrombolysis

rTPA: recombinant tissue plasminogen activator.

**Table 69:** Prosthesis thrombosis: Recommendations^1^^,^^2^^,^^184^^,^^185^

Intervention	Clinical condition	SBC	AHA	ESC
Thrombolysis	Valve thrombosis in a right chamber	IIa B	IIa B	–
	Small thrombus (< 0.8 cm²), NYHA FC I to III, left chambers if the thrombus persists after parenteral anticoagulation	IIa B	IIa B	–
Valve surgery	NYHA FC IV, left chambers	IB	I B	I C
	Mobile or large (> 0.8 cm²) thrombus, left chambers	IIa C	IIa C	IIa C (thrombus > 10 mm)

AHA: American Heart Association; ESC: European Society of Cardiology; FC: functional class; NYHA: New York Heart Association; SBC: Sociedade Brasileira de Cardiologia (Brazilian Society of Cardiology).

In cases where there is a more significant hemodynamic impairment (NYHA FC III and IV), fibrinolytic therapy or valve surgery is usually indicated. Recently, there has been a trend to prioritize fibrinolysis over surgery, based on data from a meta-analysis of 48 studies. When deciding on these two strategies, discussion with the Heart Team is recommended, and the risks of fibrinolysis (preferred procedure) and surgery should be weighed individually. The following factors make fibrinolysis favorable: high surgical risk, low risk of bleeding, involvement of the right valves, first episode of valve thrombosis, and thrombus smaller than 1 cm². If there is hemodynamic instability, the treatment of choice is surgery, and fibrinolysis may be considered in individuals with elevated surgical risk. The following factors make surgical procedure favorable: contraindication to fibrinolysis, high risk of bleeding, low surgical risk, suspicion of pannus associated with thrombosis, and need for other concomitant cardiac surgical procedures (for example, myocardial revascularization).^184^^,^^185^

## 17. Prophylaxis of Rheumatic Fever

RF and consequent chronic rheumatic heart disease remain the most important cause of acquired VHD in Brazil. Rheumatic disease is one of the most costly diseases for the Brazilian Unified Health System and the community in general, because it affects very young individuals, and it frequently leads to multiple hospitalizations and surgeries. It continues to be the main cause of acquired VHD in Brazil. The goal of decreasing its incidence is of the utmost importance, considering that it is certainly the most easily prevenTable cardiovascular disease.

### 17.1. Primary Prophylaxis of Rheumatic Fever

In order to decrease the incidence of RF, the measure with the greatest impact is primary prophylaxis, preventing susceptible individuals from contracting the disease (Tables 70 and 71). We have recently encountered serious difficulties in carrying out primary prophylaxis; supplies of benzathine penicillin G are unreliable, with frequent shortages of the medication. Furthermore, restrictions on locations where the medication may be administered, due to concerns regarding allergic reactions and lack of familiarity with intramuscular application on the part of primary healthcare professionals, have made it increasingly difficult to perform primary prophylaxis via the intramuscular route. This fact will certainly contribute to increased incidence of the disease in the coming years.

**Table 70:** Medications and posology indicated for streptococcal pharyngotonsillitis – primary prophylaxis of rheumatic fever^186^^–^^193^

Medication		Dose	Route of administration / Duration	Comments
Penicillins and derivatives				
	Benzathine benzylpenicillin	600,000 IU up to 25 kg, 1,200,000 IU over 25 kg	Intramuscular Single dose	Medication of choice: single dose, high efficacy and low cost
	Amoxicillin	50 mg/kg for children and 1.5g daily for adults, divided in 2 to 3 doses	Oral 10 days	Low adherence to complete treatment
	Phenoxymethylpenicillin	250 mg 2 to 3 times daily up to 25 kg, 500 mg 3 times daily > 25 kg	Oral 10 days	Low adherence to complete treatment
For patients who have allergy to penicillin				
	Clindamycin	20 mg/kg divided 3 times daily, adults: 300 to 600 mg 3 times daily	Oral 10 days	Frequent gastrointestinal intolerance
	Azithromycin	12 mg/kg in a single daily dose. For adults, 500 mg once daily	Oral 5 days	The only oral antibiotic therapy that may eradicate streptococcus in less than 10 days
	Clarithromycin	15 mg/kg twice daily or, for adults, 250 mg twice daily	Oral 10 days	

**Table 71:** Recommendations for primary prophylaxis of rheumatic fever^186^^–^^193^

**Class I** – Benzathine benzylpenicillin in patients with streptococcal tonsillitis – Benzathine benzylpenicillin in patients with suspected streptococcal tonsillitis, even without diagnostic confirmation – Oral antibiotic therapy in patients with streptococcal tonsillitis who are allergic to penicillin
**Class IIa** – Use of oral antibiotics for treatment of streptococcal pharyngotonsillitis in patients who are not allergic to penicillin – Rapid tests to detect streptococci in the oropharynx in order to make the decision regarding treatment with penicillin.
**Class III** – Oropharynx culture in patients with suspected tonsillitis in order to make the decision regarding treatment with penicillin.

Oral therapies should not be used routinely, because 10 days of therapy are generally necessary in order to completely eradicate streptococci from the oropharynx. For this reason, there is a very high risk of non-adherence to the complete treatment, placing patients at the risk of developing a rheumatic attack. Treatments based on 5 days of azithromycin have been proposed, but there are still no clinical studies validating its use in pharyngotonsillitis.^186^^–^^193^

### 17.2. Secondary Prophylaxis of Rheumatic Fever

For patients who have already been diagnosed with RF, secondary prophylaxis is indicated in order to prevent new attacks of acute RF (Tables 72 and 73). The drug of choice is benzathine benzylpenicillin, at the same doses of 600,000 IU for children weighing up to 27 kg and 1,200,000 IU above this weight, at a maximum interval of three weeks. Monthly applications of benzathine penicillin do not promote adequate protection in patients with rheumatic disease in countries with high endemicity of the disease, like Brazil.^194^^–^^198^ For patients who are allergic to penicillin, sulfadiazine is indicated at a dose of 1 g daily, and it is necessary to control possible leukopenic conditions.

**Table 72:** Secondary prophylaxis of rheumatic fever: Recommended medications and posology^194^^–^^200^

Medication		Dose and frequency	Recurrence / Notes
	Benzathine benzylpenicillin G	< 25 kg – 600,000 IU > 25 kg – 1,200,000 IU Every 15 days during the first two years after the attack Every 21 days during subsequent years	Recurrence of 0.3% yearly Medication of choice
	Phenoxymethylpenicillin	250 mg orally twice daily	Recurrence of 5%/year Should not be used as an alternative to benzathine penicillin G
For patients who have allergy to penicillin	Sulfadiazine	< 25 kg – 500 mg daily > 25 kg – 1 g daily	Recurrence of 1.3% yearly May be used until penicillin desensitization is concluded
For patients who have allergy to penicillin and sulfadiazine	Erythromycin	250 mg twice daily	Empirical regimen of prophylaxis, has not been the subject of studies on secondary prophylaxis of RF – should only be used in exceptional cases

RF: rheumatic fever.

**Table 73:** Recommendations for secondary prophylaxis of rheumatic fever^194^^–^^200^

**Class I** – Benzathine benzylpenicillin G for secondary prophylaxis of RF, every 15 days during the first two years after the attack and every 21 days during the following years. – Use of benzathine benzylpenicillin G until 18 years of age, or 5 years after the last attack in patients with RF without carditis. – Use of benzathine benzylpenicillin G until 25 years of age, or 10 years after the last attack in patients with RF and carditis, without cardiac sequelae or mild sequelae, provided that there are no stenotic lesions. – Use of benzathine benzylpenicillin G until 40 years of age in patients with RF and carditis, with severe sequelae or cardiac surgery to correct valvular heart disease. – Use of benzathine benzylpenicillin G after 40 years of age in patients who are occupationally exposed to streptococci. – Sulfadiazine for antibiotic prophylaxis of RF in patients who are allergic to penicillin
**Class IIa** – Use of oral antibiotic prophylaxis for patients with RF who are not allergic to penicillin
**Class IIb** – Use of erythromycin for antibiotic prophylaxis for patients with RF who are allergic to penicillin and sulfa medications
**Class III** – Suspension of antibiotic prophylaxis for RF after cardiac surgery with implantation of valve prosthesis, even when other valves do not have apparent lesions.

RF: rheumatic fever.

Considering the recent shortage of benzathine penicillin G, the alternative is sulfadiazine, which is frequently available for rheumatologic diseases in the public health system and is listed in high-cost medication regimens. We must also remember that only benzathine penicillin G and sulfadiazine have proven efficacy for secondary prophylaxis of RF, based on controlled studies.^199^^-^^200^

### 17.3. Criteria for Suspending Prophylaxis (Table 74)

**Table 74:** Duration of secondary prophylaxis of rheumatic fever

Category	Duration
RF without carditis: clinical of pure arthritis or chorea	Until 18 years of age or 5 years after the last attack of RF, whichever is longer
RF with carditis, without sequelae or with very mild valvular sequelae (excluding stenotic lesions, even if they are very mild)	Until 25 years of age or 10 years after the last attack
RF with carditis and severe sequelae; patients undergoing cardiac surgery	Until 40 years of age, at least; lifelong if occupationally exposed

RF: rheumatic fever.

– Patients without cardiac involvement, with only joint manifestation or “pure” chorea – suspend at 18 years of age or 5 years after the last rheumatic attack;
– Patients with carditis during the acute attack who do not have late sequelae or who have very mild sequelae – suspend at 25 years of age or 10 years after the last rheumatic attack;
– In patients whose prophylaxis is suspended and symptoms recur, prophylaxis should be maintained for 5 more years.
– Patients with even mild cardiac involvement should receive prolonged prophylaxis, preferably lifelong; when this is not possible, until the fourth decade life. When deciding to suspend the prophylaxis, we must always investigate occupational exposure to sources of streptococci.

## 18. Prophylaxis of Infective Endocarditis in VHD

IE is a severe complication of VHD, and it is frequently fatal. For this reason, when prophylaxis is possible, it should be applied. For this purpose, several antibiotic regimens have been utilized, with little evidence from controlled studies, mainly due to the difficulty of conducting large controlled studies with medications that are already in the public domain.

Streptococci are part of the normal oropharynx and gastrointestinal tract flora, and they cause at least 50% of acquired IE cases in the Brazilian community. Bacteremia due to viridans streptococci has been demonstrated in up to 61% of patients following tooth extraction and periodontal surgery (36% to 88%), and experimental studies in animals have shown that antibiotic prophylaxis was capable of avoiding IE due to viridans streptococci and enterococci.^201^^,^^202^

More recently, it has been observed that spontaneous bacteremia, especially originating in the teeth and gums, occurs in everyday situations. Thus, ordinary routine activities, such as tooth brushing (0% to 50%), use of dental floss (20% to 68%), use of toothpicks, and even chewing during meals (7% to 51%), are associated with bacteremia. In this manner, the burden of spontaneous bacteremia, not caused by dental intervention, would be higher than that caused by dental treatments. A theoretical study of cumulative bacteremia, lasting approximately one year, calculated that everyday bacteremia is six times greater than bacteremia caused by isolated tooth extraction. Considering that dental prophylaxis indications recommend two annual visits to the dentist, everyday activities have a greater impact on the generation of bacteremia than dental intervention itself. Recent epidemiological studies have not shown a relation between dental treatment two weeks before and episodes of IE.^203^^–^^208^

For this reason, maintenance of optimal oral health in patients with VHD is more important than prophylaxis before dental procedures. Patients with good oral health have lower chances of bacteremia from everyday activities. We must, thus, focus more on non-pharmacological prevention than on pharmacological prophylaxis. Part of non-pharmacological prophylaxis of IE is to reinforce, during all consultations, the need to maintain excellent oral health and to increase the frequency of dental consultations, from two (recommendation for the general population) to four times a year. It is necessary to underline that many of the dental conditions that most frequently cause IE are oligosymptomatic, such as gingivitis and periapical endodontic lesions.^209^

For patients undergoing dental interventions, there is growing evidence that antibiotic prophylaxis prevents only a very small number of cases of IE. There is, however, recent evidence that completely abolishing antibiotic prophylaxis could lead to increased incidence of IE. The British National Institute for Health and Care Excellence (NICE) proposed that prophylaxis of IE should not be applied on any occasion.^210^ As a consequence, a decrease was observed in the prescription of antibiotic prophylaxis before dental treatments, followed by an increase in the number of cases of IE.^211^ We thus have empirical evidence that completely abolishing antibiotic prophylaxis could lead to an increase in cases of IE. We accordingly recommend maintaining antibiotic prophylaxis before dental, gastrointestinal, and genitourinary procedures.

All patients with moderate to severe VHD, whether of rheumatic or degenerative etiology, and patients with prosthetic valves should receive non-pharmacological and pharmacological prophylaxis for IE, once all patients with IE have high morbimortality.

### 18.1. Non-pharmacological Prophylaxis of Infective Endocarditis

Non-pharmacological prophylaxis of IE may be more effective than pharmacological prophylaxis, as it acts toward primary prevention of proven sources of bacteremia (Table 75). As priority measures for patients with VHD, we highlight maintaining excellent oral health and avoiding invasive body art procedures, such as piercings and tattoos.

**Table 75:** Non-pharmacological prophylaxis of infective endocarditis

Recommendation	Class of recommendation	Level of evidence
During medical consultations, reinforce the need to maintain good oral health and appropriate hygiene habits	I	C
Quarterly dental consultations	I	C
Tattoo	III	C
Skin piercings	III	C
Piercings of the tongue and mucous membranes	III	C

Body art (procedures such as tattoos and piercings) should be contraindicated. Piercings lead to the formation of a tract that needs to be epithelialized, and until that process is complete, it is a source of continuous bacteremia, with many reports of IE related to piercings in the literature, some of them with fatal outcomes. It is important for patients to be informed regarding the risks of this procedure, in the same manner that physicians should always cover this issue when treating patients who have or intend to have body art.^212^

### 18.2. Prophylaxis of Infective Endocarditis for Dental Procedures (Tables 76, 77, and 78)

**Table 76:** Indications of prophylaxis for dental procedures

High likelihood of significant bacteremia	Without high likelihood of significant bacteremia
Procedures that involve manipulation of gum or periodontal tissue or perforation of oral mucosa.	Local anesthesia in non-infected tissue
	Dental radiography
	Placement or removal of orthodontic appliances
	Adjustment of orthodontic appliances
	Placement of parts in orthodontic appliances
	Natural loss of deciduous teeth
	Bleeding due to trauma of the oral mucosa or the lips

**Table 77:** Antibiotic prophylaxis of IE in VHD

Indication	Recommendation	Level of evidence
Patients with moderate and severe valvular heart disease, or patients with prosthetic valves, who will undergo dental procedures with high likelihood of significant bacteremia.	I	C
Patients with an elevated risk of severe infective endocarditis* who will undergo genitourinary or gastrointestinal procedures associated with lesion of the mucosa.	IIa	C
Patients with elevated risk of severe infective endocarditis* who will undergo esophagus or respiratory tract procedures associated with lesion of the mucosa.	IIa	C
Patients with MVP without regurgitation, patients after myocardial revascularization surgery or stent placement, patients with functional heart murmur, patients with pacemaker or defibrillator, patients with Kawasaki disease or RF without valvular dysfunction, who will undergo dental, respiratory tract, genitourinary, or gastrointestinal procedures.	III	C
Patients undergoing procedures that do not involve risk of bacteremia.	III	C

* Elevated risk of severe IE: prosthetic heart valve; prior IE; congenital heart disease that is unrepaired, partially corrected, or corrected with prosthetic material; heart transplant with VHD. MVP: mitral valve prolapse.

**Table 78:** Regimens for prophylaxis of infective endocarditis before dental procedures

Route of administration	Medication	Single dose 1 hour before the procedure	
		Children	Adults
Oral	Amoxicillin	50 mg/kg	2 g
Oral (penicillin allergy)	Clindamycin	20 mg/kg	600 mg
	Azithromycin or clarithromycin	15 mg/kg	500 mg
Parenteral (endovenous or intramuscular)	Ampicillin	50 mg/kg	2 g
	Cefazolin or ceftriaxone	50 mg/kg	1 g
Parenteral (endovenous or intramuscular) (penicillin allergy)	Clindamycin	20 mg/kg	600 mg

The antibiotic should be administered one hour before the procedure. The regimen used should prevent bacteremia due to streptococci viridans, whenever tissue from the gums or the periapical region of the tooth is to be manipulated. The antibiotic of choice, if the patient is not allergic, is amoxicillin, due to its adequate absorption and to the susceptibility of the infectious agent. However, resistance to the antibiotic has been reported in several strains of the microorganism. For patients who are allergic to penicillin, the following may be used: clindamycin, azithromycin, or clarithromycin.

### 18.3. Prophylaxis of Infective Endocarditis for Respiratory Tract Procedures

Patients who will undergo incision or biopsy of the mucosa of the respiratory tract, such as otorhinolaryngological surgery, should receive antibiotic regimens similar to those used for conditions affecting the mouth.

### 18.4. Prophylaxis of Infective Endocarditis for Genitourinary or Gastrointestinal Tract Procedures

Enterococci are part of the the gastrointestinal tract flora, and they can cause IE. Thus, considering the lack of adequate scientific evidence, American and European guidelines no longer indicate antibiotic prophylaxis before interventions in these locations.^213^^,^^214^ Though, considering the severity of an eventual occurrence of IE by these sources, in the current document, we have chosen to consider prophylaxis for patients with high risk of severe IE who will undergo genitourinary or gastrointestinal procedures associated with mucosal injury. (Table 79).^215^ In the presence of infections that have installed in the genitourinary and gastrointestinal tracts, treatment should include antibiotics that act against enterococcus.

**Table 79:** Parenteral antibiotic prophylaxis for procedures in the gastrointestinal and genitourinary tracts

Route of administration	Medication	Single dose 1 hour before the procedure		
		Children		Adults
Parenteral (intravenous)	Ampicillin +	50 mg/kg		2 g
	Gentamicin	1.5 mg/kg		
Parenteral (intravenous) - penicillin allergy	Vancomycin +	20 mg/kg		1 g
	Gentamicin	1.5 mg/kg		

## 19. Pregnancy, Family Planning, and Contraception

### 19.1. Pre-Pregnancy Counseling

Risk stratification of valve diseases during pregnancy planning must be based on anatomical diagnosis of the valve lesion in order to classify the risks of pregnancy as high, intermediate, or accepTable (Table 80).

**Table 80:** Classification of risks of valve diseases to pregnancy

High risk	Intermediate risk	AccepTable risk
Severe MS	Biological prosthesis with moderate dysfunction	Mild valve disease
Severe AS Stenotic/calcified biological prosthesis Mechanical prosthesis with dysfunction	Pulmonary valve stenosis	Biological prosthesis without dysfunction
	Mitral mechanical prosthesis > Aortic mechanical prosthesis	No prognostic factors

AS: aortic stenosis; MS: mitral stenosis.

Concomitance of prognostic factors should be considered as worsening maternal and fetal prognosis (Table 81).^216^

**Table 81:** Conditions that worsen prognosis of pregnancy in patients with valve disease^216^

Prognostic factors: AF, PH, ventricular dysfunction, previous events (heart failure, thromboembolism, or infective endocarditis) Moderate to severe left-sided obstructive lesions Aortic diseases associated with increased diameters of the ascending aorta Marfan syndrome (diameter of the aorta > 40 mm) Bicuspid aortic valve (diameter of the aorta > 45 mm) NYHA FC III/IV Valve disease with indication of surgical or percutaneous intervention Need to anticoagulant use (transitory or permanent)

AF: atrial fibrillation; FC: functional class; NYHA: New York Heart Association; PH: pulmonary hypertension.

During pregnancy planning, keep in mind that percutaneous or surgical valve intervention should be indicated in patients with severe valve disease, even in asymptomatic patients, because NYHA FC I/II does not mean good maternal evolution in severe obstructive lesions (Table 82).^217^

**Table 82:** Recommendations for treatment in acquired native VHD, during family planning and pregnancy^217^

Valve disease	Family planning Intervention	Pregnancy		
		Maternal risk	Fetal risk	Intervention
Severe mitral stenosis MVA < 1.5 cm^2^	Consider PBMV or surgery: NYHA FC III/IV or NYHA FC I/II + SPAP > 50 mmHg or Recent onset AF	Increased risk: NYHA FC III/IV and/or AF	Prematurity Restricted intrauterine growth Fetal loss Increased if NYHA FC III/IV	Betablocker Diuretic Anticoagulation if AF If refractory maternal NYHA FC III/IV consider PBMV or surgery
Severe aortic stenosis AVA ≤ 1 cm^2^	Consider balloon valvuloplasty or surgery: Symptomatic or Asymptomatic + Altered ergometry test or LVEF < 50% or AVA < 0.7 cm^2^ mean gradient > 60 mmHg or Bicuspid valve + diameter of the aorta > 45mm	Increased risk Heart failure Arrhythmia Syncope Sudden death Aortic dissection	Complications Prematurity Restricted intrauterine growth Fetal loss	Rest Use of diuretics is controversial Consider betablocker or calcium channel blocker + Anticoagulation if AF Consider balloon valvuloplasty or surgery if heart failure or syncope
Severe mitral regurgitation	Consider surgery (repair/prosthesis): NYHA FC ≥ II or Asymptomatic + LVEF ≤ 60% + SPAP ≥ 50 mmHg + LVSD ≥ 40 mm	Heart failure AF Increased risk if LVEF < 35%	Low risk	Diuretic, vasodilator Digoxin, betablocker Consider surgery or percutaneous mitral repair if refractory heart failure
Severe aortic regurgitation	Consider surgery: Symptomatic NYHA FC ≥ II or Prognostic factors LVEF < 50% LVDD > 70 mm (75 if rheumatic) LVSD > 50 mm (55 if rheumatic) Consider intervention in proximal aorta: Isolated bicuspid valve and diameter of the aorta > 45 mm	Low risk if asymptomatic and normal LVEF Risk of heart failure if NYHA FC > II and/or AF or LVEF < 35%	Low risk	Diuretic, vasodilator, Digoxin Consider surgery if refractory heart failure Consider intervention in proximal aorta: Isolated bicuspid valve and diameter of the aorta > 45 mm

AF: atrial fibrillation; AVA: aortic valve área; PBMV: percutaneous balloon mitral valvuloplasty; FC: functional class; LVDD: left ventricular diastolic diameter; LVEF: left ventricular ejection fraction; LVSD: left ventricular systolic diameter; MVA: mitral valve area; SPAP: systolic pulmonary artery pressure.

In contrast, regurgitation lesions have better prognosis when LVEF fraction is preserved, and the rare cases with complications are those that already had surgical indication prior to pregnancy.

During pregnancy, the basic principle for prevention and treatment of complications is to prioritize general measures and to choose non-teratogenic drugs with doses adjusted to gestational age. Table 83 lists the drugs and daily doses most frequently used to control complications of valve disease during pregnancy.^218^

**Table 83:** General and pharmacological recommendations during pregnancy^218^

Restricted physical activities and low-sodium diet (4 g/day) Prophylaxis of rheumatic disease should be maintained (except sulfadiazine) If pharmacological treatment is indicated, consider: ○ Diuretic: furosemide (< 80 mg/day) ○ Betablockers: propranolol (<80 mg/day) or metoprolol succinate (< 100 mg/day), carvedilol < 50 mg ○ Non-dihydropyridine calcium channel blockers: verapamil (< 240 mg/day) ○ Vasodilator: hydralazine (< 100 mg/day) ○ Digitalis: digoxin (0.25 mg/day)

Interventional measures in valve diseases during pregnancy are reserved for cases that are refractory to clinical treatment. Percutaneous procedures should be given preference over surgery, and the proposed treatments should be discussed with the Heart Team and shared with the Obstetric Team. Balloon valvuloplasty in AS has been indicated when etiology is congenital or as an attempt to save the mother’s life in extremely severe cases. In contrast, PBMV is safe, with results equivalent to those of surgery; it nevertheless requires the classical indication criteria, such as absence of thrombus in the LA, no more than mild MR, and Wilkins-Block echocardiographic score ≤ 8.

### 19.2. Valve Prostheses

From the hemodynamic point of view, both mechanical and biological prostheses improve functional capacity, and they promote similar clinical evolution during pregnancy; nevertheless, biological prostheses appear to be more advantageous because they do not require anticoagulation (Table 84). Their limited durability, with the possibility of short-term reoperation, including during pregnancy, are the main restrictions to implantation of biological prostheses in young women.

**Table 84:** Prosthetic valve with normal function and risks to pregnancy

Biological prosthesis with normal LVEF		Mechanical prosthesis with normal LVEF	
Maternal risk	Fetal risk	Maternal risk	Fetal results
Low risk	Low risk	Intermediate risk Requires anticoagulation	High risk
Does not require anticoagulation		Systemic embolism Prosthesis thrombosis Hemorrhage	Warfarin embryopathy Fetal loss Prematurity Perinatal hemorrhage

LVEF: left ventricular ejection fraction.

The management in cases of prosthesis dysfunction during pregnancy should always prioritize the mother’s life, and the proposed treatments should be discussed with the Heart Team and shared with the Obstetric Team (Table 85).

**Table 85:** Treatment in prosthesis dysfunction during pregnancy

Biological prosthesis		Mechanical prosthesis	
Maternal risk	Fetal risk	Maternal risk	Fetal risk
Dysfunction with predominant regurgitation, NYHA FC I/II and normal LVEF Consider pharmacological measures	Low risk	Dysfunction with mild to moderate “paravalvular” regurgitation, without significant hemolysis or severe heart failure Consider pharmacological measures for heart failure and anemia Severe MR or significant hemolysis Consider intervention Heart failure and/or symptomatic hemolysis Consider percutaneous closure of the paravalvular leak or surgery (high risk of relapse)	High fetal risk, if surgery
Dysfunction with predominant valve stenosis and calcification (mitral, aortic, or tricuspid) Risks of severe heart failure, shock, sudden death Always consider percutaneous or transapical (valve-in-valve) implantation or surgery	High fetal risk Fetal loss Prematurity	Mechanical prosthesis thrombosis Consider emergency intervention (thrombolysis or surgery) Mechanical prosthesis stenosis due to intravalvular endothelial growth – pannus or mismatch Need for intervention is rare If necessary, consider surgery	High fetal risk, if surgery

FC: functional class; LVEF: left ventricular ejection fraction; MR: mitral regurgitation.

Anticoagulation regimens for patients with mechanical prosthesis remain controversial.^218^^,^^219^ To date, there are no uniform guidelines that have been widely accepted. Factors that must be considered include the following: patient preference, expertise of the attending doctor, local resources, and availability of adequate coagulation control.

The recommendations for preventing thromboembolism in mechanical prostheses are intended to meet the ideal requirements of a position based on the literature and on the authors’ experience, and they should be effective for the reality of diverse healthcare services. It is understood that the dynamics of permanent anticoagulation in patients with mechanical prostheses is multidisciplinary, and it is divided into five phases: pre-conception, each trimester, delivery, and postpartum, shown in Table 86 and Figure 11. Vigilant control of anticoagulation and doses of anticoagulants should be adjusted according to conventional targets.

**Table 86:** Anticoagulation control in patients with mechanical prosthesis during pregnancy

Gestational age (weeks)	Anticoagulant	Control
Between 6 and 12	Subcutaneous low-molecular-weight heparin 1.0 mg/kg every 12 hours or Intravenous unfractionated heparin 18 IU/kg/hour in an infusion pump (< 30,000 IU)	Anti-Xa: 0.8 to 1.2 U/ml aPTT 1.5 to 2.0 times control value
12 to 36	Warfarin, dose according to INR	Aortic INR between 2.5 and 3.0 Mitral INR between 3.0 and 3.5
After 36, until delivery	Low-molecular-weight heparin 1.0 mg/kg subcutaneous every 12 hours or Intravenous unfractionated heparin 18 IU/kg/hour in an infusion pump (< 30,000 IU)	Anti-Xa: 0.8-1.2 U/ml aPTT 1.5 to 2.0 times control value
Postpartum	Subcutaneous low-molecular-weight heparin 1.0 mg/kg every 12 hours Intravenous unfractionated heparin 18 IU/kg/hour in an infusion pump (< 30,000 IU) Warfarin must reach target INR before hospital discharge	Anti-Xa: 0.8 – 1.2 U/ml aPTT 1.5 to 2.0 times control value INR between 2.0 and 2.5

aPTT: activated partial thromboplastin time; INR: international normalized ratio.

**Figura 11:** Flowchart with recommendations for anticoagulation in patients with mechanical prostheses during pregnancy, delivery, and postpartum. βHCG: beta-human chorionic gonadotropin; IV UFH: intravenous unfractionated heparin; SC LMWH: subcutaneous low-molecular-weight heparin; VKA: vitamin K antagonist.

Phase 1 - *orientations regarding early diagnosis of pregnancy:* Clarify that it is mandatory to maintain anticoagulation and discuss the availability of anticoagulants and their risks during all phases of pregnancy, delivery, and postpartum. Advice includes information regarding the importance of early diagnosis of pregnancy in order to reduce the occurrence of embryopathy, which occurs between the sixth and ninth week of pregnancy. During this consultation, the patient receives a request for beta-human chorionic gonadotropin (βHCG) measurement, which should take place as soon as there are doubts regarding late menstruation.

Phase 2 - *first trimester:* Once pregnancy has been confirmed (βHCG and obstetric ultrasound), warfarin should be substituted by heparin which makes it possible to balance between the benefit of preventing maternal thrombosis and the harm of embryopathy. In patients whose first medical consultation occurs after the sixth week of gestation, warfarin should not be suspended. The couple should be informed that there is a possibility of embryopathy and that the risks of substituting warfarin for heparin are no longer justified.

Phase 3 - *second trimester:* Return to oral anticoagulant. The return to warfarin is based on the benefit of shortening the use of heparin and lowering the risk of embryopathy. The proposal is to maintain the warfarin dosage in accordance with pre-pregnancy goals, with weekly or biweekly INR control. Reintroduction of warfarin should take place simultaneously with the use of subcutaneous low-molecular-weight heparin or intravenous unfractionated heparin until the target INR has been reached.

Phase 4 - *third trimester:* Consider hospitalization, return to parenteral anticoagulation and schedule delivery. Hospitalization should be scheduled at week 36 of pregnancy for use of subcutaneous low-molecular-weight heparin or intravenous unfractionated heparin.

Phase 5 - *postpartum:* Reintroduction of oral anticoagulation and hospital discharge. Six hours after delivery, if there are not maternal complications, intravenous unfractionated heparin or subcutaneous low-molecular-weight heparin should be reintroduced in therapeutic doses. Warfarin should be prescribed 48 hours after delivery, following the transition dynamic in conjunction with heparin until the INR value of 2.0 has been reached, at which point the patient is discharged from the hospital.

### 19.3. Delivery and Postpartum

Delivery planning should be multidisciplinary, starting at week 34 of pregnancy. Vaginal delivery is considered to be more advantageous because it is associated with less blood loss and lower risks of thrombosis and infection. Sequential anesthesia techniques, with neuraxial anesthesia, have hemodynamic advantages because they allow a gradual form of sympathetic block. In general, cases of maternal indication for cesarean delivery require general anesthesia (Table 87).

**Table 87:** Recommendations for route of delivery and anesthesia in patients with valve disease

Vaginal birth and spinal epidural anesthesia are preferable in cases with low- and intermediate-risk valve disease Cesarean delivery should be considered in the event of: High-risk valve disease (severe obstructive lesions) Diseases of the thoracic ascending aorta Delivery under anticoagulation History of aortic dissection Antibiotic prophylaxis at the moment of delivery is no longer routine. Nevertheless, it may be considered in patients with valve prostheses or history of infective endocarditis: Ampicillin 2.0 g intravenous + gentamicin 1.5 mg/kg/day intramuscular, one hour before delivery There are no restrictions with respect to breastfeeding

### 19.4. Contraception

The choice of contraceptive method for women with valve diseases requires multidisciplinary effort, involving the gynecologist and the cardiologist, in order to seek safety, efficacy, tolerance, and easy access. Accordingly, guidelines for prescription should be based on the Contraceptive Eligibility Criteria, which classify contraceptives in four risk categories, and on the Pearl index, which calculates the effectiveness of a method considering the number of pregnancies per 100 women during the first year of use.^220^^,^^221^ For patients with valve disease, the current tendency is to indicate methods that contain only progesterone or combinations of progesterone and natural estrogen in monthly injecTable forms, because they are safe, effective, and easily accessible (Table 88). Although intrauterine devices are classified as category 2, they have not been indicated in patients with valve diseases, due to the presumed inherent risk of IE.

**Table 88:** Medical eligibility criteria (modified)* and index of effectiveness for contraceptive use in patients with valve disease^220^^,^^221^

Available contraceptives	Oral CHC	Monthly injection	Progesterone pills	InjecTable progesterone	Implantation of progesterone	Copper IUD	Levonorgestrel IUD
Valve disease							
Not complicated	2	1	1	1	1	3/4	3/4
Prognostic factors	4	4	1	1	1	4	4
Effectiveness	8	3	3	3	0.05	0.8	0.1

* Prognostic factors: Effectiveness (Pearl Index) calculated as the number of pregnancies per 100 women who routinely use the method. Eligibility criteria: category 1: there are no restrictions to using the method; category 2: the advantages of using the method generally outweigh the theoretical or proven risks; category 3: the theoretical or generally proven risks outweigh the advantages of using the method; category 4: condition that represents an unaccepTable health risk of using the contraceptive method. CHC: combined hormonal contraceptive; IUD: intrauterine device.

## List of Abbreviations:

βHCG: beta-human chorionic gonadotropin
ACC/AHA: American College of Cardiology/American Heart Association
AF: atrial fibrillation
AR: aortic regurgitation
aPTT: activated partial thromboplastin time
AS: aortic stenosis
ASA: acetylsalicylic acid
AVA: aortic valve area
PBMV: percutaneous balloon mitral valvuloplasty
BNP: brain natriuretic peptide
PBTV: percutaneous balloon tricuspid valvuloplasty
CHC: combined hormonal contraceptive
DOACs: direct oral anticoagulants
ECG: electrocardiogram
EOA: effective orifice area
EROA: effective regurgitant orifice area
ESC/EACTS: European Society of Cardiology/European Association for Cardiothoracic Surgery
FC: functional class
IE: infective endocarditis
INR: international normalized ratio
IUD: intrauterine device
LA: left atrium
LV: left ventricle
LVDD: left ventricular diastolic diameter
LVEF: left ventricular ejection fraction
LVSD: left ventricular systolic diameter
MAC: mitral annulus calcification
MR: mitral regurgitation
MS: mitral stenosis
MVA: mitral valve area
NYHA: New York Heart Association
PH: pulmonary hypertension
PHT: pressure half time
RA: right atrium
RF: rheumatic fever
rTPA: recombinant tissue plasminogen activator
SBC: Sociedade Brasileira de Cardiologia (Brazilian Society of Cardiology)
SPAP: systolic pulmonary artery pressure
STS: Society of Thoracic Surgeons
TAVI: transcatheter aortic valve implantation
TR: tricuspid regurgitation
TS: tricuspid stenosis
TTR: time in therapeutic range
VHD: valvular heart disease
VKA: vitamin K antagonists
